# Evidence of two deeply divergent co-existing mitochondrial genomes in the Tuatara reveals an extremely complex genomic organization

**DOI:** 10.1038/s42003-020-01639-0

**Published:** 2021-01-29

**Authors:** J. Robert Macey, Stephan Pabinger, Charles G. Barbieri, Ella S. Buring, Vanessa L. Gonzalez, Daniel G. Mulcahy, Dustin P. DeMeo, Lara Urban, Paul M. Hime, Stefan Prost, Aaron N. Elliott, Neil J. Gemmell

**Affiliations:** 1 Peralta Genomics Institute, Chancellor’s Office, Peralta Community College District, 333 East 8th Street, Oakland, CA 94606 USA; 2 AIT Austrian Institute of Technology, Center for Health and Bioresources, Molecular Diagnostics, Giefinggasse 4, 1210 Vienna, Austria; 3 Global Genome Initiative, National Museum of Natural History, Smithsonian Institution, 1000 Constitution Ave., Washington, DC 20560 USA; 4 Department of Anatomy, University of Otago, PO Box 913, Dunedin, 9054 New Zealand; 5 Biodiversity Institute and Natural History Museum, University of Kansas, 1345 Jayhawk Blvd., Lawrence, KS 66045 USA; 6 LOEWE-Center for Translational Biodiversity Genomics, Senckenberg Museum, 60325 Frankfurt, Germany; 7 South African National Biodiversity Institute, National Zoological Garden, Pretoria, 0184 South Africa

**Keywords:** Zoology, Genomics

## Abstract

Animal mitochondrial genomic polymorphism occurs as low-level mitochondrial heteroplasmy and deeply divergent co-existing molecules. The latter is rare, known only in bivalvian mollusks. Here we show two deeply divergent co-existing mt-genomes in a vertebrate through genomic sequencing of the Tuatara (*Sphenodon punctatus*), the sole-representative of an ancient reptilian Order. The two molecules, revealed using a combination of short-read and long-read sequencing technologies, differ by 10.4% nucleotide divergence. A single long-read covers an entire mt-molecule for both strands. Phylogenetic analyses suggest a 7–8 million-year divergence between genomes. Contrary to earlier reports, all 37 genes typical of animal mitochondria, with drastic gene rearrangements, are confirmed for both mt-genomes. Also unique to vertebrates, concerted evolution drives three near-identical putative Control Region non-coding blocks. Evidence of positive selection at sites linked to metabolically important transmembrane regions of encoded proteins suggests these two mt-genomes may confer an adaptive advantage for an unusually cold-tolerant reptile.

## Abstract (web summary)

Robert Macey et al. use deep sequencing to reveal two co-existing, deeply divergent, fully functional mitochondrial genomes in a single Tuatara from Lady Alice Island, New Zealand. This finding will stimulate further investigation, having profound implications for our understanding of mitochondrial evolution and function.

## Introduction

Mitochondrial polymorphisms in animals exist as low-level mitochondrial heteroplasmy and as deeply divergent co-existing mitochondrial genomes. Low-level mitochondrial heteroplasmy typically arises via shallow historical mutation with the maternal passage leading to generational fixation^1,2^. Alternative mechanisms are mitochondrial degradation forming heteroplasmy^2,3^ that is generally not inherited and paternal leakage^4^. Instances of paternal leakage causing apparent heteroplasmy have been suggested to be mtDNA fragments recently integrated into the nuclear genome^4^. Deeply divergent co-existing mt-genomes are previously only known among bivalvian mollusks, where one mt-genome is typically maternally inherited and the other paternally inherited, a phenomenon known as doubly uniparental inheritance (DUI)^5^.

**Fig. 1:**
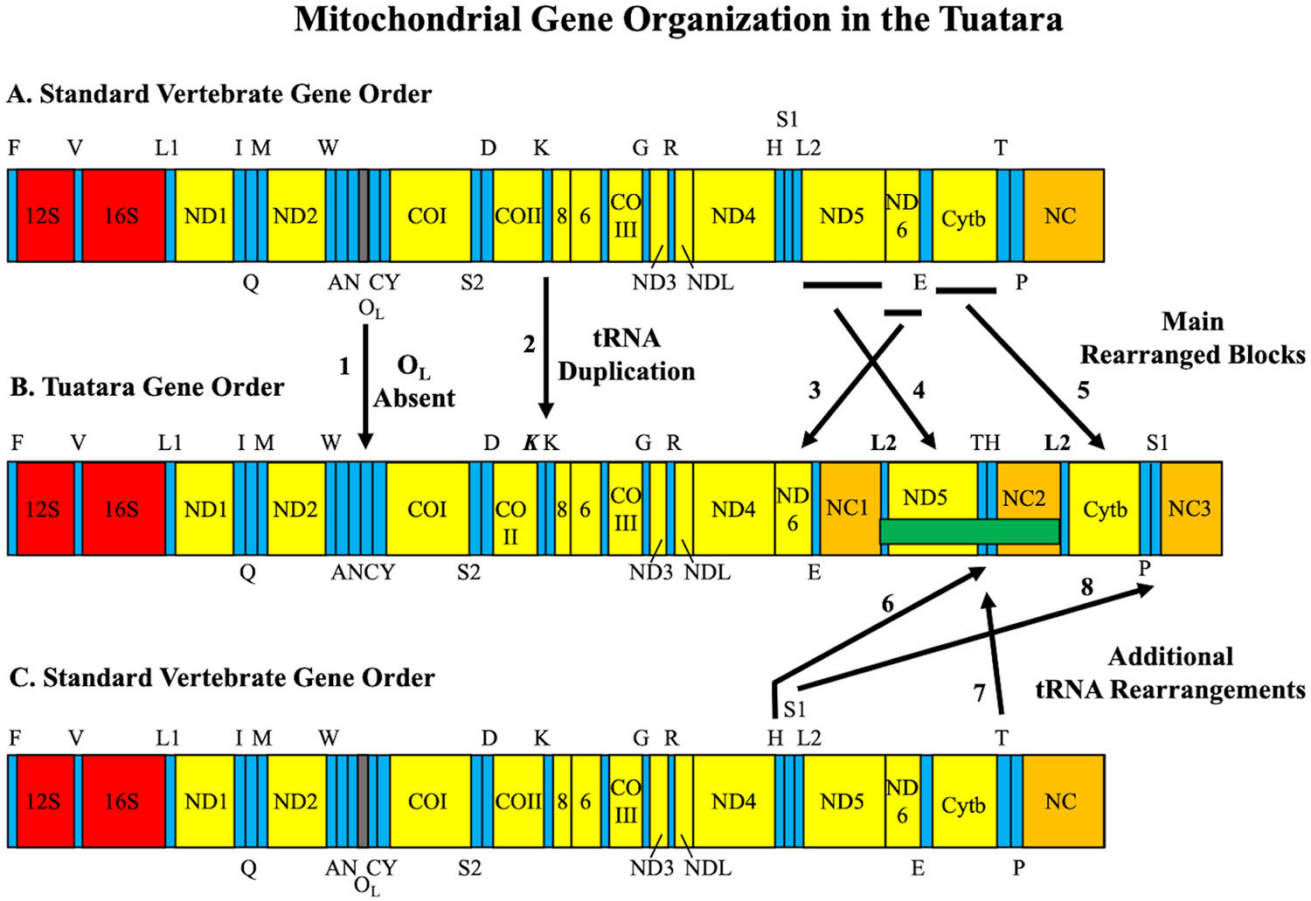
Mitochondrial gene organization in the Tuatara. Arrangements are depicted as a linear bar for what is a circular molecule. **A** The standard vertebrate mt-gene order presumptive as ancestral among vertebrates. **B** The mt-gene order discovered in complete mt-genomes of the Tuatara. **C** The standard vertebrate mt-gene order as in “**A**” above. Abbreviations for genes are: 12 S and 16 S for small and large ribosomal RNAs; NDl–6 and 4 L for NADH dehydrogenase subunits; COI–III for cytochrome *c* oxidase subunits; Cytb for cytochrome *b*; 6 and 8 for ATPase subunits. All protein-coding genes are transcribed from the heavy strand with the exception of *ND6* from the light strand. Transfer RNA genes are represented by their standard one-letter amino acid codes positioned on the strand encoded, with the top representing the heavy strand and the bottom representing the light strand. S1, S2, Ll, and L2 depict *tRNA*^*Ser(AGY)*^*, tRNA*^*Ser(UCN)*^*, tRNA*^*Leu(UUR)*^, and *tRNA*^*Leu(CUN)*^, respectively. The identical and duplicated *tRNA*^*Leu(CUN)*^ is labeled L2. The Control Region (O_H_), responsible for heavy-strand replication, standardly has one copy among vertebrates. Identified are three non-coding regions labeled NC1–3 and putative Control Regions. Black bars indicate protein-coding regions rearranged relative to the standard vertebrate mt-genic organization. Eight novel features of the Tuatara mt-genome are numbered with arrows. (1) The origin of light-strand replication (O_L_) is not present in the Tuatara^19^ but a stem-and-loop structure overlapping tRNA genes may supplement (Fig. 4), which is indicated by an arrow. (2) The bold italic *K* represents a duplicated *tRNA*^*Lys*^ that appears to be a pseudogene^8^. (3) *ND6* and *tRNA*^*Glu*^ are moved relative to the standard vertebrate mt-genic organization. (4) *tRNA*^*Leu(CUN)*^ and *ND5* are moved relative to the standard vertebrate mt-genic organization. (5) *Cytb* is moved relative to the standard vertebrate mt-genic organization. (6) *tRNA*^*His*^ is moved relative to the standard vertebrate mt-genic organization. (7) *tRNA*^*Thr*^ is moved relative to the standard vertebrate mt-genic organization. (8) *tRNA*^*Ser(AGY)*^ is moved relative to the standard vertebrate mt-genic organization. The two identical or near identical copies of *tRNA*^*Leu(CUN)*^ flank the missing region of all previously reported Tuatara mt-genomes^5–7^ (horizontal green bar).

The vertebrate mt-genome is small in size (15–26 kb) with most taxa sharing a common gene order^6^. Among the more structurally complicated mt-genomes described is that of the Tuatara (*Sphenodon punctatus*, Fig. 1). A highly threatened reptile, the Tuatara remains on 32 small islands in New Zealand, and represents the sole-surviving taxon of a deep vertebrate lineage (Order: Rhynchocephalia), which diverged from squamate reptiles 220–250 million years ago (MYA)^7^. Previous studies reporting the first ‘complete’ mt-genome^8^ and subsequent 34 additional population-level Tuatara mt-genomes^9,10^ suggests the Tuatara mt-genome is missing three genes that encode ND5, tRNA^His^, and tRNA^Thr^. That finding is striking because absent tRNA genes have transcriptional implications for all mt-encoded proteins^11^.

In order to investigate the true Tuatara mt-genome composition an array of sequencing techniques including Illumina whole-genome shotgun, Oxford Nanopore, PacBio, and PCR-based Sanger sequencing are used on individuals sampled across extremes of the Tuatara’s geographic distribution: Stephens Island (SI-1–4) and Lady Alice Island (LAI) samples, the latter sequenced for a whole-genome project^12^. In doing so, two deeply divergent mt-molecules are discovered in the Tuatara (LAI), each containing the three genes previously reported as missing, albeit bounded by a series of repeated Control Region copies. The Tuatara is exceptional in being a large-bodied reptile metabolizing in cold environments, in which mitochondrial ATP synthesis is conducted^13,14^. The likely time of divergence among these molecules and the selective pressures that may have led to maintain two deeply divergent, coexisting mitochondrial genomes is explored.

## Results

### The discovery of two mitochondrial genomes in the Tuatara

**Fig. 2:**
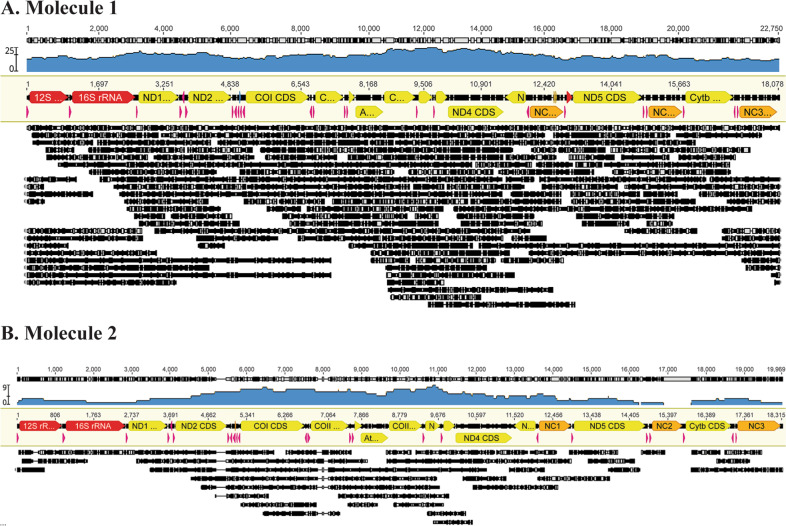
Oxford Nanopore coverage of Lady Alice Island mt-genomes (M1 and M2). Mapped Oxford Nanopore reads to the Illumina assembly of mt-genomes. Blue graphs represent mapping coverage. Gene annotations are shown below the coverage map (some gene names are truncated with the same gene order reported herein). **A** Molecule 1, with 100% of reference covered, averaging 2504.5 bp in length (std dev = 2409.2; min = 514; max = 16,978) with an average depth coverage of 16.9 (std dev = 3.4; min = 11; max = 25). The uppermost read covers the entire molecule. **B** Molecule 2, with 95.8% of reference covered, averaging 1990.2 bp in length (std dev = 1757.6; min = 551; max = 7026) with an average depth coverage of 3.7 (std dev = 3.4; min = 11; max = 25). Light gray arrows on the ends indicate reads that wrap around the molecules. See Supplementary Data 1 for a fasta file of LAI M1 Oxford Nanopore reads and Supplementary Data 2 for a fasta file of LAI M2 Oxford Nanopore reads, each a filtered product and mapped here.

The discovery of two mt-genomes in the LAI individual is established via complementary Illumina (high coverage short-read) and Oxford Nanopore (long-read) data that identify two groups of DNA sequencing reads distinguishable by >5% sequence divergence. Illumina assembly of the LAI molecule 1 (M1) is 18,078 bases and molecule 2 (M2) 18,315 bases, each containing all 37 genes typical of animal mt-genomes. Molecular features implicate both genomes as mtDNA and not nuclear DNA. These include strong strand-bias against guanine (13.8% M1 and 14.6% M2; Supplementary Note 1), all protein-coding genes translate with no internal stop codons, tRNA genes encode tRNAs with stable secondary structures containing recognized anti-codons^6^, and no sequencing reads, whether short or long-read data, were flanked by nuclear DNA. The two mt-genomes are further confirmed via Oxford Nanopore sequencing of LAI total genomic DNA producing 9.46 Gb of sequence data in 7,229.48 K reads (Fig. 2, Table 1, and Supplementary Note 2). Filtering out mtDNA reads with assignments to M1 or M2, results in 114 mtDNA reads that map to 100% of the LAI M1 Illumina assembly reference. Thirty-two LAI M2 assigned reads map to 95.8% of the LAI M2 Illumina assembly reference. A single complete mt-genomic Oxford Nanopore read is obtained for LAI M1 from an mt-molecule naturally nicked, with both native DNA complement strands end-connected via laboratory manipulation, and sequenced in a single reaction (Supplementary Note 3 and Supplementary Fig. 1).

**Table 1:** Data obtained from Oxford Nanopore runs both mined and retrieved mitochondrial DNA (see text for protocol information).

Run	Modification to library preparation	Chemistry	Total # of reads (K)	Total yield (Gb)	mt-reads >500 bp	mtDNA yield (kb)	Mapped molecule 1 mt-reads	Mapped molecule 1 yield (kb)	Mapped molecule 2 mt-reads	Mapped molecule 2 yield (kb)
1	10 kb shear	1D, R9.4, SQK-108	165.43	0.99	7	25.31	7	25.31	0	0
2	10 kb shear, Trypsin	1D, R9.4, SQK-108	353.58	2.06	16	94.42	9	43.87	5	24.54
3	2D	2D, R9.4, SQK-208	18.79	0.26	10	27.83	9	45.02	0	0
4	5 kb shear	1D, R9.4, SQK-108	34.64	0.31	0	0	0	0	0	0
5	Trypsin	1D, R9.4, SQK-208	265.17	0.98	8	46.46	6	14.16	1	4.836
6	Trypsin	1D, R9.4, SQK-108	18.31	0.11	1	3.43	1	3.39	0	0
7	Trypsin	1D, R9.4, LSK109	1224.00	1.30	36	74.99	31	64.60	4	8.22
8	Trypsin	1D, R9.4, LSK109	5149.57	3.45	80	112.71	51	74.69	22	26.09
Totals			7229.48	9.46	158	385.15	114	271.04	32	63.69

Run numbers are described in the text: library preparation methods refer to variations in protocols described in the methods section, chemistry is the type of sequencing run and the version of flow cell used. Run numbers 1–3 are SRA run numbers 1–3, run number 4 is SRA run number 5, run number 5 is SRA run number 6, run number 6 is SRA run number 10, run number 7 is SRA run number 14, and run number 8 is SRA run number 15.

### A highly divergent second mt-genome via phylogenetics

**Fig. 3:**
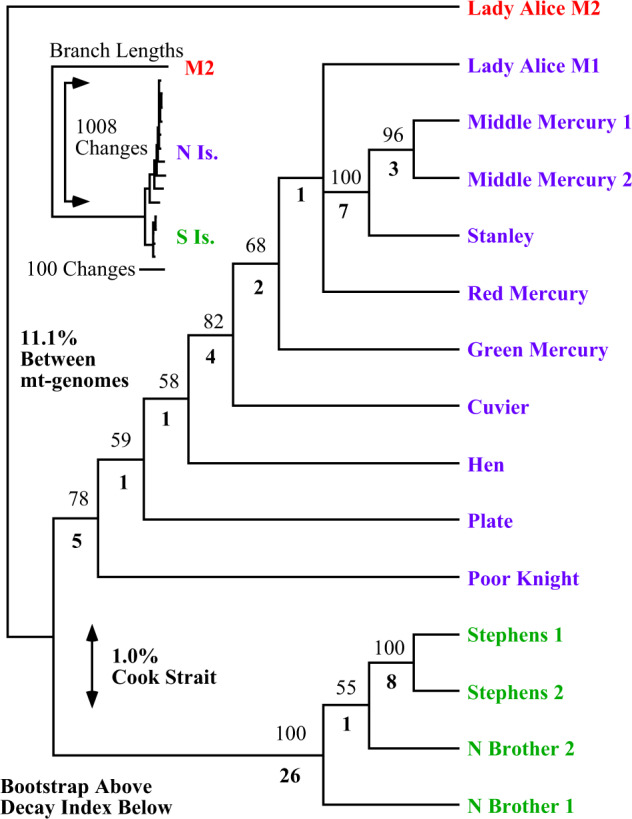
Phylogenetic relationships of Tuatara populations in relation to Lady Alice Island molecules (M1 and M2). Analysis of 12 mt-protein-coding genes from published data^9,10^ not including *ND5* in relation to both M1 and M2 sequences from LAI reported here. Bootstraps are depicted above with decay indices below. The tree has a length of 1302 steps from analysis of 9522 aligned sites of which 170 are parsimony informative; the entire tree collapses in 26 steps, hence the decay index below branches. Using LAI M2 as a root, it is implicated it is placed between northern and southern populations forming two groups separating in the Cook Strait. These northern and southern groups have only 1.0% sequence divergence between them, while sequence divergence to LAI M2 is 11.1%. The upper left corner shows relative branch lengths with the LAI M2 sample considerably different in nucleotide changes. Phylogram depiction of the shortest tree illustrates branch length DNA changes along the shortest path.

Phylogenetic analysis of protein-coding sequences from LAI M1 and M2 with published^9,10^ mt-genome sequences (lacking *ND5*) places LAI M2 outside all extant populations (Fig. 3). The rooting position of M2 between northern and southern populations forms two groups of M1 separated by the Cook Strait with 1.0% sequence divergence. In contrast, all M1 sequences are on average 11.1% divergent to M2, further negating the possibility of M2 being a recent nuclear-integrated copy. Monophyly of LAI genomes (M1 and M2) is statistically rejected. The well-dated *ND1–COI* section in amphibians and reptiles is estimated to have a pair-wise sequence divergence of 1.3% per million years^15–17^. Application of this divergence rate to the Tuatara mt-genomes indicates a 7.8-million-year separation between M1 and M2 (10.1% sequence divergence), with a 1.2-million-year separation between northern and southern population M1 genomes (1.6% average sequence divergence), indicating a deeply divergent second mt-molecule (Supplementary Note 4).

### An extremely divergent genomic organization among vertebrates

The most complex and rearranged vertebrate mt-genome is discovered in the Tuatara. All 37 mt-genes (13 protein-coding, 2 rRNA, and 22 tRNA) are present with the addition of three putative Control Region non-coding blocks (NC1–3), and duplicate *tRNA*^*Lys*^ and *tRNA*^*Leu(CUN)*^ copies (Fig. 1). A newly identified segment containing *ND5, tRNA*^*Thr*^*, tRNA*^*His*^, NC2, and *tRNA*^*Leu(CUN)*^ second copy is discovered that was not reported in all 35 previously published Tuatara mt-genomes^8–10^. In contrast to the standard vertebrate mt-gene order, the section between *ND4* and *tRNA*^*Phe*^ contains the highly rearranged section of *ND6, tRNA*^*Glu*^, NC1, *tRNA*^*Leu(CUN)*^ first copy, *ND5, tRNA*^*Thr*^*, tRNA*^*His*^, NC2, *tRNA*^*Leu(CUN)*^ second copy, *Cytb, tRNA*^*Pro*^*, tRNA*^*Ser(AGY)*^, and NC3. This large-scale regional genic rearrangement with NC duplications involves protein-coding genes and numerous tRNA gene movements that cannot be explained by a simple duplication and deletion of redundant sequence model^6^. The Tuatara mt-genome architecture is also confirmed in a PacBio sequenced individual (SI-3) and our mining of a transcriptome library^18^ (SI-4), Sanger sequencing of PCR-amplified products from SI-2 and SI-3 with forward and reverse primers extending inside *ND5* further support that gene and other gene junctions are in the mt-genome (Supplementary Note 5).

### Unusual replication origins

**Fig. 4:**
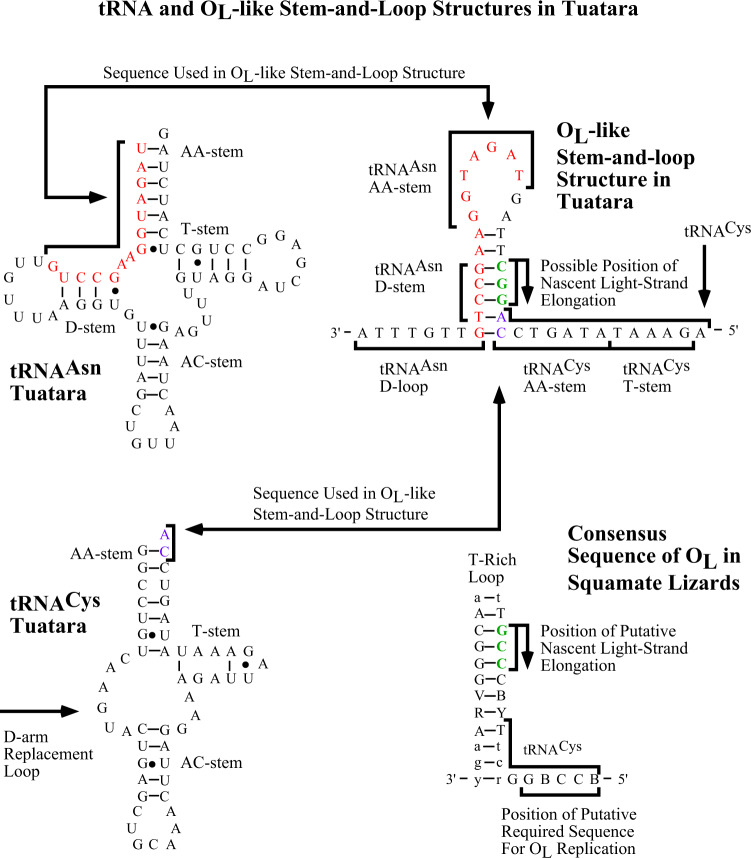
Secondary structures in the region of the light strand replication origin (O_L_) in the Tuatara (*Sphenodon punctatus*). Top right is a stem-and-loop structure that may serve as O_L_ but is highly unusual (presented as heavy-strand template DNA sequence). This O_L_-like structure overlaps 14 bases with *tRNA*^*Asn*^ (normally no overlap) and two bases with *tRNA*^*Cys*^ (normally two to four), (see arrows and lower right for O_L_ comparison). Putative light-strand encoded tRNAs are presented to the left and are the same sequence as heavy-strand DNA except for the use of U instead of T. The Tuatara has an unusual *tRNA*^*Cys*^ that lacks a D-stem and instead contains a D-arm replacement loop (arrow)^19,20^. Stems are as follows: AA = amino-acid acceptor stem, D = dihydrouridine stem, AC = anticodon stem, and T = TphiC stem. Consensus heavy-strand sequence of putative O_L_ from squamate lizards is presented in the lower right. This sequence is based on representatives from the Iguanidae (nine genera), Gekkonidae (*Teratoscincus*), Dibamidae (*Dibamus*), Lacertidae (*Eremias*), Teiidae (*Cnemidophorus*), Cordylidae (*Platysaurus*), Anguidae (*Elgaria*), Xenosauridae (*Xenosuurus*), and Varanidae (*Varanus*);^6,54^ *Dibamus novaeguineae*, U71327, was previously reported as *Lialis jicari*^6^. Bases in capitals are conserved pairings and downstream sequences. Bases in lower case are often paired. Variable positions are labeled with their standard one-letter codes: R = G or A; Y = C or T; B = G, C, or T; and V = G, C, or A. The 3’-GCC-5’ heavy-strand template sequence identified as the point of light-strand elongation in mouse^55^ is indicated in green font (arrow). The heavy-strand sequence 3’-GBCCB-5’ in *tRNA*^*Cys*^ related to the downstream 3’-GGCCG-5’ sequence found to be required for in vitro replication in humans^56^ is underlined. Note the O_L_-like stem-and-loop structure in the Tuatara (upper right) has a G-C rich heavy-strand sequence of 3’-CGG-5’ (bases in bold and arrow), which is the complement of the 3’-GCC-5’ heavy-strand template sequence identified as the point of light-strand elongation in mouse^55^. No obvious sequence related to the downstream 3’-GGCCG-5’ sequence found to be required for in vitro replication in humans^56^ is identifiable in the Tuatara.

**Fig. 5:**
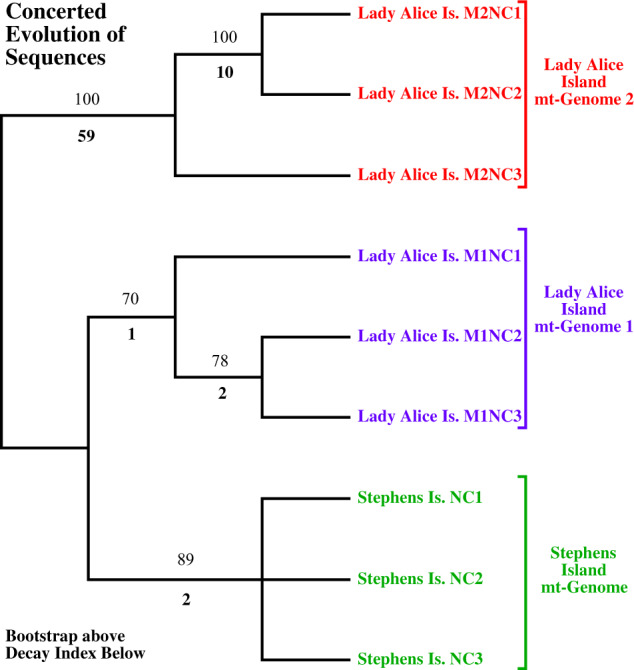
Phylogenetic tree depicting relationships among non-coding blocks (NC1–3) using Lady Alice Island M1 and M2 with Stephans Island sample 4. Strict consensus of two equally parsimonious trees with lengths of 107 steps from analysis of 737 aligned sites of which 80 are parsimony informative. Duplicated regions within an mt-genome cluster, as opposed to all NC1 sequences grouping, all NC2 sequences grouping, and all NC3 sequences grouping; a result consistent with concerted evolution of sequences in which identical or near identical sequences in repeated regions are propagated. Bootstraps are presented above and decay indices are depicted below.

The replication origin for the light strand (O_L_), missing between *tRNA*^*Asn*^ and *tRNA*^*Cys*^^6,19^, has an O_L_-like structure overlapping 14 bases with *tRNA*^*Asn*^ (normally none) and two bases with *tRNA*^*Cys*^ (normally two to four). An associated structure of the adjacent *tRNA*^*Cys*^ encoding a tRNA lacking a D-arm that instead contains a D-arm replacement loop^19,20^ may provide an alternative O_L_ initiation site^6^ (Fig. 4). Concerted evolution of two Control Region sequences in which evolution of duplicated segments of DNA undergo rapid replacement during replication to either create identical or near identical copies through time^21^ is observed in several independent reptiles lineages^22–24^. The Tuatara three non-coding blocks (NC1–3) show features consistent with Control Region copies that are nearly identical suggestive of concerted evolution (Supplementary Note 6). Phylogenetic analysis of duplicated non-coding blocks of the two LAI mt-genomes and the SI-4 mt-genome produces two equally parsimonious trees showing duplicated regions within an mt-genome clustering (Fig. 5). Monophyly of NC1, NC2 or NC3 sequences is each statistically rejected, a result consistent with concerted evolution in which identical or near identical sequences in repeated regions are propagated^21^.

### Inheritance of two mt-genomes

The LAI individual exhibiting two mt-genomes (M1, M2) is a male, which received genome-wide sequencing efforts^12^, while all other samples were female and not as extensively sequenced. Mined Illumina mtDNA reads totaling 209,650 from LAI, identify 30,005 as M2, and 176,980 as M1, resulting in 206,985 reads assignable to either M1 or M2. Thus, M2 is represented by 14.5% (1/7^th^ concentration) of assignable reads, relative to the dominant M1 copy. Among bivalves, males inherit and carry mtDNA from both parents, while females only carry mtDNA from the mother^5^. In males, somatic tissues are variable for the concentration of the male-type mt-genome within individuals and between species^25^, but generally in low concentration in somatic tissues^5,26^. A male inherited Tuatara copy is possible. The Tuatara M2 genome is detected only in the single male sampled and DNA was extracted from blood. No other male samples were available, and the M2 genome was not detected in any females sampled, although not subjected to genome-level sequencing.

### Structural content of amino acids in two molecules

**Fig. 6:**
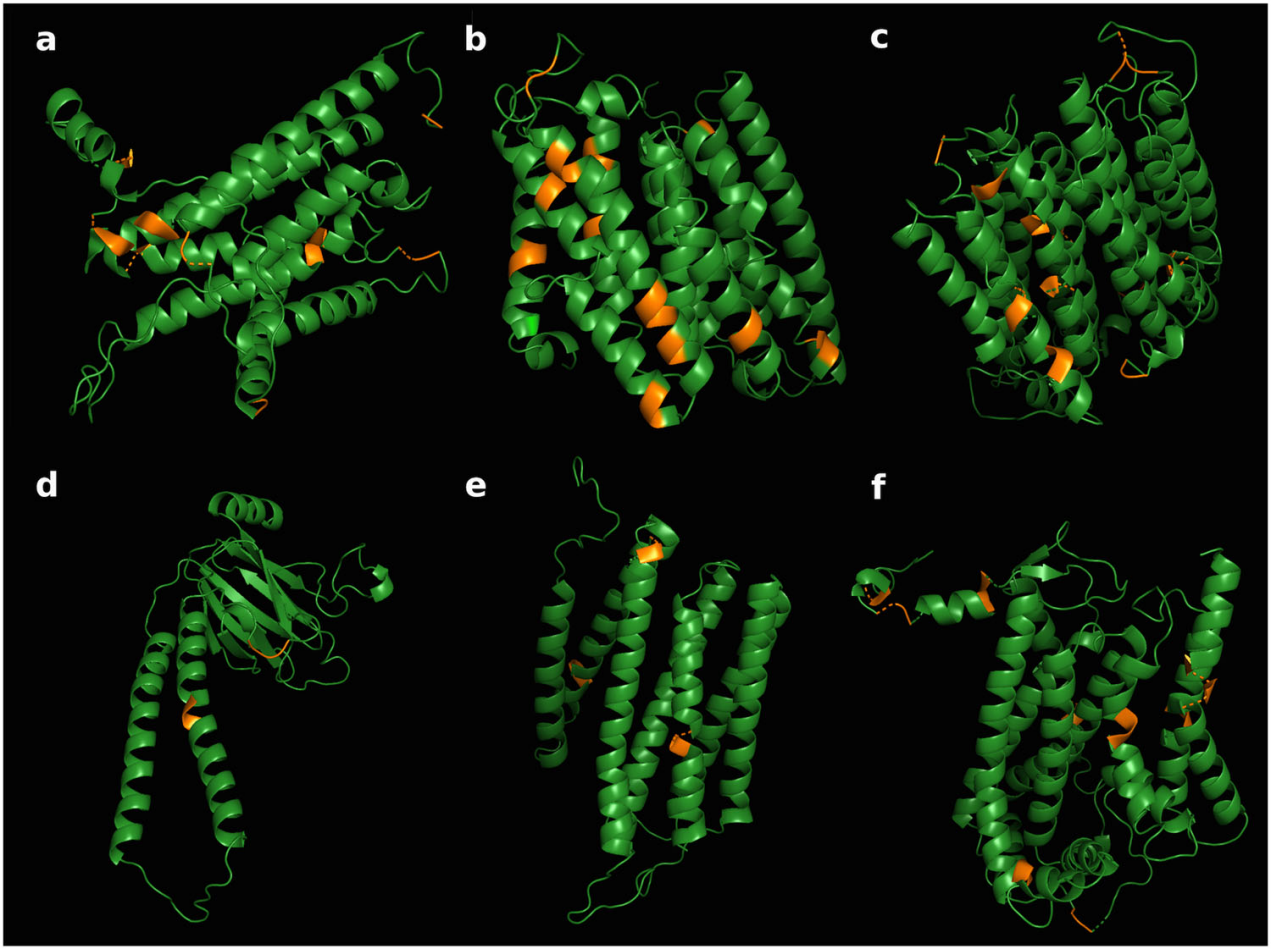
Positive selection of amino acids in protein structure. Protein structure of LAI M2 proteins depicting putative positive selection (orange) in encoded **A** ND1, **B** ND2, **C** ND4, **D** COII, **E** COIII, and **F** Cytb. Structures are estimated with the ConSurf method^51^ based on homologs of proteins in the UNIREF-90 database, and visualized using PyMOL. Amino acid changes under putative positive selection in LAI M2 as detected by PAML and FEL are colored in orange, with the majority of them residing in transmembrane regions.

The Tuatara is a large-bodied unusually cold-tolerant reptile with a low standard metabolic rate (13 °C)^13,14^. Two co-occurring mt-genomes may therefore be advantageous for metabolic flexibility in cool environments. Adaptations in transmembrane proteins have been shown to have a high bearing on environmental fitness in extreme environments^27^. Selection analysis of codon positions with PAML (Supplementary Table 1) identifies a consistent pattern of purifying selection in all mitochondrial-encoded proteins, with an average ratio of non-synonymous to synonymous changes of *ω* = 0.096 ± 0.016 (Supplementary Table 2). However, PAML and the fixed effects approach in FEL detect a series of amino acids under putative positive selection in LAI M2 and show that the majority of them lie in transmembrane regions. A concentration of 43 out of 55 detected amino acid sites under putative positive selection (78%) reside in transmembrane regions of encoded ND1, ND2, ND4, COII, COIII, and Cytb (FDR ≤ 0.05; Fig. 6; Supplementary Table 3), contrasting typical patterns of mammalian evolution with strong enrichment of adaptive variation in loop regions^28^. As amino acid changes in transmembrane regions have a stronger impact on protein function than in loop regions^29^, the selective enrichment of transmembrane amino acid changes suggests divergence of molecules in relation to the species’ life history. Further work is needed to understand the biological implications of two deeply divergent Tuatara mt-genomes.

## Discussion

The discovery of two deeply-divergent mt-genomes in the Tuatara has profound implications for our understanding of animal mitochondrial genome organization, inheritance, and evolution. The Tuatara is the sole surviving member of an ancient vertebrate lineage (Rhynchocephalia) providing a long period of isolated evolution (220–250 million years)^7^. Whether duplication of Tuatara mt-genomes arose via uni- or biparental mechanisms, and how they have been maintained for a 7–8 million-year period through a potential adaptive advantage is a dynamic new area for research.

DUI is the only well-studied mode of co-existing, deeply divergent mt-molecules found in single individuals, and thus far only confirmed in bivalvian mollusks^5^. In mollusks, the maternal copy is present in somatic tissues, whereas the paternal copy is found primarily in germ-line cells^5,26^. Our discovery of two deeply divergent (10.4% sequence divergence) mt-genomes in the Tuatara is intriguing, but the origin, inheritance mechanism, and maintenance of the two molecules remains unknown. Thus, for now, our results do not conclusively support, nor refute a DUI hypothesis.

The two divergent mt-genomes were identified in a single male (the second mt-molecule at 1/7^th^ the concentration of the first) and their discovery was only achievable in combination with genome-wide sequencing^12^ of DNA from blood. Additional samples were screened for a second mt-genome via long-read PacBio sequencing and molecule-specific PCR primers, yet were unsuccessful. These additional individuals were female, high molecular weight DNA extracted from liver was used, and these samples were not subjected to the same exhaustive genome-scale sequencing as our male sample. Additionally, the published transcriptome library^18^ was consolidated from multiple individual embryos sequenced at modest depth, and no signal of a second mt-genome was herein detected. The lack of detection in female samples could be attributable to simple unsuccessful attempts of PCR-primer specificity/preferential annealing and/or non-exhaustive genomic sequencing efforts.

Future studies seeking to confirm the widespread presence of two mt-molecules in the Tuatara should examine multiple individuals, of both sexes, across the geographic range of the Tuatara. Ideally, studies would be conducted in pedigrees to explore the inheritance of these molecules, and across multiple tissues to explore any tissue-specific patterns. Such a study is not trivial for a species that is protected under New Zealand and international law, and that is a taonga, or special treasure for those Maori iwi that are the kaitiaki, guardians, of the Tuatara. Our genome sequencing work was undertaken in partnership with Ngatiwai iwi, but any future studies in other locations would require further partnerships with Ngatiwai and another iwi across Aotearoa New Zealand to gain permissions to work on, sample, and sequence these taonga.

Tuatara mt-genomes show extreme rearrangement relative to other vertebrate mt-genomes. Tuatara mt-gene rearrangements include tRNA genes often found in mt-genomic rearrangements, but in the Tuatara with drastic distant locations relative to the standard vertebrate gene order^6^. Unlike most rearrangements among vertebrates, protein-coding and tRNA genes are switched and shuffled among duplicated origins of replication for the heavy strand (putative Control Regions, O_H_). A third of the mt-genome is significantly rearranged, two protein-coding genes are switched (ND5 and ND6), tRNA genes are drastically reshuffled, and putative origins of replication are duplicated and inserted in this region (Fig. 1). Putative Control Regions are observed in triplicate going through concerted evolution, and an unusual stem-and-loop structure is discovered with overlapping sequences of *tRNA*^*Asn*^ and *tRNA*^*Cys*^ in the location of light-strand replication (O_L_). These observations in totality reinforce the idea of replication errors driving mt-genomic rearrangement^6^.

Tandem complementary efforts of high-throughput short-read sequencing with newly advanced long-read sequencing identify two deeply divergent mt-genomes escaping science for decades. The genomic structure is elucidated via these techniques with a single Oxford Nanopore read covering both DNA strands, signaling a new era of organellar genomics with whole molecule sequencing.

## Methods

### Sampling

The LAI sample is a male and NCBI Biosample SAMN08793959 with the four Stephens Island samples all being female: (SI-1) SAMN10598677, (SI-2) SAMN10598679, (SI-3) SAMN10598680, and (SI-4) SAMN00855319 with published transcriptome data in SRA051647^18^. Blood of LAI was collected with ethical permissions from Victoria University of Wellington and under permits supplied by the Department of Conservation, New Zealand.

### Sanger sequencing of Stephens Island samples (SI-1–3)

Shotgun Sanger sequencing of SI-1 PCR fragments from *COIII* to *12 S rRNA* follows methods previously described^30^, with amplifications conducted using L9940 5’-GCAGCATGATACTGACACTTYGT-3’ and H1067^6^. A MegaBACE 1000 (Amersham) DNA sequencer ran 768-reads that were assembled using Phrap. Sanger sequencing of SI-2 and SI-3 used two forward REX26_ND5F1 5’-GTGCACTAACACAAAACGATATC-3’ and REX27_ND5F1 5’-GCGCACTGACACAAAATGATATT-3’, and two reverse primers REX26_ND5R1 5’-GGATTCCTCCTATTTTTCGAATG-3’ and REX27_ND5R1 5’- GGATTCCTCCTATTTTTCAGATA-3’ designed in *ND5* from SI-1 sequences. Amplifications applied forward *ND5* primers with H1067^6^ and reverse *ND5* primers with L9940. End-sequencing was done on all PCR fragments with internal reactions using “ND4” ^31^ in *ND4* and “IguaCytBR2”^32^ in *Cytb*. Reactions were run on an ABI3730 Sequencer (2011 Life Technologies) with 900 chemistry (Supplementary Method 1).

### Illumina data collection of the LAI sample

Total genomic DNA was extracted using proteinase K digestion and Phenol-Chloroform extraction from blood. Sequencing was undertaken using the Illumina HiSeq 2000 and 2500 as well as MiSeq sequencing platforms (Illumina, San Diego). Sequencing libraries consisted of paired-end (PE) libraries with estimated insert sizes of 180 bp, 350 bp, and 550 bp and three mate-paired (MP) libraries with estimated insert sizes of 2500 bp, 5000 bp, and 8000 bp. The PE libraries were prepared using the Illumina TruSeq PCR-Free DNA library kit, while the mate-pair libraries were prepared using the Illumina TruSeq DNA library kit as per the manufacturer’s instructions. These libraries were normalized and pooled across 32 lanes on an Illumina HiSeq 2000 or 2500 using 2 × 100 bp PE sequencing at New Zealand Genomics Ltd., Dunedin. We further supplemented these data with additional Illumina TruSeq and Kappa DNA libraries, with insert sizes of 400 bp and 480 bp, respectively. These libraries were normalized and pooled across five Illumina MiSeq 2 × 250 bp runs via New Zealand Genomics Ltd., Dunedin (Supplementary Method 2).

### Illumina assembly of LAI

Illumina reads were mined from total genomic shotgun data for mtDNA reads using the first previously published mt-genome^8^ (AF53439) and initial contigs from two PCR fragments obtained from Sanger sequencing of SI-1 with *ND5, tRNA*^*His*^, and *tRNA*^*Thr*^ herein reported. Illumina HiSeq reads were extracted from eleven PE 100 bp data-sets with an insert size of 180 bp (totaling ~1.9 billion read-pairs) and two additional data-sets with an insert size of 2500 bp (totaling ~47 million read-pairs), and one data-set of 5000 bp (~128 million read-pairs), respectively, using Bowtie 2^33^. Extracted reads were cleaned and only properly paired read-pairs were kept for further analysis. In total, 156,012 reads were obtained that could be used for the assembly of mtDNA. First, the insert size for all mapped reads was calculated and only reads where the determined insert size ranged between 100–200 bp (for the 180 bp library) and 2000–3000 bp (for the 2500 bp library) were used to perform the assembly. The initial assembly was created using MaSuRCA assembler^34^. Next, all contigs mapping to the two PCR fragments obtained from Sanger sequencing of SI-1 reported here were used to fine-tune the Illumina assembly using Minimus^35^. This draft assembly of Illumina HiSeq reads had an average coverage of 739.2 reads (std dev = 200.7; min = 14, max = 1625). In order to evaluate the Minimus assembly, 71mers were counted with one PE 180 bp data-set using Jellyfish^36^ that allowed the identification of a repeat region missed by the initial assembly. After including the repeat into the mtDNA sequence, we obtained a final sequence containing 18,078 bp. The final assembly had an average coverage of 723.2 reads (std dev = 178.3; min = 12, max = 1502). Further examination of Illumina data suggested there may be a second copy of the mt-genome. Initially, all reads mentioned above were de novo assembled in Geneious v10.2.4 (Biomatters, Auckland) with the highest sensitivity producing 937 contigs. Recovered contigs were backmapped to the initial Illumina LAI M1 assembly. Three of the 937 contigs nearly covered the entire molecule (M1) with ~98% identity. Twenty-seven additional contigs that mapped were within 90% identity to the initial LAI M1 mt-genome. These contigs were manually assembled to construct a nearly complete second mt-genome (M2). Therefore, all available Illumina reads were re-mined for mtDNA from Illumina HiSeq PE 100 bp reads from (a) eleven data-sets with an insert size of 180 bp (totaling ~1.9 billion read-pairs), (b) eight data-sets with an insert size of 350 bp (totaling ~446 million read-pairs), (c) eight data-sets with an insert size of 550 bp (totaling ~316 million read-pairs), (d) one data-set with an insert size of 2500 bp (~47 million read-pairs), (e) three data-sets with an insert size of 5000 bp (totaling ~307 million read-pairs), (f) four data-sets with an insert size of 8000 bp (totaling ~490 million read-pairs); and Illumina MiSeq PE 250 bp reads from (g) four data-sets with an insert size of 400 bp (totaling ~50 million read-pairs), and (h) one data-set with an insert size of 480 bp (~17 million read-pairs). This produced 104,825 paired-read sets with a total of 209,650 reads matching mtDNA. The 209,650 reads recovered were de novo assembled in Geneious with the highest sensitivity level, using all paired-read information that produced 856 contigs. Consensus sequences from these contigs were then backmapped to the 18,078 bp draft LAI M1 mt-genome obtained from the above assembly and to the draft M2 in Geneious. Contigs were screened for a minimum of 5% sequence divergence to either the 18,078 bp draft LAI M1 or the draft M2. This produced 19 contigs assigned to M2 for further evaluation. The 19 contigs containing raw reads were separately assembled producing 5 contigs that were manually assembled following manual trimming. These final contigs were backmapped to the draft M2, forming a contig containing 30,005 reads with an average coverage of 169.2 reads (std dev = 102.7; min = 3, max = 508). Ambiguities in M2 were resolved and a final second mt-genome was identified and annotated. Of the 856 contigs discovered above not assigned to M2, nine contigs were deemed unusable. The remaining 828 contigs were backmapped to LAI M1 producing a contig with 176,980 reads having an average coverage of 1007.5 reads (std dev = 213.8; min = 25, max = 1791).

### DNA preparation for Oxford Nanopore sequencing of LAI

Oxford Nanopore work relied on the sample being subsequently shipped between the University of Otago, New Zealand and San Diego Zoo, California, USA using CITES institutional transfers, together with supporting permits to export and import from the New Zealand Department of Conservation and US Fish and Wildlife Service, respectively. A partial sample was shipped to Dovetail (Santa Cruz, CA) for work on the Tuatara genome. Dovetail extracted DNA from ~100 µl of snap-frozen blood using the Qiagen Blood & Cell Culture DNA Midi Kit (Hilden, Germany), yielding 242 ng/µl provided in TE buffer (runs 1–6). A partial blood sample was shipped to SeqMatic LLC (Fremont, CA) for work on the Tuatara genome. SeqMatic (Fremont, CA) extracted DNA from ~50 µl of snap-frozen blood using an enzymatic DNA extraction with (run 7) and without phenol-chloroform (run 8), yielding 131 ng/µl and 79 ng/µl in TE buffer. Following extractions, DNA was stored and transferred to the Peralta Genomics Laboratory (Alameda, CA) at 4**°**C and never frozen (Supplementary Method 3).

### Oxford Nanopore sequencing of LAI

Eight Oxford Nanopore runs using R9.4 chemistry were conducted applying Minion protocol version GDE_9002_v108_revT_18Oct2016 using a nick-repair enzyme (NEBNext FFPE Repair Mix). To increase library yield, NEBNext Ultra II End Repair/dA-Tailing Module was used to eliminate an additional Solid Phase Reversible Immobilization (SPRI)^37^ cleanup step with Agencourt AMPure XP beads. To increase library yield, 2 µl of concentrated NEB T4 DNA ligase was added during adaptor ligation. The general protocol consists of four steps. *Step 1, DNA Nick-Repair, Blunt-Ending, and End Repair*: Add in a clean low bind PCR tube, (a) ~ 2 µg genomic Tuatara DNA as described above of 8.27 µl, (b) NEBNext FFPE Repair Mix of 3 µl, (c) NEBNext Ultra II End Repair/dA-Tailing Mix of 3 µl, (d) NEBNext Ultra II End Repair/dA-Tailing Buffer of 7 µl, (e) 100X NAD + of 0.6 µl, and (f) 10 mM Tris HCl pH 8.5 buffer of 38.13 µl for a total volume of 60 µl. This is mixed gently via inversion and incubated at 20 °C for 60 min and 65 °C for 30 min in a thermocycler. *Step 2, SPRI Cleanup*: add to the above (a) Agencourt AMPure XP beads of 60 µl and let stand for 2 min followed by discarding supernatant while on the magnet, (b) wash with 70% EtOH of 140 µl X 2, and (c) elute in Nuclease-free water of 31 µl. *Step 3, Adaptor Ligation*: add to (a) eluted end-prepped DNA above of 30 µl, (b) 1D Adapter Mix of 20 µl, and (c) NEB Instant Sticky End Ligase of 50 µl, for a total volume of 100 µl. This is mixed gently via inversion and incubated at 20 °C for 10 min in a thermocycler. *Step 4, Library Purification, with additional SPRI Cleanup*: add to the above (a) Agencourt AMPure XP beads of 40 µl and let stand for 2 min followed by discarding supernatant while on the magnet, (b) wash with Adaptor Bead Binding Buffer of 140 µl X 2, and (c) elute in Oxford Nanopore Elution Buffer of 25 µl for DNA sequencing. Deviations to the above protocol are described. *Deviations I, DNA Integrity and Sizing*: DNA was kept in high integrity for runs 3, 5, 6, 7, and 8 but DNA shearing was conducted in two ways for runs 1, 2, and 4. Run number 4 sheared DNA to a target of 5 kb from ~ 2 µg of Tuatara DNA using a Covaris LE220R with (a) Peak Incident Power (W) 100, (b) Duty Factor 20%, (c) Cycles per Burst 1000, and (d) Treatment Time (s) 600. Runs 1 and 2 sheared DNA to a target of 10 kb from ~ 2 µg of Tuatara DNA using a Covaris g-tube. It is noteworthy that while subjecting DNA to shearing a direct following step implementing nick-repair was applied as described above. Final products from shearing procedures required volume adjustments between input DNA (from shearing in buffer) and 10 mM Tris HCl pH 8.5 buffer in the starting protocol of step 1 above to keep relative concentrations equivalent. *Deviations II, Enzymatic Cleanup with Additional SPRI Cleanup*: For runs 2, 5, 6, 7, and 8, replacements steps from steps above apply an activated immobilized trypsin resin (ThermoFisher Scientific). *Step 2.1*, to activate trypsin resin, the resin was gently mixed adding 20 µl of resin to 100 µl of 10 mM Tris HCl pH 8.5 buffer in a 1.5 ml tube. This was inverted for mixing followed by a 6000 rpm spin, with the supernatant discarded; this was repeated two additional times, with 20 µl of 10 mM Tris HCl pH 8.5 buffer added to the end product. *Step 2.2*, the (a) end-repaired DNA from step 1 of 60 µl, and (b) activated trypsin resin of 20 µl from step 2.1 were (c) incubated at 37 °C for 30 min. Only 60 µl of the total volume of 80 µl was retained leaving 20 µl of pelleted resin behind. *Step 3*, add to (a) eluted end-prepped DNA above of 60 µl, (b) 1D Adapter Mix of 20 µl, and (c) NEB Instant Sticky End Ligase of 80 µl, for a total volume of 160 µl. This is mixed gently via inversion and incubated at 20 °C for 10 min in a thermocycler. *Step 4*, add to the above (a) Agencourt AMPure XP beads of 64 µl and let stand for 2 min followed by discarding supernatant while on the magnet, (b) wash with Adaptor Bead Binding Buffer of 140 µl X 2, and (c) elute in Oxford Nanopore Elution Buffer of 25 µl for DNA sequencing. *Deviations III, 2D Ligation and Library Preparation*: For run 3, 2D chemistry requires replacement steps. *Step 3*, add to (a) eluted end-prepped DNA above of 30 µl, (b) 2D Adapter Mix of 10 µl, (c) dH2O of 5 µl, (d) HP Adapter of 2 µl with (d) NEB Instant Sticky End Ligase of 50 µl and (e) incubated for 10 min at room temp; followed with (f) HP tether of 1 µl and (g) 2 µl of concentrated NEB T4 DNA ligase added with (h) additional incubation at room temp for 10 min. The 2 µl of concentrated NEB T4 DNA ligase was added during adaptor ligation with the HP Tether to increase library yield. The total yield is 100 µl for this step. *Step 4.1, MyOne C1 Streptavidin bead preparation*. Add in a 1.5 ml Eppendorf DNA LoBind tube (a) MyOne C1 Streptavidin beads (Invitrogen) of 50 µl and (b) pellet beads on the magnet for 2 min; (c) discard supernatant and (d) add Oxford Nanopore Bead Binding Buffer of 140 µl; (e) vortex until homogeneous and (f) pellet on a magnet for 2 min; (g) discard supernatant and (h) repeat Oxford Nanopore Bead Binding Buffer wash step of 140 µl with pelleting on a magnet for 2 min X 2; (i) add Bead Binding Buffer of 100 µl and (j) label tube as “Washed Beads” for binding step. *Step 4.2*, for binding add (a) Washed Beads from previous step 4.1 of 100 µl to the tube containing the Ligated DNA in step 3 of 100 µl and (b) incubate at room temperature for 5 min; for elution add (c) Oxford Nanopore elution buffer to DNA-bound beads of 25 µl, (d) incubate tube on the hot block at 37 °C for 10 min, (e) pellet beads on the magnet for 2 min, and (f) transfer supernatant containing library into a clean 1.5 ml Eppendorf DNA LoBind tube for DNA sequencing. Libraries were run on SQK-108, SQK-208, and SQK-LSK-109 flow-cells.

### Oxford Nanopore data and evaluation of LAI

Oxford Nanopore 2D reads were extracted using Nanopolish^38^. By default, Nanopolish extracts reads for export either as a 2D consensus read or a 1D template read; hence there is only a single read per double-stranded (2D) or single-stranded (1D) read. Guppy (Oxford Nanopore) was only used to process 1D reads. All Oxford Nanopore reads obtained from genomic data were compiled into a single database and the Tuatara mt-genomic data were blasted against this database using the default parameters for Blastn and Megablast. This was done with both LAI M1 and M2 Illumina data assemblies reported here in separate searches, as the Rest et al^8^. mt-genome was discovered to lack a segment of the mt-genome. Following this 1.5 kb, 3 kb, and 6 kb random seeds were applied from the LAI M1 and M2 Illumina data assemblies reported here in separate searches to further maximize mitochondrial Oxford Nanopore read recovery. Reads less than 500 bp in length were no longer considered. To evaluate if recovered mt-genomic Oxford Nanopore reads were M1 or M2, matches were made to both the LAI Illumina assembly of M1 or the LAI Illumina assembly of M2, separately. This was done in three separate iterations per molecule using NCBI (a) Blastn, (b) Megablast, and (c) Discontiguous Megablast. A cut-off of 5% difference between molecules was adopted because the two Illumina draft molecules are ~10% sequence divergence and Oxford Nanopore reads have a bias of including numerous gaps representing unread bases. Oxford Nanopore sequences assigned to M1 or M2 were separately backmapped to their respective Illumina draft mt-genome molecules using Geneious with default parameters to evaluate depth coverage.

### PacBio sequencing and assembly of Stephen Island sample 3 (SI-3)

Genomic DNA was extracted using the Qiagen Genomic Tip DNA extraction kit from ~20 mg of liver tissue. A PacBio SMRTbell library was prepared using the SMRTbell Express Template Preparation Kit (Pacific Biosciences). A 12 kb and above size selection with the Sage Science BluePippin system was implemented. Prepared libraries were run on the PacBio Sequel platform using version 2.1 chemistry. Two Single-Molecule Real-Time (SMRT) Cells were sequenced, where each library was sequenced on one SMRT cell with 360 min movie lengths. PacBio raw reads were assembled in CANU version 1.8 (Supplementary Method 4).

### Transcriptome library mining

The published Tuatara transcriptome library (SI-4)^18^ was downloaded. Initial work discovered contaminated *Rattus leucopus* DNA sequences. The mt-genome of *R. leucopus*^39^ was used to purge *Rattus* mt-DNA from the Tuatara transcriptome library by using the ‘Map to Reference’ function in Geneious and saving a list of unused reads. The *Rattus*-free mt-DNA reads were mapped to LAI M1 producing a full-coverage of the mt-genome.

### Mt-genome alignment for sequence divergence

Complete mt-genome alignments for pair-wise sequence divergence were conducted in Geneious using MUSCLE version 3.8.425, with adjustments to comply with secondary structures of encoding tRNA genes^40^. The end of the third non-coding block was not alignable and excluded (last 404 bp of NC3 in LAI M2, positions 17912–18315; last 230 bp of NC3 in LAI M1, positions 17849–18078; last 230 bp of NC3 in SI-3, positions 17849–18078; last 230 bp of NC3 in SI-4, positions 17842–18071).

### Phylogenetic analysis of duplicate mt-genomes and duplicate non-coding blocks

Phylogenetic sampling used LAI M1 and M2 with samples from eight northern islands (Plate, Cuvier, Green Mercury, Middle Mercury, Stanley, Red Mercury, Hen, and Poor Knight) and two southern islands (Stephens and North Brother); GenBank numbers KP996609–19, 40–41^9,10^ (Supplementary Method 5). The LAI M2 is 7 encoded amino acid positions longer in *Cytb* than sequences deemed molecule 1. Gaps were placed in all molecule 1 *Cytb* sequences: (a) after encoded amino acid position 3 (nucleotide position 9) 5 encoded amino acid positions were entered as gaps (15 nucleotide positions), and (b) after encoded amino acid position 291 (nucleotide position 873), and 2 encoded amino acid positions were entered as gaps (6 nucleotide positions). *ND5* was not included because it was not reported in all previous studies^8–10^. Overlapping regions of bicistronic-encoded genes were excluded: *Atp8* and *Atp6* (last 96 nucleotides of *Atp8*), and *ND4L* and *ND4* (last 7 nucleotides of *ND4L*). To evaluate duplicated non-coding block sequences (NC1–3) from both LAI mt-genomes, they were compared to sequences from SI-4 of the published transcriptome library (Supplementary Method 6). The SI-3 sample was not included because of ambiguities in sequence. Sequences were aligned in Geneious using MUSCLE version 3.8.425. Only areas in common between NC1–3 sequences were evaluated. Phylogenetic estimates were conducted using branch-and-bound searches in PAUP* version 4.0^41^ with bootstraps generated via 1000 replicates. Decay indices were generated with successive searches retaining suboptimal trees, and computing tree length difference to the overall shortest tree(s). Two-tailed Wilcoxon signed-ranks tests^42,43^ incorporating a correction for tied ranks were used to examine the statistical significance of alternative phylogenetic hypotheses with the shortest estimate(s). Alternative phylogenetic estimates were discovered via searches applying constraint trees that saved suboptimal trees compatible with an alternative phylogenetic hypothesis, using MacClade version 4.03^44^ for constraint tree construction and search implementation in PAUP* version 4.0^41^.

### Protein evaluation

Aligned protein-coding regions of LAI M1 & M2, SI-3, SI-4, and GenBank KP996609–19, 40–41^9,10^ were subjected to PAML-CodeML^45^ (Supplementary Table 1) using ETE3^46^ and to FEL^47^ using HyPhy^48^ to detect selective pressures on individual sites of the LAI M2 lineage. For ND5, the only available LAI M1 & M2, SI-3, and SI-4 sequences were used. Within PAML, we used Bayes Empirical Bayes (BEB)^49^ with a threshold of P > 95% as evidence for sites under putative positive selection. Within FEL, the resulting p values were adjusted for multiple testing using FDR (false discovery rate^50^) across sites. For protein structure visualization, ConSurf Server^51,52^ uses HMMER to search for homologs in the UNIREF-90 database (E-value cut-off of 0.0001), and 150 sampled protein sequences (min/max identity of 35%/95%) are aligned using MAFFT-L-INS-i. HHPred and MODELER then apply the best evolutionary substitution model (JTT) to predict the 3D structure of proteins^52^. The structures are visualized using PyMOL^53^ (see Supplementary Method 7 and Supplementary Tables 1–3).

### Statistics and reproducibility

Phylogenetic statistical analyses are described in Methods. Mt-genome coverage statistics (e.g., average, min/max, depth) were calculated in Geneious. Protein evaluation statistical analyses used Python (http://www.python.org), applying multiple tests library for multiple testing correction. ETE3^46^ was used to perform PAML-CodeML^45^ selection analyses, and HyPhy^48^ to perform FEL^47^ selection analyses. Additional details of all experiments are presented in Supplementary Information.

### Reporting summary

Further information on research design is available in the Nature Research Reporting Summary linked to this article.

## Supplementary information

Supplementary Information

Description of Additional Supplementary Files

Supplementary Data 1

Supplementary Data 2

Reporting Summary

## Data Availability

New DNA sequences generated in this study are deposited as GenBank MN864228– MN864230 and Sequence Read Archive (SRA) PRJNA445603. Published transcriptome sequences^18^ used in this study are from SRA SRA051647 and the complete mt-genome from that transcriptome is deposited as a Third Party Annotation in the DDBJ/ENA/GenBank databases as TPA: BK012001.
