# Supplementary material for: Evidence of two deeply divergent co-existing mitochondrial genomes in the Tuatara reveals an extremely complex genomic organization
Source: Commun Biol. 2021 Jan 29;4:116. doi: 10.1038/s42003-020-01639-0 (PMC7846811; doi:10.1038/s42003-020-01639-0)
Supplement: Supplementary file 1 — Supplementary Information [file 42003_2020_1639_MOESM1_ESM.pdf]

Supplementary Materials

J. Robert Macey, Stephan Pabinger, Charles G. Barbieri, Ella S. Buring, Vanessa L. Gonzalez, Daniel G. Mulcahy, Dustin P. DeMeo, Lara Urban, Paul M. Hime, Stefan Prost, Aaron N. Elliott and Neil J. Gemmell. Evidence of two deeply divergent co-existing mitochondrial genomes in the Tuatara reveals an extremely complex genomic organization. Comms. Bio.

**Content**	**Pages**	**Topic**
Supplementary Note 1	2	Illumina LAI base composition
Supplementary Note 2	2	Oxford Nanopore LAI sequencing
Supplementary Note 3	3	Complete nanopore mt-genome read
Supplementary Note 4	3	Mt-genome sequence divergence
Supplementary Note 5	4	PacBio, transcriptome, PCR-Sanger
Supplementary Note 6	5	Duplicated putative Control Regions
Supplementary Method 1	6–8	SRA Sanger sequencing protocols
Supplementary Method 2	9–16	SRA Illumina sequencing protocols
Supplementary Method 3	17–31	SRA nanopore sequencing protocols
Supplementary Method 4	32–33	SRA PacBio sequencing protocols
Supplementary Method 5	34	Duplicate molecule evaluation
Supplementary Method 6	34	Duplicate Control Region evaluation
Supplementary Method 7	35	Protein structure evaluation
Supplementary Table 1	36	PAML positive selection detection
Supplementary Table 2	37	p-values across genes by LRT
Supplementary Table 3	38	Positive selection codon sites
Supplementary Figure 1 Legend	39	Alignment of nanopore mt-2D read
Supplementary Figure 1	40–62	Alignment of nanopore mt-2D read

Supplementary Note 1

Base composition of Illumina assemlies of Lady Alice Island mt-genomes (M1 and M2). The Illumina assembly of M1 is 18,078 bases in length showing strong strand-bias against Guanine (G) on the light strand with 13.8% G, 33.1% A, 27.3% T, and 25.8 % C, which is typical among vertebrates[6,30]. The Illumina assembly of M2 is 18,315 bases in length also showing strong strand-bias against Guanine (G) on the light strand with 14.6% G, 31.9% A, 26.6% T, and 26.6 % C. It is important to note that base compositions among molecules are quite similar with G ranging between 13.8% and 14.6% as expected for functioning mt-genomes.

Supplementary Note 2

Oxford Nanopore sequencing of the Lady Alice Island sample (LAI). Oxford Nanopore sequencing of LAI produces 9.46 Gb of sequence data in 7,229.48 K reads. The eight runs range from 0.11 Gb to 3.45 Gb yield. Runs 1 and 3–4 applying SPRI cleanup produce 1.56 Gb (218.86 K reads), whereas runs 2 and 5–8 using immobilized trypsin resin as a cleanup produce 7.90 Gb (7,010.63 K reads). In total 342 reads are deemed mitochondrial. Mitochondrial DNA is recovered from all runs except run 4 applying SPRI cleanup. The number of screened mtDNA reads with a 5% difference in match between Illumina drafts of M1 and M2 is 187. Only 158 of screened reads are above 500 bp and used in further analyses. Runs 1 and 3 using SPRI cleanup produce 17 reads with 53.13 kb of data, whereas runs 2 and 5–8 using immobilized trypsin resin as a cleanup produce 141 reads with 332.01 kb of data. Of the 158 screened mtDNA reads, 136 mtDNA reads are assigned to M1, having at least a 5% sequence identity or better match than with M2. Of these 136 reads, 123 are over 500 bp in length. Nine of the 123 reads failed to map producing a total of 114 mapped reads for M1. Reads that mapped to LAI M1 average 2504.5 bp in length (std dev = 2409.2; min = 514; max = 16,978), obtaining the entire M1 with the maximum read length. The entire Illumina reference sequence is covered (100% of 18,078 bp) with an average depth coverage of 16.9 (std dev = 3.4; min = 11; max = 25). Fifty-one mtDNA reads are assigned to M2, having at least a 5% sequence identity or better match than with M1. Of these 51 reads, 35 are over 500 bp in length. Three of the 35 reads failed to map producing a total of 32 mapped reads for molecule 2. Reads that mapped to the LAI M2 mt-genome averaged 1990.2 bp in length (std dev = 1757.6; min = 551; max = 7026). This molecule has 95.8% coverage (17,546 bp of 18,315 bp reference) of the Illumina reference second molecule.

Supplementary Note 3

Oxford Nanopore complete mt-genome 2D read (see Supplementary Method 3 below). The LAI Oxford Nanopore 2D read is identified as M1, and nicked in *ND1* with both strands covering the molecule end to end; providing confidence in this version of the Tuatara mt-genome. The template strand is sequenced as heavy strand mtDNA of 16,978 bases, and the compliment strand is sequenced as light strand mtDNA of 14,211 bases. We report the consensus of these two connected strands during sequencing. The consensus read is 16,978 bases in length, which includes overhangs that were end-trimmed (13 bases on the 5'-end and 17 bases on the 3'-end as light strand sequence), yielding a 16,948 base read. This trimmed read shows strong strand-bias against Guanine (G) on the light strand with 13.6% G, 33.6% A, 27.8% T, and 25.0 % C, which is typical among vertebrates[6,29]. The read is considerably shorter than the LAI Illumina assembly of M1 due to Oxford Nanopore induced gaps when skipping bases during nanochip reading. The alignment of the Oxford Nanopore 2D read covering the entire molecule with the LAI Illumina assembly of M1 has the following differences: (A) The Oxford Nanopore 2D read has 1192 gaps, which are distributed as one 18-base gap, one 11-base gap, three 9-base gaps, three 8-base gaps, two 7-base gaps, eleven 6-base gaps, eight 5-base gaps, twenty-six 4-base gaps, fifty-seven 3-base gaps, one hundred one 2-base gaps, and four hundred thirty-five 1-base gaps, for a total of 688 gap segment events. This does not include two positions in *ND1* missing after trimming of the Oxford Nanopore 2D read, which are either gaps or ambiguous bases in that read but are two G positions in the Illumina assembly. (B) The Illumina assembly has 60 gaps representing imposed extra bases in the Oxford Nanopore read, which are distributed as one 5-base gap, two 3-base gaps, nine 2-base gaps, and thirty-one 1-base gap, for a total of 43 gap segment events. (C) There are 191 transitional differences between the Oxford Nanopore read and the Illumina assembly, which are distributed as 49 R positions and 142 Y positions. (D) There are 41 transversional differences between the Oxford Nanopore read and the Illumina assembly, which are distributed as 7 K positions, 20 W positions, 2 S positions, and 12 M positions. In total there are 1484 differences producing a 92% identity between the Oxford Nanopore 2D read covering the entire molecule and the LAI Illumina assembly of M1.

Supplementary Note 4

Sequence divergence of newly reported mt-genomes. Pair-wise sequence divergence across all alignable sites in the four reported complete mt-genomes (end of NC3 excluded) is most prominent between the LAI M2 genome and the other three genomes deemed molecule 1 (LAI M1 at 10.4%, SI-3 at 10.5% and SI-4 at 10.6%). Across the Cook Strait between the North Island and South Island, the LAI M1 located in the north is 0.8% and 1.1% sequence divergent to SI-3 and SI-4, respectively; the latter two Stephens Island samples are 0.05% divergent in sequence.

Supplementary Note 5

PacBio sequencing of the Stephens Island sample 3 (SI-3). PacBio results of SI-3 produced 251 reads that mapped to the draft Illumina LAI M1 mt-genome with an average coverage of 40.2 reads (std dev = 6.8; min = 5, max = 70). Reads average 4970.6 bp in length (std dev = 3315.4; min = 102; max = 15,151), obtaining nearly the entire molecule with the maximum read length. A consensus sequence for SI-3 produced an mt-genome of 18,078 bp in length. This agrees in structure with the Illumina assembly of LAI M1 mt-genome draft, and shares a pair-wise identity of 99.0%.

Mining the published Tuatara transcriptome library (SI-4). Mapping the SRA library[18] (SI-4) to the LAI M1 mt-genome results in a contig yielding an average coverage of 2841.5 read-depth (std dev = 3359.5; min = 0, gap region; max = 24,559). The contig contains only one gap, in positions 981 through 986, at the very 3' end of the 12S rRNA transcript sequence. Thus, the 21-nucleotide gap at the 3' end of 16S rRNA transcript found when mapping to the Rest et al.[8] mt-genome is completed when mapping to the LAI M1 mt-genome. Therefore, the mt-genome recovered from the transcriptome library (SI-4) matches the genome structure of the LAI M1 and SI-3 mt-genomes herein reported. Hence, the mt-genomic transcript complements raw genomic DNA sequencing of the mt-genome.

Gene order confirmation from Sanger reactions derived from Long PCR amplicons of Stephens Island samples (SI-2, 3). Both samples are confirmed to have the following regional and genic junctions. Positions correspond to the Illumina assembly of LAI M1 mt-genome (GenBank MN864228): (a) positions 9310–10230, *COIII*, $tRNA^{Gly}$, *ND3*, $tRNA^{Arg}$, *ND4L, ND4*; (b) positions 10,790–11,620, *ND4, ND6*; (c) positions 13,352–14,040, *ND5*, $tRNA^{Thr}$; (d) positions 15,400–16,235, NC2, $tRNA^{Leu(CUN)}$ second copy, *Cytb*; and (e) positions 17,820–18,078 plus 1–475, NC3, $tRNA^{Phe}$, *12S rRNA*.

Supplementary Note 6

Duplicated putative Control Regions. Three main noncoding regions are identified that show features consistent with Control Region copies which are expected to function as the replication origin for the heavy strand (O_H). In LAI M1 these noncoding regions are NC1 (positions 12,086–12,909) located between tRNA^{Glu} and tRNA$^{Leu(CUN)}$ copy one, NC2 (positions 14,960–15,782) located between tRNA^{His} and tRNA$^{Leu(CUN)}$ copy two, and NC3 (positions 17,151–18,078) located between tRNA$^{Ser(AGY)}$ and tRNA^{Phe}. NC1 and NC2 are exactly the same except for three minor differences: (a) NC2 is missing the first base of NC1 which is a G (position 12,086), so NC1 is 824 bases in length and NC2 is 823 bases in length; (b) the 11th base of NC1 (position 12,096) is a C instead of a T as in NC2 (position 14,969); (c) the 573rd base of NC1 is an A (position 12,658) instead of a G as in NC2 (position 15,531). NC3 has five nucleotides (positions 17,151–17,155) in front of the G (position 17,156) present in NC1 but has the same sequence in the two variable sites described above as NC2 (11th base of NC1 is a T in NC3 at position 17,166; 573rd base of NC1 is a G in NC3 at position 17,728). NC3 differs in not having the end 58 bases present in NC1 (positions 12,852–12,909) and NC2 (positions 15,725–15,782). Instead NC3 has 157 bases (positions 17,922–18,078) with no identity to NC1 and NC2. Both NC1 and NC2 are followed by 69-nucleotide identical copies of the tRNA$^{Leu(CUN)}$ gene (copy one positions 12,910–12,978, copy two positions 15,783–15,851). Hence, using the NC1 region of 824 nucleotides as a reference, 823 bases align with NC2 containing only two Y or R variable sites, which are then followed by 69 additional nucleotides present in the two identical tRNA$^{Leu(CUN)}$ gene copies to be an 892 nucleotide duplicated segment. NC3 has an exact copy of 764 nucleotides found in NC2. In LAI M2 mt-genome NC1 (positions 12,116–12,933) and NC2 (positions 14,992–15,809) are largely the same with 818 bp each, but have 18 nucleotide changes (7 Y, 2 R, 2 W, 2 M, and 5 K). Most of these changes occur at the beginning and end of the non-coding blocks. As in molecule 1, NC1 has a G (position 12,116) at the beginning not present in NC2. Six gaps in 4 positions are placed in NC1 relative to NC2, and 5 gaps in 2 positions are placed in NC2 relative to NC1. The proceeding tRNA$^{Leu(CUN)}$ gene copies are identical except for 2 Y positions. NC3 is longer with 1,108 bp in total length. The first eight bases do not match NC1 and NC2 with the ninth base a G (position 17,216) as in the first base of NC1. The following 783 bases (positions 17,217–17,999) align to NC1 and NC2. A total of 23 sites differ from either NC1 or NC2 (4 Y, 7 R, 6 W, 3 S, 2 M and 1 K). Two sites match NC1 but not NC2 and 8 sites match NC2 but not NC1. To align with NC1 and NC2 12 gaps are introduced in NC3 in 6 positions, with 5 additional gaps introduced in NC1 and NC2 in 3 positions.

Supplementary Method 1

Data deposition of raw Sanger sequencing reads in the NCBI Sequence Read Archive (SRA) database as PRJNA445603.

DNA sequence reads from three Tuatara samples are sequenced with Sanger raw reads deposited in the NCBI Sequence Read Archive (SRA) as SRP140116/PRJNA445603. (1) SI-1, St. Louis Zoo originating from Stephens Island, Marlborough Sounds, Marlborough Province, New Zealand, Biosample SAMN10598677 (ISIS 373002), (2) SI-2, St. Louis Zoo originating from Stephens Island, Marlborough Sounds, Marlborough Province, New Zealand, BioSample SAMN10598679 (SLZ 14846, ISIS 379002), and (3) SI-3, St. Louis Zoo originating from Stephens Island, Marlborough Sounds, Marlborough Province, New Zealand, Biosample SAMN10598680 (SLZ 19079, ISIS 103796). The SI-1 sample was Sanger shotgun sequenced on a 384-plate, forward and reverse, providing 768 raw reads and listed in A below. The SI-2 and SI-3 samples were subjected to targeted PCR with Sanger sequencing and covered in B below.

A). Sanger DNA-Seq of *Sphenodon punctatus* Stephens Island ISIS 373002 SI-1, SAMN10598677

SRA Experiment SRX5161986; SRA Run SRR8351024

Genomic DNA was extracted from blood of a Tuatara (*Sphenodon punctatus*) residing at St. Louis Zoo (ISIS 373002) originating from Stephens Island, Marlborough District, New Zealand, using the Qiagen QIAamp tissue kit. Amplification of the mtDNA was conducted using rTth long PCR enzyme (Applied Biosystems) with a beginning denaturation at 94C for 45s, then followed by 37 cycles of a denaturation at 94 C for 15 s, annealing at 50 C for 20 s, and extension at 68 C for 9 min, with a final extension at 72 C for 12 min after the last cycle. Negative controls were run on all amplifications to check for contamination. A single primer-pair was used, one in the COIII gene (L9940) 5-GCAGCATGATACTGACACTTYGT-3 (this study) and the other in the 12S rRNA gene (H1067) 5-TAGTGGGGTATCTAATCCCAGTTT-3 (Macey et al. 1997, Mol. Biol. Evol. 14, 91–104). Primers are designated by their 3 ends, which correspond to the position in the human mitochondrial genome (Anderson et al. 1981, Nature 290, 457–465) by convention. H and L designate heavy-strand and light-strand primers, respectively; the mixed base position of Y = T or C. Amplification products were sheared randomly into fragments of approximately 1.5kb by repeated passage through a narrow aperture using a HydroShear device (GeneMachines). After end-repair, the sheared DNA was gel purified and ligated into pUC18 vector to construct a library of random fragments, then transformed into bacterial cells. Automated colony pickers introduced single clones into bacterial broth in 384-well for- mat. These plasmid clones were processed robotically through rolling circle amplification (Dean et al. 2001, Genome Res. 11, 1095–1099; Hawkins et al. 2002, Curr. Opin. Biotechnol. 13, 65–67), sequencing reactions, and reaction clean up using SPRI (Solid Phase Reversible Immobilization; Elkin et al. 2002, Biotechniques 32, 1296–1302). Sequences were determined using a MegaBACE 1000 (Amersham) DNA sequencer.

B). Long-amp PCR based Sanger sequencing for Sphenodon mitochondrial genome

B1. SI-2
SRA Experiment SRX7423932; SRA Run SRR10749103

B2. SI-3
SRA Experiment SRX7423931; SRA Run SRR10749102

Genomic DNA was extracted from liver of two female Tuatara previously residing at the St. Louis Zoo [BioSample SAMN10598679 (ISIS379002, SLZ 14846) and BioSample SAMN10598680 (ISIS103796, SLZ 19079)] originating from Stephens Island, Marlborough District, New Zealand, using the Qiagen Genomic Tip DNA extraction kit (genomic-tip column 100/G) from ~20mg of liver tissue according to the manufacturer's directions at the Laboratories of Analytical Biology at the Smithsonian Institutions National Museum of Natural History (NMNH). Two sets of forward and reverse primers were designed on the Stephens Island (BioSample SAMN10598677, ISIS 373002) Sanger sequences generated from Long-amp PCR. Two forward (Rex26_ND5F1) 5-GTGCACTAACACAAAACGATATC-3 and (Rex27_ND5F1) 5-GCGCACTGACACAAAATGATATT-3 and two reverse primers (Rex26_ND5R1) 5-GGATTCCTCCTATTTTTCGAATG-3 and (Rex27_ND5R1) 5-GGATTCCTCCTATTTTTCAGATA-3 were designed in the middle of the ND5 gene, where the two mt-genome copies differed significantly. Primer sets were designed to amplify only one or the other of the differing copies (Rex26Rex27). Forward ND5 primers were used with the 12S rRNA primer (H1067; Macey et al. 1997, Mol. Biol. Evol., 14, 91–104) 5-TAGTGGGGTATCTAATCCCAGTTT-3 and reverse ND5 primers were used with the COIII primer (L9940) 5-GCAGCATGATACTGACACTTYGT-3 (this study) in long-amp reactions, producing fragments ~5 kb long. Polymerase chain reactions (PCR) were conducted on the two separate [BioSample SAMN10598679 (ISIS379002, SLZ 14846) and BioSample SAMN10598680 (ISIS103796, SLZ 19079)] Qiagen Genomic Tip DNA extractions, using LongAmp taq (New England BioLabs Inc. #M0323S), in 25 µl reactions, with 12 µl of template, and amplified with a beginning denaturation at 94 C for 45 s, then followed by 37 cycles of a denaturation at 94 C for 15 s, annealing at 50 C for 20 s, and extension at 68 C for 9 min, with a final extension at 72 C for 12 min after the last cycle. Negative controls were run on all amplifications to check for contamination. PCR products were purified using ExoSapIT and end-sequence reactions were performed with the PCR primers using BigDye Terminator v3.1 Cycle Sequencing Kits in 0.25 10 µl reactions and run on an Automated ABI3730 Sequencer (2011 Life Technologies) with the 900 chemistry. Additionally, internal primers were also used in cycle-sequence reactions on the long-amp (~5 kb) products, including the forward primer (ND4) 5-CACCTATGACTACCAGCTCATGTAGAAGC-3 (Arevelo et al. 1994, Systematic Biology 43, 387–418) and the reverse primer (IguaCytoBR2) 5-GGTTTACAAGACCAATGCTTT-3 (Corl et al., 2010, PNAS 107, 4254–4259). Using Geneious v10.2.4 (Biomatters Ltd 2005–2017), raw trace files from both sample extractions were initially aligned with the Lady Alice first molecule mitochondrial genome (BioSample SAMN10598677, ISIS 373002), and the BioSample

SAMN10598680 (ISIS103796, SLZ 19079) sequences were later aligned with the PacBio-produced mitochondrial genome of the same individual resolving several ambiguities. Trace files are named by the specimen number (SLZ 14846 and SLZ 19079) followed by Spheno_Rex26 or Rex27 (from which fragment ND5f or ND5r primers were amplified and where f designates light-strand extending and r designates heavy-strand extending primers), followed by the primer used for sequencing.

Supplementary Method 2

Data deposition of raw Illumina sequencing reads in the NCBI Sequence Read Archive (SRA) database as PRJNA445603. Illumina DNA sequence reads from Tuatara Passive Integrated Transponder (PIT) 982 000167713537 originating from Lady Alice Island, Hen and Chickens Islands, New Zealand (NCBI Biosample SAMN08793959; this study LAI) are deposited in the NCBI Sequence Read Archive (SRA) as SRP140116/PRJNA445603.

Data are distributed in 80 fastq files of 40 sets accommodating paired reads. Sequencing strategy and sequencing instrument are (1) first described in a title with a note of number of paired-reads, followed by (2) a protocol, (3) the number of paired fastq file sets, and (4) a list of those fastq file sets. Preceeding each of the 40 fastq file sets are SRA experiment numbers in bold and run numbers in plain text as they apprear in PRJNA445603 for reference.

In summary: (A) 2x100 PE 180 bp insert, Illumina HiSeq 2000, 11 sets of 2 fastq files (totaling ~1.9 billion read-pairs); (B) 2x100 PE 350 bp insert, Illumina HiSeq 2500, 6 sets of 2 fastq files; (C) 2x100 PE 350 bp insert, Illumina HiSeq 2000, 2 sets of 2 fastq files (B & C totaling ~446 million read-pairs); (D) 2x250 PE 400 bp insert, Illumina MiSeq, 4 sets of 2 fastq files (totaling ~50 million read-pairs); (E) 2x250 PE 480 bp insert, Illumina MiSeq, 1 set of 2 fastq files (~17 million read-pairs); (F) 2x100 PE 550 bp insert, Illumina HiSeq 2000, 6 sets of 2 fastq files; (G) 2x100 PE 550 bp insert, Illumina HiSeq 2000, 2 sets of 2 fastq files; (F & G totaling ~316 million read-pairs; (H) 2x100 MP 2500 bp insert, Illumina HiSeq 2000, 1 set of 2 fastq files (~47 million read-pairs); (I) 2x100 MP 5000 bp insert, Illumina HiSeq 2000, 1 set fastq files; (J) 2x100 MP 5000 bp insert, Illumina HiSeq 2500, 2 sets of 2 fastq files (I & J totaling ~490 million read-pairs); (K) 2x100 MP 8000 bp insert, Illumina HiSeq 2000, 2 sets of 2 fastq files; and (L) 2x100 MP 8000 bp insert, Illumina HiSeq 2500, 2 sets of 2 fastq files (K & L totaling ~490 million read-pairs).

In this, sequencing libraries consisting of paired end (PE) libraries with estimated insert sizes of 180, 350, 400, 480, and 550 bp and libraries consisting of mate pair (MP) libraries with estimated insert size of 2500, 5000, 8000 bp are run and resulting data deposition files are as follows.

A). 2x100 PE 180 bp insert, Illumina HiSeq 2000

Total genomic DNA was extracted using proteinase K digestion and Phenol-Chloroform extraction from blood. Sequencing was undertaken using the Illumina HiSeq 2000 sequencing platform (Illumina, San Diego, CA, USA). Sequencing libraries consisted of paired end (PE) libraries with estimated insert size of 180 bp. The paired end libraries were prepared using the Illumina TruSeq PCR-Free DNA library kit as per manufacturers instructions. These libraries were normalised and pooled across 11 lanes of Illumina HiSeq 2000 using 2 x 100 bp paired end sequencing at New Zealand Genomics Ltd., Dunedin.

11 Sets

SRA Experiment SRX3937384; SRA Run SRR7004786
ACOTKVACXX_NZGL00054_Tru_2x100_180_L001_R1.fastq.gz
ACOTKVACXX_NZGL00054_Tru_2x100_180_L001_R2.fastq.gz

SRA Experiment SRX3937385; SRA Run SRR7004785
ACOTKVACXX_NZGL00054_Tru_2x100_180_L002_R1.fastq.gz
ACOTKVACXX_NZGL00054_Tru_2x100_180_L002_R2.fastq.gz

SRA Experiment SRX3937386; SRA Run SRR7004784
ACOTKVACXX_NZGL00054_Tru_2x100_180_L003_R1.fastq.gz
ACOTKVACXX_NZGL00054_Tru_2x100_180_L003_R2.fastq.gz

SRA Experiment SRX3937387; SRA Run SRR7004783
ACOTKVACXX_NZGL00054_Tru_2x100_180_L004_R1.fastq.gz
ACOTKVACXX_NZGL00054_Tru_2x100_180_L004_R2.fastq.gz

SRA Experiment SRX3937388 ; SRA Run SRR7004782
ACOTKVACXX_NZGL00054_Tru_2x100_180_L005_R1.fastq.gz
ACOTKVACXX_NZGL00054_Tru_2x100_180_L005_R2.fastq.gz

SRA Experiment SRX3937389; SRA Run SRR7004781
ACOTKVACXX_NZGL00054_Tru_2x100_180_L006_R1.fastq.gz
ACOTKVACXX_NZGL00054_Tru_2x100_180_L006_R2.fastq.gz

SRA Experiment SRX3937390; SRA Run SRR7004780
ACOTKVACXX_NZGL00054_Tru_2x100_180_L007_R1.fastq.gz
ACOTKVACXX_NZGL00054_Tru_2x100_180_L007_R2.fastq.gz

SRA Experiment SRX3937391; SRA Run SRR7004779
ACOTKVACXX_NZGL00054_Tru_2x100_180_L008_R1.fastq.gz
ACOTKVACXX_NZGL00054_Tru_2x100_180_L008_R2.fastq.gz

SRA Experiment SRX3937382; SRA Run SRR7004788
BCOGKKACXX_NZGL00054_Tru_2x100_180_L005_R1.fastq.gz
BCOGKKACXX_NZGL00054_Tru_2x100_180_L005_R2.fastq.gz

SRA Experiment SRX3937383; SRA Run SRR7004787
BCOGKKACXX_NZGL00054_Tru_2x100_180_L006_R1.fastq.gz
BCOGKKACXX_NZGL00054_Tru_2x100_180_L006_R2.fastq.gz

SRA Experiment SRX3937364; SRA Run SRR7004806
BCOGKKACXX_NZGL00054_Tru_2x100_180_L007_R1.fastq.gz
BCOGKKACXX_NZGL00054_Tru_2x100_180_L007_R2.fastq.gz

B). 2x100 PE 350 bp insert, Illumina HiSeq 2500

Total genomic DNA was extracted using proteinase K digestion and Phenol-Chloroform extraction from blood. Sequencing was undertaken using the Illumina HiSeq 2500 sequencing platform (Illumina, San Diego, CA, USA). Sequencing libraries consisted of paired end (PE) libraries with estimated insert size of 350 bp. The paired end libraries were prepared using the Illumina TruSeq PCR-Free DNA library kit as per manufacturers instructions. These libraries were normalised and pooled across 8 lanes of Illumina HiSeq 2000 or 2500 using 2 x 100 bp paired end sequencing at New Zealand Genomics Ltd., Dunedin.

6 Sets

SRA Experiment SRX3937365; SRA Run SRR7004805
H73VYADXX_681_Tru_2x100_350_L001_R1.fastq.gz
H73VYADXX_681_Tru_2x100_350_L001_R2.fastq.gz

SRA Experiment SRX3937362; SRA Run SRR7004808
H76LEADXX_681_Tru_2x100_350_L001_R1.fastq.gz
H76LEADXX_681_Tru_2x100_350_L001_R2.fastq.gz

SRA Experiment SRX3937363; SRA Run SRR7004807
H76LEADXX_681_Tru_2x100_350_L002_R1.fastq.gz
H76LEADXX_681_Tru_2x100_350_L002_R2.fastq.gz

SRA Experiment SRX3937368; SRA Run SRR7004802
H7F83ADXX-681_Tru_2x100_350_L002_R1.fastq.gz
H7F83ADXX-681_Tru_2x100_350_L002_R2.fastq.gz

SRA Experiment SRX3937367; SRA Run SRR7004803
H7GFFADXX_681_Tru_2x100_350_L001_R1.fastq.gz
H7GFFADXX_681_Tru_2x100_350_L001_R2.fastq.gz

SRA Experiment SRX3937370; SRA Run SRR7004800
H7GFFADXX_681_Tru_2x100_350_L002_R1.fastq.gz
H7GFFADXX_681_Tru_2x100_350_L002_R2.fastq.gz

C). 2x100 PE 350 bp insert, Illumina HiSeq 2000

Total genomic DNA was extracted using proteinase K digestion and Phenol-Chloroform extraction from blood. Sequencing was undertaken using the Illumina HiSeq 2000 sequencing platform (Illumina, San Diego, CA, USA). Sequencing libraries consisted of paired end (PE) libraries with estimated insert size of 350 bp. The paired end libraries were prepared using the Illumina TruSeq PCR-Free DNA library kit as per manufacturers instructions. These libraries were normalised and pooled across 8 lanes of Illumina HiSeq 2000 or 2500 using 2 x 100 bp paired end sequencing at New Zealand Genomics Ltd., Dunedin.

2 Sets

SRA Experiment SRX3937369; SRA Run SRR7004801
C2MGWACXX-681_Tru_2x100_350_L002_R1.fastq.gz
C2MGWACXX-681_Tru_2x100_350_L002_R2.fastq.gz

SRA Experiment SRX3937366; SRA Run SRR7004804
C2MGWACXX-681_Tru_2x100_350_L003_R1.fastq.gz
C2MGWACXX-681_Tru_2x100_350_L003_R2.fastq.gz

D). 2x250 PE 400 bp insert, Illumina MiSeq

Total genomic DNA was extracted using proteinase K digestion and Phenol-Chloroform extraction from blood. Sequencing was undertaken using the Illumina MiSeq sequencing platform (Illumina, San Diego, CA, USA). Sequencing libraries consisted of paired end (PE) libraries with estimated insert sizes of 400 bp. The paired end libraries were prepared using the Illumina Kappa DNA library kits as per manufacturers instructions. These libraries were normalised and pooled across 4 lanes of Illumina MiSeq using 2 x 250 bp paired end sequencing at New Zealand Genomics Ltd., Dunedin. fastq

4 Sets

SRA Experiment SRX3937371; SRA Run SRR7004799
121116_M00933_0014_000000000-A1Y07-97_Kappa_2x250_400_L001_R1.fastq.gz
121116_M00933_0014_000000000-A1Y07-97_Kappa_2x250_400_L001_R2.fastq.gz

SRA Experiment SRX3937381; SRA Run SRR7004789
121120_M00933_0015_000000000-A1Y1W-97_Kappa_2x250_400_L001_R1.fastq.gz
121120_M00933_0015_000000000-A1Y1W-97_Kappa_2x250_400_L001_R2.fastq.gz

SRA Experiment SRX3937380 = SRR7004790
121208_M00933_0021_000000000-A1V7W-97_Kappa_2x250_400_L001_R1.fastq.gz
121208_M00933_0021_000000000-A1V7W-97_Kappa_2x250_400_L001_R2.fastq.gz

SRA Experiment SRX937379; SRA Run SRR7004791
121210_M00933_0022_000000000-A1VJG-97_Kappa_2x250_400_L001_R1.fastq.gz
121210_M00933_0022_000000000-A1VJG-97_Kappa_2x250_400_L001_R2.fastq.gz

E). 2x250 PE 480 bp insert, Illumina MiSeq

Total genomic DNA was extracted using proteinase K digestion and Phenol-Chloroform extraction from blood. Sequencing was undertaken using the Illumina MiSeq sequencing platform (Illumina, San Diego, CA, USA). The sequencing library consisted of a paired end (PE) library with estimated insert sizes of 480 bp. The paired end library was prepared using the Illumina TruSeq DNA library kit as per manufacturers instructions. The library was run on one lane of Illumina MiSeq using 2 x 250 bp paired end sequencing at New Zealand Genomics Ltd., Dunedin.

1 Set

SRA Experiment SRX3937378; SRA Run SRR7004792
121130_M00933_0017_000000000-A1VGC-97_Tru_2x250_480_L001_R1.fastq.gz
121130_M00933_0017_000000000-A1VGC-97_Tru_2x250_480_L001_R2.fastq.gz

F). 2x100 PE 550 bp insert, Illumina HiSeq 2500

Total genomic DNA was extracted using proteinase K digestion and Phenol-Chloroform extraction from blood. Sequencing was undertaken using the Illumina HiSeq 2500 sequencing platform (Illumina, San Diego, CA, USA). Sequencing libraries consisted of paired end (PE) libraries with estimated insert size of 550 bp. The paired end libraries were prepared using the Illumina TruSeq PCR-Free DNA library kit as per manufacturers instructions. These libraries were normalised and pooled across 8 lanes of Illumina HiSeq 2000 or 2500 using 2 x 100 bp paired end sequencing at New Zealand Genomics Ltd., Dunedin.

6 Sets

SRA Experiment SRX3937377; SRA Run SRR7004793
H73VYADXX_681_Tru_2x100_550_L001_R1.fastq.gz
H73VYADXX_681_Tru_2x100_550_L001_R2.fastq.gz

SRA Experiment SRX3937376; SRA Run SRR7004794
H76LEADXX_681_Tru_2x100_550_L001_R1.fastq.gz
H76LEADXX_681_Tru_2x100_550_L001_R2.fastq.gz

SRA Experiment SRX3937375 = SRR7004795
H76LEADXX_681_Tru_2x100_550_L002_R1.fastq.gz
H76LEADXX_681_Tru_2x100_550_L002_R2.fastq.gz

SRA Experiment SRX3937374; SRA Run SRR7004796
H7F83ADXX-681_Tru_2x100_550_L002_R1.fastq.gz
H7F83ADXX-681_Tru_2x100_550_L002_R2.fastq.gz

SRA Experiment SRX3937354; SRA Run SRR7004816
H7GFFADXX_681_Tru_2x100_550_L001_R1.fastq.gz
H7GFFADXX_681_Tru_2x100_550_L001_R2.fastq.gz

SRA Experiment SRX3937355; SRA Run SRR7004815
H7GFFADXX_681_Tru_2x100_550_L002_R1.fastq.gz
H7GFFADXX_681_Tru_2x100_550_L002_R2.fastq.gz

G). 2x100 PE 550 bp insert, Illumina HiSeq 2000

Total genomic DNA was extracted using proteinase K digestion and Phenol-Chloroform extraction from blood. Sequencing was undertaken using the Illumina HiSeq 2000 sequencing platform (Illumina, San Diego, CA, USA). Sequencing libraries consisted of paired end (PE) libraries with estimated insert size of 550 bp. The paired end libraries were prepared using the Illumina TruSeq PCR-Free DNA library kit as per manufacturers instructions. These libraries were normalised and pooled across 8 lanes of Illumina HiSeq 2000 or 2500 using 2 x 100 bp paired end sequencing at New Zealand Genomics Ltd., Dunedin.

2 Sets

SRA Experiment SRX3937373; SRA Run SRR7004797
C2MGWACXX-681_Tru_2x100_550_L002_R1.fastq.gz
C2MGWACXX-681_Tru_2x100_550_L002_R2.fastq.gz

SRA Experiment SRX3937372; SRA Run SRR7004798
C2MGWACXX-681_Tru_2x100_550_L003_R1.fastq.gz
C2MGWACXX-681_Tru_2x100_550_L003_R2.fastq.gz

H). 2x100 MP 2500 bp insert, Illumina HiSeq 2000

Total genomic DNA was extracted using proteinase K digestion and Phenol-Chloroform extraction from blood. Sequencing was undertaken using the Illumina HiSeq 2000 sequencing platform (Illumina, San Diego, CA, USA). The sequencing library consisted of a mate paired (MP) library with estimated insert sizes of 2.5 kbp. The mate pair library was prepared using the Illumina TruSeq DNA library kit as per manufacturers instructions. This library was run on 1 lane of Illumina HiSeq 2000 using 2 x 100 bp paired end sequencing at New Zealand Genomics Ltd., Dunedin.

1 Set

SRA Experiment SRX3937356; SRA Run SRR7004814
BDOVECACXX_NZGL00054_Tru_2x100_2500_L008_R1.fastq.gz
BDOVECACXX_NZGL00054_Tru_2x100_2500_L008_R2.fastq.gz

I). 2x100 MP 5000 bp insert, Illumina HiSeq 2000

Total genomic DNA was extracted using proteinase K digestion and Phenol-Chloroform extraction from blood. Sequencing was undertaken using the Illumina HiSeq 2000 sequencing platform (Illumina, San Diego, CA, USA). Sequencing libraries consisted of mate pair (MP) libraries with estimated insert size of 5 kbp. The paired end libraries were prepared using the Illumina TruSeq PCR-Free DNA library kit as per manufacturers instructions. These libraries were normalised and pooled across 3 lanes of Illumina HiSeq 2000 or 2500 using 2 x 100 bp paired end sequencing at New Zealand Genomics Ltd., Dunedin.

1 Set

SRA Experiment SRX3937357; SRA Run SRR7004813
BCOGKKACXX_NZGL00054_Tru_2x100_5000_L008_R1.fastq.gz
BCOGKKACXX_NZGL00054_Tru_2x100_5000_L008_R2.fastq.gz

J). 2x100 MP 5000 bp insert, Illumina HiSeq 2500

Total genomic DNA was extracted using proteinase K digestion and Phenol-Chloroform extraction from blood. Sequencing was undertaken using the Illumina HiSeq 2500 sequencing platform (Illumina, San Diego, CA, USA). Sequencing libraries consisted of mate pair (MP) libraries with estimated insert size of 5 kbp. The paired end libraries were prepared using the Illumina TruSeq PCR-Free DNA library kit as per manufacturers instructions. These libraries were normalised and pooled across 3 lanes of Illumina HiSeq 2000 or 2500 using 2 x 100 bp paired end sequencing at New Zealand Genomics Ltd., Dunedin.

2 Sets

SRA Experiment SRX3937358; SRA Run SRR7004812
H73UVADXX_NZGL00627_Tru_2x100_5000_L001_R1.fastq.gz
H73UVADXX_NZGL00627_Tru_2x100_5000_L001_R2.fastq.gz

SRA Experiment SRX39373359; SRA Run SRR7004811
H73UVADXX_NZGL00627_Tru_2x100_5000_L002_R1.fastq.gz
H73UVADXX_NZGL00627_Tru_2x100_5000_L002_R2.fastq.gz

K). 2x100 MP 8000 bp insert, Illumina HiSeq 2000

Total genomic DNA was extracted using proteinase K digestion and Phenol-Chloroform extraction from blood. Sequencing was undertaken using the Illumina HiSeq 2000 sequencing platform (Illumina, San Diego, CA, USA). Sequencing libraries consisted of mate pair (MP) libraries with estimated insert size of 8 kbp. The paired end libraries were prepared using the Illumina TruSeq PCR-Free DNA library kit as per manufacturers instructions. These libraries were normalised and pooled across 4 lanes of Illumina HiSeq 2000 or 2500 using 2 x 100 bp paired end sequencing at New Zealand Genomics Ltd., Dunedin.

2 Sets

SRA Experiment SRX3937360; SRA Run SRR7004810
C1PF2ACXX_NZGL00166_Tru_2x100_8000_L001_R1.fastq.gz
C1PF2ACXX_NZGL00166_Tru_2x100_8000_L001_R2.fastq.gz

SRA Experiment SRX3937361; SRA Run SRR7004809
C1PF2ACXX_NZGL00166_Tru_2x100_8000_L002_R1.fastq.gz
C1PF2ACXX_NZGL00166_Tru_2x100_8000_L002_R2.fastq.gz

L). 2x100 MP 8000 bp insert, Illumina HiSeq 2500

Total genomic DNA was extracted using proteinase K digestion and Phenol-Chloroform extraction from blood. Sequencing was undertaken using the Illumina HiSeq 2500 sequencing platform (Illumina, San Diego, CA, USA). Sequencing libraries consisted of mate pair (MP) libraries with estimated insert size of 8 kbp. The paired end libraries were prepared using the Illumina TruSeq PCR-Free DNA library kit as per manufacturers instructions. These libraries were normalised and pooled across 4 lanes of Illumina HiSeq 2000 or 2500 using 2 x 100 bp paired end sequencing at New Zealand Genomics Ltd., Dunedin.

2 Sets

SRA Experiment SRX3937352; SRA Run SRR7004818
H73UVADXX_NZGL00627_Tru_2x100_8000_L001_R1.fastq.gz
H73UVADXX_NZGL00627_Tru_2x100_8000_L001_R2.fastq.gz

SRA Experiment SRX3937353; SRA Run SRR7004817
H73UVADXX_NZGL00627_Tru_2x100_8000_L002_R1.fastq.gz
H73UVADXX_NZGL00627_Tru_2x100_8000_L002_R2.fastq.gz

Supplementary Method 3

Data deposition of raw Oxford Nanopore sequencing reads in the NCBI Sequence Read Archive (SRA) database as PRJNA445603. Oxford Nanopore DNA sequence reads from Tuatara Passive Integrated Transponder (PIT) 982 000167713537 originating from Lady Alice Island, Hen and Chickens Islands, New Zealand (NCBI Biosample SAMN08793959; this study LAI) are deposited in the NCBI Sequence Read Archive (SRA) as SRP140116/PRJNA445603.

Fifteen Oxford Nanopore runs were conducted, of which 8 were used in this study as 7 runs were done on DNA extracted by Dovetail (Santa Cruz, CA) that purged mtDNA, and no mtDNA was detected. Runs used are SRA runs 1–3, 5, 6, 10, 14, and 15 (= this study runs 1–8, Table 1 main text), with run 10 having mtDNA but also extracted by Dovetail (Santa Cruz, CA) that purged mtDNA. All runs were searched for mtDNA, and SRA runs 4, 7–9, 11–13 in which mtDNA was purged and not detected are provided to not confuse the reader and share unique protocol information. Please note run numbers used in the main text Table 1 in this study are runs 1–3 which are SRA run numbers 1–3, run number 4 is SRA run number 5, run number 5 is SRA run number 6, run number 6 is SRA run number 10, run number 7 is SRA run number 14 and run number 8 is SRA run number 15.

A). SRA RUN_1 [Run 1 this study]
10kb_Shear_Barcode115_SQK-108

SRA Experiment SRX4909469; SRA Runs SRR8093109, 8082397

Total genomic DNA was extracted from blood using the Qiagen Blood & Cell Culture DNA Midi Kit (Hilden, Germany, cat# 13343), yielding 242 ng/µl provided in TE buffer. Sequencing was undertaken using the Oxford Nanopore MinIon (machine ID No. MN19030; flow-cell version SQK-108) with R9.4 chemistry and conducted using Minion protocol version GDE_9002_v108_revT_18Oct2016 for library preparation with modification. A nick-repair enzyme (NEBNext FFPE Repair Mix) to repair breaks in one side of the DNA double helix, which can prematurely shorten DNA reads was added. To increase library yield, NEBNext Ultra II End Repair/dA-Tailing Module was used to eliminate an additional SPRI (Solid Phase Reversible Immobilization) cleanup step, here applying Agencourt AMPure XP beads. To increase library yield, 2 µl of concentrated NEB T4 DNA ligase was added during adaptor ligation. 10 kb DNA Shearing DNA was sheared to a target of 10 kb from ~ 2 µg of Tuatara DNA using a Covaris g-tube. It is noteworthy that while subjecting DNA to shearing a direct following step implementing nick-repair was applied. Final products from this shearing procedure required volume adjustments between input DNA (from shearing in buffer) and 10 mM Tris HCl pH 8.5 buffer in the starting protocol of step 1 below to keep relative concentrations equivalent.
1. DNA Nick-Repair, Blunt-Ending, and End Repair Add in a clean low bind PCR tube, (a) ~ 2 µg genomic Tuatara DNA as described above of 8.27 µl, (b) NEBNext FFPE Repair Mix of 3 µl, (c) NEBNext Ultra II End Repair/dA-Tailing Mix of 3 µl, (d) NEBNext Ultra II End Repair/dA-Tailing Buffer of 7 µl, (e) 100X NAD+ of 0.6 µl, and

(f) 10 mM Tris HCl pH 8.5 buffer of 38.13 µl for a total volume of 60 µl. This is mixed gently via inversion and incubated at 20°C for 60 min and 65°C for 30 min in a thermocycler. 2. SPRI Cleanup Add to the above (a) Agencourt AMPure XP beads of 60 µl and let stand for 2 min followed by discarding supernatant while on magnet, (b) wash with 70% EtOH of 140 µl X 2, and (c) elute in Nuclease-free water of 31 µl. 3. Adaptor Ligation Add to (a) eluted End-prepped DNA above of 30 µl, (b) 1D Adapter Mix of 20 µl, and (c) NEB Instant Sticky End Ligase of 50 µl, for a total volume of 100 µl. This is mixed gently via inversion and incubated at 20°C for 10 min in a thermocycler. 4. Library Purification, with additional SPRI Cleanup Add to the above (a) Agencourt AMPure XP beads of 40 µl and let stand for 2 min followed by discarding supernatant while on magnet, (b) wash with Adaptor Bead Binding Buffer of 140 µl X 2, and (c) elute in Oxford Nanopore Elution Buffer of 25 µl for DNA sequencing. Library was run on an SQK-108 flow-cell.

B). SRA RUN_2 [Run 2 this study]
10kb_Shear_ Trypsin_Resin_Barcode115_SQK-108

SRA Experiment SRX4909470; SRA Runs SRR8093110, 8082395

Total genomic DNA was extracted from blood using the Qiagen Blood & Cell Culture DNA Midi Kit (Hilden, Germany, cat# 13343), yielding 242 ng/µl provided in TE buffer. Sequencing was undertaken using the Oxford Nanopore MinIon (machine ID No. MN19030; flow-cell version SQK-108) with R9.4 chemistry and conducted using Minion protocol version GDE_9002_v108_revT_18Oct2016 for library preparation with modification. A nick-repair enzyme (NEBNext FFPE Repair Mix) to repair breaks in one side of the DNA double helix, which can prematurely shorten DNA reads was added. To increase library yield, NEBNext Ultra II End Repair/dA-Tailing Module was used to eliminate an additional SPRI (Solid Phase Reversible Immobilization) cleanup step, here applying Agencourt AMPure XP beads. To increase library yield, 2 µl of concentrated NEB T4 DNA ligase was added during adaptor ligation.10 kb DNA Shearing DNA was sheared to a target of 10 kb from ~ 2 µg of Tuatara DNA using a Covaris g-tube. It is noteworthy that while subjecting DNA to shearing a direct following step implementing nick-repair was applied. Final products from this shearing procedure required volume adjustments between input DNA (from shearing in buffer) and 10 mM Tris HCl pH 8.5 buffer in the starting protocol of step 1 below to keep relative concentrations equivalent. 1. DNA Nick-Repair, Blunt-Ending, and End Repair Add in a clean low bind PCR tube, (a) ~ 2 µg genomic Tuatara DNA as described above of 8.27 µl, (b) NEBNext FFPE Repair Mix of 3 µl, (c) NEBNext Ultra II End Repair/dA-Tailing Mix of 3 µl, (d) NEBNext Ultra II End Repair/dA-Tailing Buffer of 7 µl, (e) 100X NAD+ of 0.6 µl, and (f) 10 mM Tris HCl pH 8.5 buffer of 38.13 µl for a total volume of 60 µl. This is mixed gently via inversion and incubated at 20°C for 60 min and 65°C for 30 min in a thermocycler. 2. Immobilized Trypsin-Resin in Place of SPRI Cleanup. For this run a deviated protocol to increase library yield from wash cycles and avoid unnecessary DNA shearing from agitation, applied an enzymatic digest of end-repair enzymes in step 1 above replacing the use of SPRI beads. This was done using an activated trypsin. 2.1. To activate trypsin resin, resin was gently mixed adding 20 µl of resin to 100 µl of 10 mM

Tris HCl pH 8.5 buffer in a 1.5 ml tube. This was inverted for mixing flowed by a 6000 rpm spin, with the supernatant discarded; this was repeated two additional times, with 20 µl of 10 mM Tris HCl pH 8.5 buffer added to the end product. 2.2. The (a) end-repaired DNA from step 1 of 60 µl, and (b) activated trypsin resin of 20 µl from step 2.1 were (c) incubate at 37°C for 30 min. Only 60 µl of the total volume of 80 µl was retained leaving 20 µl of pelleted resin behind. 3. Adaptor Ligation Add to (a) eluted End-prepped DNA above of 60 µl, (b) 1D Adapter Mix of 20 µl, and (c) NEB Instant Sticky End Ligase of 80 µl, for a total volume of 180 µl. This is mixed gently via inversion and incubated at 20°C for 10 min in a thermocycler. 4. Library Purification, with additional SPRI Cleanup Add to the above (a) Agencourt AMPure XP beads of 64 µl and let stand for 2 min followed by discarding supernatant while on magnet, (b) wash with Adaptor Bead Binding Buffer of 140 µl X 2, and (c) elute in Oxford Nanopore Elution Buffer of 25 µl for DNA sequencing. Library was run on an SQK-108 flow-cell.

C). SRA RUN_3 [Run 3 this study]
2D_Standard_Protocol_Barcode115_SQK-208

SRA Experiment SRX4909472; SRA Runs SRR8082396, 8097532

Total genomic DNA was extracted from blood using the Qiagen Blood & Cell Culture DNA Midi Kit (Hilden, Germany, cat# 13343), yielding 242 ng/µl provided in TE buffer. Sequencing was undertaken using the Oxford Nanopore MinIon (machine ID No. MN19030; flow-cell version SQK-208) with R9.4 chemistry and conducted using Minion protocol version GDE_9002_v108_revT_18Oct2016 for library preparation with modification. A nick-repair enzyme (NEBNext FFPE Repair Mix) to repair breaks in one side of the DNA double helix, which can prematurely shorten DNA reads was added. To increase library yield, NEBNext Ultra II End Repair/dA-Tailing Module was used to eliminate an additional SPRI (Solid Phase Reversible Immobilization) cleanup step, here applying Agencourt AMPure XP beads. To increase library yield, 2 µl of concentrated NEB T4 DNA ligase was added during adaptor ligation. 1. DNA Nick-Repair, Blunt-Ending, and End Repair Add in a clean low bind PCR tube, (a) ~ 2 µg genomic Tuatara DNA as described above of 8.27 µl, (b) NEBNext FFPE Repair Mix of 3 µl, (c) NEBNext Ultra II End Repair/dA-Tailing Mix of 3 µl, (d) NEBNext Ultra II End Repair/dA-Tailing Buffer of 7 µl, (e) 100X NAD+ of 0.6 µl, and (f) 10 mM Tris HCl pH 8.5 buffer of 38.13 µl for a total volume of 60 µl. This is mixed gently via inversion and incubated at 20°C for 60 min and 65°C for 30 min in a thermocycler. 2. SPRI Cleanup Add to the above (a) Agencourt AMPure XP beads of 60 µl and let stand for 2 min followed by discarding supernatant while on magnet, (b) wash with 70% EtOH of 140 µl X 2, and (c) elute in Nuclease-free water of 31 µl. 3. 2D Adaptor Ligation Add to (a) eluted End-prepped DNA above of 30 µl, (b) 2D Adapter Mix of 10 µl, (c) dH_2O of 5 µl, (d) HP Adapter of 2 µl with (d) NEB Instant Sticky End Ligase of 50 µl and (e) incubated for 10 min at room temp; followed with (f) HP tether of 1 µl and (g) 2 µl of concentrated NEB T4 DNA ligase added with (h) additional incubation at room temp for 10 min. The 2 µl of concentrated NEB T4 DNA ligase was added during adaptor ligation with the HP Tether to increase library yield. The total yield is 100 µl for this step. 4. Library Purification requires two steps. 4.1. MyOne C1 Streptavidin beads preparation Add in a

1.5 ml Eppendorf DNA LoBind tube (a) MyOne C1 Streptavidin beads of 50 µl and (b) pellet beads on magnet for 2 min; (c) discard supernatant and (d) add Oxford Nanopore Bead Binding Buffer of 140 µl; (e) vortex until homogeneous and (f) pellet on a magnet for 2 min; (g) discard supernatant and (h) repeat Oxford Nanopore Bead Binding Buffer wash step of 140 µl with pelleting on a magnet for 2 min X 2; (i) add Bead Binding Buffer of 100 µl and (j) label tube as Washed Beads for binding step. 4.2. Purification of Library using Streptavidin Beads For binding add (a) Washed Beads from previous step 4.1 of 100 µl to the tube containing the Ligated DNA in step 3 of 100 µl and (b) incubate at room temperature for 5 min; for elution add (c) Oxford Nanopore elution buffer to DNA-bound beads of 25 µl, (d) incubate tube on hot block at 37°C for 10 min, (e) pellet beads on a magnet for 2 min, and (f) transfer supernatant containing library into a clean 1.5 ml Eppendorf DNA LoBind tube for DNA sequencing. Library was run on an SQK-208 flow-cell.

D). SRA RUN_4 [Not used in this study; mtDNA purged] Standard_Protocol_Barcode115_SQK-108

SRA Experiment SRX4909471; SRA Runs SRR8093111, 8082394

Total genomic DNA was extracted from blood using the Qiagen Blood & Cell Culture DNA Midi Kit (Hilden, Germany, cat# 13343), yielding 242 ng/µl provided in TE buffer. Sequencing was undertaken using the Oxford Nanopore MinIon (machine ID No. MN19030; flow-cell version SQK-108) with R9.4 chemistry and conducted using Minion protocol version GDE_9002_v108_revT_18Oct2016 for library preparation with modification. A nick-repair enzyme (NEBNext FFPE Repair Mix) to repair breaks in one side of the DNA double helix, which can prematurely shorten DNA reads was added. To increase library yield, NEBNext Ultra II End Repair/dA-Tailing Module was used to eliminate an additional SPRI (Solid Phase Reversible Immobilization) cleanup step, here applying Agencourt AMPure XP beads. To increase library yield, 2 µl of concentrated NEB T4 DNA ligase was added during adaptor ligation. 1. DNA Nick-Repair, Blunt-Ending, and End Repair Add in a clean low bind PCR tube, (a) ~ 2 µg genomic Tuatara DNA as described above of 8.27 µl, (b) NEBNext FFPE Repair Mix of 3 µl, (c) NEBNext Ultra II End Repair/dA-Tailing Mix of 3 µl, (d) NEBNext Ultra II End Repair/dA-Tailing Buffer of 7 µl, (e) 100X NAD+ of 0.6 µl, and (f) 10 mM Tris HCl pH 8.5 buffer of 38.13 µl for a total volume of 60 µl. This is mixed gently via inversion and incubated at 20°C for 60 min and 65°C for 30 min in a thermocycler. 2. SPRI Cleanup Add to the above (a) Agencourt AMPure XP beads of 60 µl and let stand for 2 min followed by discarding supernatant while on magnet, (b) wash with 70% EtOH of 140 µl X 2, and (c) elute in Nuclease-free water of 31 µl. 3. Adaptor Ligation Add to (a) eluted End-prepped DNA above of 30 µl, (b) 1D Adapter Mix of 20 µl, and (c) NEB Instant Sticky End Ligase of 50 µl, for a total volume of 100 µl. This is mixed gently via inversion and incubated at 20°C for 10 min in a thermocycler. 4. Library Purification, with additional SPRI Cleanup Add to the above (a) Agencourt AMPure XP beads of 40 µl and let stand for 2 min followed by discarding supernatant while on magnet, (b) wash with Adaptor Bead Binding Buffer of 140 µl X 2, and (c) elute in Oxford Nanopore Elution Buffer of 25 µl for DNA sequencing. Library was run on an SQK-108 flow-cell.

E). SRA RUN_5 [Run 4 this study] 5kb_shear_Standard_Protocol_Barcode115_SQK-208

SRA Experiment SRX4909473; SRA Runs SRR8093112, 8082393

Total genomic DNA was extracted from blood using the Qiagen Blood & Cell Culture DNA Midi Kit (Hilden, Germany, cat# 13343), yielding 242 ng/µl provided in TE buffer. Sequencing was undertaken using the Oxford Nanopore MinIon (machine ID No. MN19030; flow-cell version SQK-208) with R9.4 chemistry and conducted using Minion protocol version GDE_9002_v108_revT_18Oct2016 for library preparation with modification. A nick-repair enzyme (NEBNext FFPE Repair Mix) to repair breaks in one side of the DNA double helix, which can prematurely shorten DNA reads was added. To increase library yield, NEBNext Ultra II End Repair/dA-Tailing Module was used to eliminate an additional SPRI (Solid Phase Reversible Immobilization) cleanup step, here applying Agencourt AMPure XP beads. To increase library yield, 2 µl of concentrated NEB T4 DNA ligase was added during adaptor ligation. 5 kb DNA Shearing DNA was sheared to a target of 5 kb from ~ 2 µg of Tuatara DNA using a Covaris LE220R with (a) Peak Incident Power (W) 100, (b) Peak Incident Power (W) 100, (c) Duty Factor 20%, (d) Cycles per Burst 1000, and (e) Treatment Time (s) 600. It is noteworthy that while subjecting DNA to shearing a direct following step implementing nick-repair was applied. Final products from this shearing procedure required volume adjustments between input DNA (from shearing in buffer) and 10 mM Tris HCl pH 8.5 buffer in the starting protocol of step 1 below to keep relative concentrations equivalent. 1. DNA Nick-Repair, Blunt-Ending, and End Repair Add in a clean low bind PCR tube, (a) ~ 2 µg genomic Tuatara DNA as described above of 8.27 µl, (b) NEBNext FFPE Repair Mix of 3 µl, (c) NEBNext Ultra II End Repair/dA-Tailing Mix of 3 µl, (d) NEBNext Ultra II End Repair/dA-Tailing Buffer of 7 µl, (e) 100X NAD+ of 0.6 µl, and (f) 10 mM Tris HCl pH 8.5 buffer of 38.13 µl for a total volume of 60 µl. This is mixed gently via inversion and incubated at 20°C for 60 min and 65°C for 30 min in a thermocycler. 2. SPRI Cleanup Add to the above (a) Agencourt AMPure XP beads of 60 µl and let stand for 2 min followed by discarding supernatant while on magnet, (b) wash with 70% EtOH of 140 µl X 2, and (c) elute in Nuclease-free water of 31 µl. 3. Adaptor Ligation Add to (a) eluted End-prepped DNA above of 30 µl, (b) 1D Adapter Mix of 20 µl, and (c) NEB Instant Sticky End Ligase of 50 µl, for a total volume of 100 µl. This is mixed gently via inversion and incubated at 20°C for 10 min in a thermocycler. 4. Library Purification, with additional SPRI Cleanup Add to the above (a) Agencourt AMPure XP beads of 40 µl and let stand for 2 min followed by discarding supernatant while on magnet, (b) wash with Adaptor Bead Binding Buffer of 140 µl X 2, and (c) elute in Oxford Nanopore Elution Buffer of 25 µl for DNA sequencing. Library was run on an SQK-208 flow-cell.

F). SRA RUN_6 [Run 5 this study]
Trypsin_Resin_Barcode115_SQK-108

SRA Experiment SRX4909474; SRA Runs SRR8097531, 8082392

Total genomic DNA was extracted from blood using the Qiagen Blood & Cell Culture DNA Midi Kit (Hilden, Germany, cat# 13343), yielding 242 ng/µl provided in TE buffer. Sequencing was undertaken using the Oxford Nanopore MinIon (machine ID No. MN19030; flow-cell version SQK-108) with R9.4 chemistry and conducted using Minion protocol version GDE_9002_v108_revT_18Oct2016 for library preparation with modification. A nick-repair enzyme (NEBNext FFPE Repair Mix) to repair breaks in one side of the DNA double helix, which can prematurely shorten DNA reads was added. To increase library yield, NEBNext Ultra II End Repair/dA-Tailing Module was used to eliminate an additional SPRI (Solid Phase Reversible Immobilization) cleanup step, here applying Agencourt AMPure XP beads. To increase library yield, 2 µl of concentrated NEB T4 DNA ligase was added during adaptor ligation. 1. DNA Nick-Repair, Blunt-Ending, and End Repair Add in a clean low bind PCR tube, (a) ~ 2 µg genomic Tuatara DNA as described above of 8.27 µl, (b) NEBNext FFPE Repair Mix of 3 µl, (c) NEBNext Ultra II End Repair/dA-Tailing Mix of 3 µl, (d) NEBNext Ultra II End Repair/dA-Tailing Buffer of 7 µl, (e) 100X NAD+ of 0.6 µl, and (f) 10 mM Tris HCl pH 8.5 buffer of 38.13 µl for a total volume of 60 µl. This is mixed gently via inversion and incubated at 20°C for 60 min and 65°C for 30 min in a thermocycler. 2. Immobilized Trypsin-Resin in Place of SPRI Cleanup For this run a deviated protocol to increase library yield from wash cycles and avoid unnecessary DNA shearing from agitation, applied an enzymatic digest of end-repair enzymes in step 1 above replacing the use of SPRI beads. This was done using an activated trypsin. 2.1. To activate trypsin resin, resin was gently mixed adding 20 µl of resin to 100 µl of 10 mM Tris HCl pH 8.5 buffer in a 1.5 ml tube. This was inverted for mixing flowed by a 6000 rpm spin, with the supernatant discarded; this was repeated two additional times, with 20 µl of 10 mM Tris HCl pH 8.5 buffer added to the end product. 2.2. The (a) end-repaired DNA from step 1 of 60 µl, and (b) activated trypsin resin of 20 µl from step 2.1 were (c) incubate at 37°C for 30 min. Only 60 µl of the total volume of 80 µl was retained leaving 20 µl of pelleted resin behind. 3. Adaptor Ligation Add to (a) eluted End-prepped DNA above of 60 µl, (b) 1D Adapter Mix of 20 µl, and (c) NEB Instant Sticky End Ligase of 80 µl, for a total volume of 180 µl. This is mixed gently via inversion and incubated at 20°C for 10 min in a thermocycler. 4. Library Purification, with additional SPRI Cleanup Add to the above (a) Agencourt AMPure XP beads of 64 µl and let stand for 2 min followed by discarding supernatant while on magnet, (b) wash with Adaptor Bead Binding Buffer of 140 µl X 2, and (c) elute in Oxford Nanopore Elution Buffer of 25 µl for DNA sequencing. Library was run on an SQK-108 flow-cell.

G). SRA RUN_7 [Not used in this study; mtDNA purged] 2D_Trypsin_Resin_Barcode113_FLO-MIN106

SRA Experiment SRX4909475; SRA Runs SRR8097530, 8082391

Total genomic DNA was extracted from blood using the Qiagen Blood & Cell Culture DNA Mini Kit (Hilden, Germany, cat# 13323), yielding 150 ng/µl provided in TE buffer. Sequencing was undertaken using the Oxford Nanopore MinIon (machine ID No. MN19030; flow-cell version FLO-MIN106) with R9.4 chemistry and conducted using Minion protocol version GDE_9002_v108_revT_18Oct2016 for library preparation with modification. A nick-repair enzyme (NEBNext FFPE Repair Mix) to repair breaks in one side of the DNA double helix, which can prematurely shorten DNA reads was added. To increase library yield, NEBNext Ultra II End Repair/dA-Tailing Module was used to eliminate an additional SPRI (Solid Phase Reversible Immobilization) cleanup step, here applying Agencourt AMPure XP beads. To increase library yield, 2 µl of concentrated NEB T4 DNA ligase was added during adaptor ligation. 1. DNA Nick-Repair, Blunt-Ending, and End Repair Add in a clean low bind PCR tube, (a) ~ 2 µg genomic Tuatara DNA as described above of 8.27 µl, (b) NEBNext Ultra II End Repair/dA-Tailing Mix of 3 µl, (c) NEBNext Ultra II End Repair/dA-Tailing Buffer of 7 µl, and (d) 10 mM Tris HCl pH 8.5 buffer of 41.73 µl for a total volume of 60 µl. This is mixed gently via inversion and incubated at 20°C for 60 min and 65°C for 30 min in a thermocycler. 2. Immobilized Trypsin-Resin in Place of SPRI Cleanup For this run a deviated protocol to increase library yield from wash cycles and avoid unnecessary DNA shearing from agitation, applied an enzymatic digest of end-repair enzymes in step 1 above replacing the use of SPRI beads. This was done using an activated trypsin. 2.1. To activate trypsin resin, resin was gently mixed adding 20 µl of resin to 100 µl of 10 mM Tris HCl pH 8.5 buffer in a 1.5 ml tube. This was inverted for mixing flowed by a 6000 rpm spin, with the supernatant discarded; this was repeated two additional times, with 20 µl of 10 mM Tris HCl pH 8.5 buffer added to the end product. 2.2. The (a) end-repaired DNA from step 1 of 60 µl, and (b) activated trypsin resin of 20 µl from step 2.1 were (c) incubate at 37°C for 30 min. Only 60 µl of the total volume of 80 µl was retained leaving 20 µl of pelleted resin behind. 3. 2D Adaptor Ligation Add to (a) eluted End-prepped DNA above of 30 µl, (b) 2D Adapter Mix of 10 µl, (c) dH2O of 5 µl, (d) HP Adapter of 2 µl with (d) NEB Instant Sticky End Ligase of 50 µl and (e) incubated for 10 min at room temp; followed with (f) HP tether of 1 µl and (h) additional incubation at room temp for 10 min. The total yield is 100 µl for this step. 4. Library Purification requires two steps. 4.1. MyOne C1 Streptavidin beads preparation Add in a 1.5 ml Eppendorf DNA LoBind tube (a) MyOne C1 Streptavidin beads of 50 µl and (b) pellet beads on magnet for 2 min; (c) discard supernatant and (d) add Oxford Nanopore Bead Binding Buffer of 140 µl; (e) vortex until homogeneous and (f) pellet on a magnet for 2 min; (g) discard supernatant and (h) repeat Oxford Nanopore Bead Binding Buffer wash step of 140 µl with pelleting on a magnet for 2 min X 2; (i) add Bead Binding Buffer of 100 µl and (j) label tube as Washed Beads for binding step. 4.2. Purification of Library using Streptavidin Beads For binding add (a) Washed Beads from previous step 4.1 of 100 µl to the tube containing the Ligated DNA in step 3 of 100 µl and (b) incubate at room temperature for 5 min; for elution add (c) Oxford Nanopore elution buffer to DNA-bound beads of 25 µl, (d) incubate tube on hot

block at 37°C for 10 min, (e) pellet beads on a magnet for 2 min, and (f) transfer supernatant containing library into a clean 1.5 ml Eppendorf DNA LoBind tube for DNA sequencing. Library was run on a FLO-MIN106 flow-cell.

H). SRA RUN_8 [Not used in this study; mtDNA purged] 2D_Trypsin_Resin_Ligase_Barcode113_FLO-MIN106-Half_Run

SRA Experiment SRX4909476; SRA Runs SRR8097529, 8097528, 8097527, 8082390

Total genomic DNA was extracted from blood using the Qiagen Blood & Cell Culture DNA Mini Kit (Hilden, Germany, cat# 13323), yielding 150 ng/µl provided in TE buffer. Sequencing was undertaken using the Oxford Nanopore MinIon (machine ID No. MN19030; flow-cell version FLO-MIN106) with R9.4 chemistry and conducted using Minion protocol version GDE_9002_v108_revT_18Oct2016 for library preparation with modification. A nick-repair enzyme (NEBNext FFPE Repair Mix) to repair breaks in one side of the DNA double helix, which can prematurely shorten DNA reads was added. To increase library yield, NEBNext Ultra II End Repair/dA-Tailing Module was used to eliminate an additional SPRI (Solid Phase Reversible Immobilization) cleanup step, here applying Agencourt AMPure XP beads. To increase library yield, 2 µl of concentrated NEB T4 DNA ligase was added during adaptor ligation. 1. DNA Nick-Repair, Blunt-Ending, and End Repair Add in a clean low bind PCR tube, (a) ~ 2 µg genomic Tuatara DNA as described above of 8.27 µl, (b) NEBNext Ultra II End Repair/dA-Tailing Mix of 3 µl, (c) NEBNext Ultra II End Repair/dA-Tailing Buffer of 7 µl, and (d) 10 mM Tris HCl pH 8.5 buffer of 41.73 µl for a total volume of 60 µl. This is mixed gently via inversion and incubated at 20°C for 60 min and 65°C for 30 min in a thermocycler. 2. Immobilized Trypsin-Resin in Place of SPRI Cleanup For this run a deviated protocol to increase library yield from wash cycles and avoid unnecessary DNA shearing from agitation, applied an enzymatic digest of end-repair enzymes in step 1 above replacing the use of SPRI beads. This was done using an activated trypsin. 2.1. To activate trypsin resin, resin was gently mixed adding 20 µl of resin to 100 µl of 10 mM Tris HCl pH 8.5 buffer in a 1.5 ml tube. This was inverted for mixing flowed by a 6000 rpm spin, with the supernatant discarded; this was repeated two additional times, with 20 µl of 10 mM Tris HCl pH 8.5 buffer added to the end product. 2.2. The (a) end-repaired DNA from step 1 of 60 µl, and (b) activated trypsin resin of 20 µl from step 2.1 were (c) incubate at 37°C for 30 min. Only 60 µl of the total volume of 80 µl was retained leaving 20 µl of pelleted resin behind. 3. 2D Adaptor Ligation Add to (a) eluted End-prepped DNA above of 30 µl, (b) 2D Adapter Mix of 10 µl, (c) dH2O of 5 µl, (d) HP Adapter of 2 µl with (d) NEB Instant Sticky End Ligase of 50 µl and (e) incubated for 10 min at room temp; followed with (f) HP tether of 1 µl and (g) 2 µl of concentrated NEB T4 DNA ligase added with (h) additional incubation at room temp for 10 min. The 2 µl of concentrated NEB T4 DNA ligase was added during adaptor ligation with the HP Tether to increase library yield. The total yield is 100 µl for this step. 4. Library Purification requires two steps. 4.1. MyOne C1 Streptavidin beads preparation Add in a 1.5 ml Eppendorf DNA LoBind tube (a) MyOne C1 Streptavidin beads of 50 µl and (b) pellet beads on magnet for 2 min; (c) discard supernatant and (d) add Oxford Nanopore Bead Binding Buffer of 140 µl; (e) vortex until homogeneous and (f) pellet on a magnet for 2 min; (g) discard supernatant

and (h) repeat Oxford Nanopore Bead Binding Buffer wash step of 140 µl with pelleting on a magnet for 2 min X 2; (i) add Bead Binding Buffer of 100 µl and (j) label tube as Washed Beads for binding step. 4.2. Purification of Library using Streptavidin Beads For binding add (a) Washed Beads from previous step 4.1 of 100 µl to the tube containing the Ligated DNA in step 3 of 100 µl and (b) incubate at room temperature for 5 min; for elution add (c) Oxford Nanopore elution buffer to DNA-bound beads of 25 µl, (d) incubate tube on hot block at 37°C for 10 min, (e) pellet beads on a magnet for 2 min, and (f) transfer supernatant containing library into a clean 1.5 ml Eppendorf DNA LoBind tube for DNA sequencing. Library was run on a FLO-MIN106 flow-cell.

I). SRA RUN_9 [Not used in this study; mtDNA purged] 2D_Trypsin_Resin_rSAP_Kinase_Ligase_Barcode113_FLO-MIN106

SRA Experiment SRX4909477; SRA Runs SRR8097526, 8097525, 8097524, 8082389

Total genomic DNA was extracted from blood using the Qiagen Blood & Cell Culture DNA Mini Kit (Hilden, Germany, cat# 13323), yielding 150 ng/µl provided in TE buffer. Sequencing was undertaken using the Oxford Nanopore MinIon (machine ID No. MN19030; flow-cell version FLO-MIN106) with R9.4 chemistry and conducted using Minion protocol version GDE_9002_v108_revT_18Oct2016 for library preparation with modification. A nick-repair enzyme (NEBNext FFPE Repair Mix) to repair breaks in one side of the DNA double helix, which can prematurely shorten DNA reads was added. To increase library yield, NEBNext Ultra II End Repair/dA-Tailing Module was used to eliminate an additional SPRI (Solid Phase Reversible Immobilization) cleanup step, here applying Agencourt AMPure XP beads. To increase library yield, 2 µl of concentrated NEB T4 DNA ligase was added during adaptor ligation.1. DNA Nick-Repair, Blunt-Ending, and End Repair Add in a clean low bind PCR tube, (a) ~ 2 µg genomic Tuatara DNA as described above of 8.27 µl, (b) NEBNext FFPE Repair Mix of 3 µl, (c) NEBNext Ultra II End Repair/dA-Tailing Mix of 3 µl, (d) NEBNext Ultra II End Repair/dA-Tailing Buffer of 7 µl, (e) 100X NAD+ of 0.6 µl, and (f) 10 mM Tris HCl pH 8.5 buffer of 38.13 µl for a total volume of 60 µl. This is mixed gently via inversion and incubated at 20°C for 60 min and 65°C for 30 min in a thermocycler. An additional step (g) added 3 µl of NEB Antartic Phosphatase and 6 µl of NEB Antartic Phosphatase buffer containing Zn2+ for enzymatic activation and is also mixed gently via inversion and incubated at 37°C for 30 min in a thermocycler. 2. Immobilized Trypsin-Resin in Place of SPRI Cleanup For this run a deviated protocol to increase library yield from wash cycles and avoid unnecessary DNA shearing from agitation, applied an enzymatic digest of end-repair enzymes in step 1 above replacing the use of SPRI beads. This was done using an activated trypsin. 2.1. To activate trypsin resin, resin was gently mixed adding 20 µl of resin to 100 µl of 10 mM Tris HCl pH 8.5 buffer in a 1.5 ml tube. This was inverted for mixing flowed by a 6000 rpm spin, with the supernatant discarded; this was repeated two additional times, with 20 µl of 10 mM Tris HCl pH 8.5 buffer added to the end product. 2.2. The (a) end-repaired DNA from step 1 of 60 µl, and (b) activated trypsin resin of 20 µl from step 2.1 were (c) incubate at 37°C for 30 min. Only 60 µl of the total volume of 80 µl was retained leaving 20 µl of pelleted resin behind. 3. An additional step added (a) 2 µl of T4 Polynucleotide Kinase, (b) 10 µl of T4

Polynucleotide Kinase buffer, and 10 µl of 10 mM ATP. This is mixed gently via inversion and incubated at 37°C for 30 min in a thermocycler. 4. 2D Adaptor Ligation Add to (a) eluted End-prepped DNA above of 30 µl, (b) 2D Adapter Mix of 10 µl, (c) dH2O of 5 µl, (d) HP Adapter of 2 µl with (d) NEB Instant Sticky End Ligase of 50 µl and (e) incubated for 10 min at room temp; followed with (f) HP tether of 1 µl and (g) 2 µl of concentrated NEB T4 DNA ligase added with (h) additional incubation at room temp for 10 min. The 2 µl of concentrated NEB T4 DNA ligase was added during adaptor ligation with the HP Tether to increase library yield. The total yield is 100 µl for this step. 5. Library Purification requires two steps. 5.1. MyOne C1 Streptavidin beads preparation Add in a 1.5 ml Eppendorf DNA LoBind tube (a) MyOne C1 Streptavidin beads of 50 µl and (b) pellet beads on magnet for 2 min; (c) discard supernatant and (d) add Oxford Nanopore Bead Binding Buffer of 140 µl; (e) vortex until homogeneous and (f) pellet on a magnet for 2 min; (g) discard supernatant and (h) repeat Oxford Nanopore Bead Binding Buffer wash step of 140 µl with pelleting on a magnet for 2 min X 2; (i) add Bead Binding Buffer of 100 µl and (j) label tube as Washed Beads for binding step. 5.2. Purification of Library using Streptavidin Beads. For binding add (a) Washed Beads from previous step 5.1 of 100 µl to the tube containing the Ligated DNA in step 4 of 100 µl and (b) incubate at room temperature for 5 min; for elution add (c) Oxford Nanopore elution buffer to DNA-bound beads of 25 µl, (d) incubate tube on hot block at 37°C for 10 min, (e) pellet beads on a magnet for 2 min, and (f) transfer supernatant containing library into a clean 1.5 ml Eppendorf DNA LoBind tube for DNA sequencing. Library was run on a FLO-MIN106 flow-cell.

J). SRA RUN_10 [Run 6 this study; mtDNA purged] Trypsin_Resin_Barcode113_SQK-108

SRA Experiment SRX4909478; SRA Runs SRR8082388, 8097425, 8097426, 8097427

Total genomic DNA was extracted from blood using the Qiagen Blood & Cell Culture DNA Mini Kit (Hilden, Germany, cat# 13323), yielding 150 ng/µl provided in TE buffer. Sequencing was undertaken using the Oxford Nanopore MinIon (machine ID No. MN19030; flow-cell version SQK-108) with R9.4 chemistry and conducted using Minion protocol version GDE_9002_v108_revT_18Oct2016 for library preparation with modification. A nick-repair enzyme (NEBNext FFPE Repair Mix) to repair breaks in one side of the DNA double helix, which can prematurely shorten DNA reads was added. To increase library yield, NEBNext Ultra II End Repair/dA-Tailing Module was used to eliminate an additional SPRI (Solid Phase Reversible Immobilization) cleanup step, here applying Agencourt AMPure XP beads. To increase library yield, 2 µl of concentrated NEB T4 DNA ligase was added during adaptor ligation. 1. DNA Nick-Repair, Blunt-Ending, and End Repair Add in a clean low bind PCR tube, (a) ~ 2 µg genomic Tuatara DNA as described above of 8.27 µl, (b) NEBNext FFPE Repair Mix of 3 µl, (c) NEBNext Ultra II End Repair/dA-Tailing Mix of 3 µl, (d) NEBNext Ultra II End Repair/dA-Tailing Buffer of 7 µl, (e) 100X NAD+ of 0.6 µl, and (f) 10 mM Tris HCl pH 8.5 buffer of 38.13 µl for a total volume of 60 µl. This is mixed gently via inversion and incubated at 20°C for 60 min and 65°C for 30 min in a thermocycler. 2. Immobilized Trypsin-Resin in Place of SPRI Cleanup For this run a deviated protocol to increase

library yield from wash cycles and avoid unnecessary DNA shearing from agitation, applied an enzymatic digest of end-repair enzymes in step 1 above replacing the use of SPRI beads. This was done using an activated trypsin. 2.1. To activate trypsin resin, resin was gently mixed adding 20 µl of resin to 100 µl of 10 mM Tris HCl pH 8.5 buffer in a 1.5 ml tube. This was inverted for mixing flowed by a 6000 rpm spin, with the supernatant discarded; this was repeated two additional times, with 20 µl of 10 mM Tris HCl pH 8.5 buffer added to the end product. 2.2. The (a) end-repaired DNA from step 1 of 60 µl, and (b) activated trypsin resin of 20 µl from step 2.1 were (c) incubate at 37°C for 30 min. Only 60 µl of the total volume of 80 µl was retained leaving 20 µl of pelleted resin behind. 3. Adaptor Ligation Add to (a) eluted End-prepped DNA above of 60 µl, (b) 1D Adapter Mix of 20 µl, and (c) NEB Instant Sticky End Ligase of 80 µl, for a total volume of 180 µl. This is mixed gently via inversion and incubated at 20°C for 10 min in a thermocycler. 4. Library Purification, with additional SPRI Cleanup Add to the above (a) Agencourt AMPure XP beads of 64 µl and let stand for 2 min followed by discarding supernatant while on magnet, (b) wash with Adaptor Bead Binding Buffer of 140 µl X 2, and (c) elute in Oxford Nanopore Elution Buffer of 25 µl for DNA sequencing. Library was run on an SQK-108 flow-cell.

K). SRA RUN_11 [Not used in this study; mtDNA purged] Trypsin_Resin_Nick_Repair_Barcode113_SQK-108_A

SRA Experiment SRX4909467; SRA Run SRR8082399, 8097536, 8097537, 8097538

Total genomic DNA was extracted from blood using the Qiagen Blood & Cell Culture DNA Mini Kit (Hilden, Germany, cat# 13323), yielding 150 ng/µl provided in TE buffer. Sequencing was undertaken using the Oxford Nanopore MinIon (machine ID No. MN19030; flow-cell version FLO-MIN106) with R9.4 chemistry and conducted using Minion protocol version GDE_9002_v108_revT_18Oct2016 for library preparation with modification. A nick-repair enzyme (NEBNext FFPE Repair Mix) to repair breaks in one side of the DNA double helix, which can prematurely shorten DNA reads was added. To increase library yield, NEBNext Ultra II End Repair/dA-Tailing Module was used to eliminate an additional SPRI (Solid Phase Reversible Immobilization) cleanup step, here applying Agencourt AMPure XP beads. To increase library yield, 2 µl of concentrated NEB T4 DNA ligase was added during adaptor ligation. 1. DNA Nick-Repair, Blunt-Ending, and End Repair Add in a clean low bind PCR tube, (a) ~ 2 µg genomic Tuatara DNA as described above of 8.27 µl, (b) NEBNext FFPE Repair Mix of 3 µl, (c) NEBNext Ultra II End Repair/dA-Tailing Mix of 3 µl, (d) NEBNext Ultra II End Repair/dA-Tailing Buffer of 7 µl, (e) 100X NAD+ of 0.6 µl, and (f) 10 mM Tris HCl pH 8.5 buffer of 38.13 µl for a total volume of 60 µl. This is mixed gently via inversion and incubated at 20°C for 60 min and 65°C for 30 min in a thermocycler. 2. Immobilized Trypsin-Resin in Place of SPRI Cleanup For this run a deviated protocol to increase library yield from wash cycles and avoid unnecessary DNA shearing from agitation, applied an enzymatic digest of end-repair enzymes in step 1 above replacing the use of SPRI beads. This was done using an activated trypsin. 2.1. To activate trypsin resin, resin was gently mixed adding 20 µl of resin to 100 µl of 10 mM Tris HCl pH 8.5 buffer in a 1.5 ml tube. This was inverted for mixing flowed by a 6000 rpm spin, with the

supernatant discarded; this was repeated two additional times, with 20 µl of 10 mM Tris HCl pH 8.5 buffer added to the end product. 2.2. The (a) end-repaired DNA from step 1 of 60 µl, and (b) activated trypsin resin of 20 µl from step 2.1 were (c) incubate at 37°C for 30 min. Only 60 µl of the total volume of 80 µl was retained leaving 20 µl of pelleted resin behind. 3. Adaptor Ligation Add to (a) eluted End-prepped DNA above of 60 µl, (b) 1D Adapter Mix of 20 µl, and (c) NEB Instant Sticky End Ligase of 80 µl, for a total volume of 180 µl. This is mixed gently via inversion and incubated at 20°C for 10 min in a thermocycler. 4. Library Purification, with additional SPRI Cleanup Add to the above (a) Agencourt AMPure XP beads of 64 µl and let stand for 2 min followed by discarding supernatant while on magnet, (b) wash with Adaptor Bead Binding Buffer of 140 µl X 2, and (c) elute in Oxford Nanopore Elution Buffer of 25 µl for DNA sequencing. Library was run on a FLO-MIN106 flow-cell.

L). SRA RUN_12 [Not used in this study; mtDNA purged] Trypsin_Resin_Nick_Repair_Barcode113_SQK-108_B

SRA Experiment SRX4909468; SRA Run SRR8082398, 8097533, 8097534, 8097535

Total genomic DNA was extracted from blood using the Qiagen Blood & Cell Culture DNA Mini Kit (Hilden, Germany, cat# 13323), yielding 150 ng/µl provided in TE buffer. Sequencing was undertaken using the Oxford Nanopore MinIon (machine ID No. MN19030; flow-cell version FLO-MIN106) with R9.4 chemistry and conducted using Minion protocol version GDE_9002_v108_revT_18Oct2016 for library preparation with modification. A nick-repair enzyme (NEBNext FFPE Repair Mix) to repair breaks in one side of the DNA double helix, which can prematurely shorten DNA reads was added. To increase library yield, NEBNext Ultra II End Repair/dA-Tailing Module was used to eliminate an additional SPRI (Solid Phase Reversible Immobilization) cleanup step, here applying Agencourt AMPure XP beads. To increase library yield, 2 µl of concentrated NEB T4 DNA ligase was added during adaptor ligation. 1. DNA Nick-Repair, Blunt-Ending, and End Repair Add in a clean low bind PCR tube, (a) ~ 2 µg genomic Tuatara DNA as described above of 8.27 µl, (b) NEBNext FFPE Repair Mix of 3 µl, (c) NEBNext Ultra II End Repair/dA-Tailing Mix of 3 µl, (d) NEBNext Ultra II End Repair/dA-Tailing Buffer of 7 µl, (e) 100X NAD+ of 0.6 µl, and (f) 10 mM Tris HCl pH 8.5 buffer of 38.13 µl for a total volume of 60 µl. This is mixed gently via inversion and incubated at 20°C for 60 min and 65°C for 30 min in a thermocycler. 2. Immobilized Trypsin-Resin in Place of SPRI Cleanup For this run a deviated protocol to increase library yield from wash cycles and avoid unnecessary DNA shearing from agitation, applied an enzymatic digest of end-repair enzymes in step 1 above replacing the use of SPRI beads. This was done using an activated trypsin. 2.1. To activate trypsin resin, resin was gently mixed adding 20 µl of resin to 100 µl of 10 mM Tris HCl pH 8.5 buffer in a 1.5 ml tube. This was inverted for mixing flowed by a 6000 rpm spin, with the supernatant discarded; this was repeated two additional times, with 20 µl of 10 mM Tris HCl pH 8.5 buffer added to the end product. 2.2. The (a) end-repaired DNA from step 1 of 60 µl, and (b) activated trypsin resin of 20 µl from step 2.1 were (c) incubate at 37°C for 30 min. Only 60 µl of the total volume of 80 µl was retained leaving 20 µl of pelleted resin behind. 3. Adaptor Ligation Add to (a) eluted End-prepped DNA above of 60 µl, (b)

1D Adapter Mix of 20 µl, and (c) NEB Instant Sticky End Ligase of 80 µl, for a total volume of 180 µl. This is mixed gently via inversion and incubated at 20°C for 10 min in a thermocycler. 4. Library Purification, with additional SPRI Cleanup Add to the above (a) Agencourt AMPure XP beads of 64 µl and let stand for 2 min followed by discarding supernatant while on magnet, (b) wash with Adaptor Bead Binding Buffer of 140 µl X 2, and (c) elute in Oxford Nanopore Elution Buffer of 25 µl for DNA sequencing. Library was run on a FLO-MIN106 flow-cell.

M). SRA RUN_13 [Not used in this study; mtDNA purged] 10kb_Shear_Barcode113_FLO-MIN106

SRA Experiment SRX4909466; SRA Run SRR8082400, 8097539, 8097540, 8097541

Total genomic DNA was extracted from blood using the Qiagen Blood & Cell Culture DNA Mini Kit (Hilden, Germany, cat# 13323), yielding 150 ng/µl provided in TE buffer. Sequencing was undertaken using the Oxford Nanopore MinIon (machine ID No. MN19030; flow-cell version FLO-MIN106) with R9.4 chemistry and conducted using Minion protocol version GDE_9002_v108_revT_18Oct2016 for library preparation with modification. A nick-repair enzyme (NEBNext FFPE Repair Mix) to repair breaks in one side of the DNA double helix, which can prematurely shorten DNA reads was added. To increase library yield, NEBNext Ultra II End Repair/dA-Tailing Module was used to eliminate an additional SPRI (Solid Phase Reversible Immobilization) cleanup step, here applying Agencourt AMPure XP beads. To increase library yield, 2 µl of concentrated NEB T4 DNA ligase was added during adaptor ligation. 10 kb DNA Shearing DNA was sheared to a target of 10 kb from ~ 2 µg of Tuatara DNA using a Covaris g-tube. It is noteworthy that while subjecting DNA to shearing a direct following step implementing nick-repair was applied. Final products from this shearing procedure required volume adjustments between input DNA (from shearing in buffer) and 10 mM Tris HCl pH 8.5 buffer in the starting protocol of step 1 below to keep relative concentrations equivalent. 1. DNA Nick-Repair, Blunt-Ending, and End Repair Add in a clean low bind PCR tube, (a) ~ 2 µg genomic Tuatara DNA as described above of 8.27 µl, (b) NEBNext FFPE Repair Mix of 3 µl, (c) NEBNext Ultra II End Repair/dA-Tailing Mix of 3 µl, (d) NEBNext Ultra II End Repair/dA-Tailing Buffer of 7 µl, (e) 100X NAD+ of 0.6 µl, and (f) 10 mM Tris HCl pH 8.5 buffer of 38.13 µl for a total volume of 60 µl. This is mixed gently via inversion and incubated at 20°C for 60 min and 65°C for 30 min in a thermocycler. 2. SPRI Cleanup Add to the above (a) Agencourt AMPure XP beads of 60 µl and let stand for 2 min followed by discarding supernatant while on magnet, (b) wash with 70% EtOH of 140 µl X 2, and (c) elute in Nuclease-free water of 31 µl. 3. Adaptor Ligation Add to (a) eluted End-prepped DNA above of 30 µl, (b) 1D Adapter Mix of 20 µl, and (c) NEB Instant Sticky End Ligase of 50 µl, for a total volume of 100 µl. This is mixed gently via inversion and incubated at 20°C for 10 min in a thermocycler. 4. Library Purification, with additional SPRI Cleanup Add to the above (a) Agencourt AMPure XP beads of 40 µl and let stand for 2 min followed by discarding supernatant while on magnet, (b) wash with Adaptor Bead Binding Buffer of 140 µl X 2, and (c) elute in Oxford Nanopore Elution Buffer of 25 µl for DNA sequencing. Library was run on an SQK-108 flow-cell.

N). SRA RUN_14 [Run 7 this study]
1D-ligation-Trypsin-resin_1

SRA Experiment SRX7284173; SRA Run SRR10604630

Total genomic DNA was extracted from blood using a phenol-chloroform extraction, yielding 78.7 ng/µl after resuspending in TE buffer. Sequencing was undertaken using the Oxford Nanopore MinIon (machine ID No. MN26033; flow-cell version SQK-LSK109) with R9.4 chemistry and conducted using Minion protocol version 1d-gDNA-sqk-lsk109-GDE_9063_v109_revC_23May2018 for library preparation with modification. A nick-repair enzyme (NEBNext FFPE Repair Mix) to repair breaks in one side of the DNA double helix, which can prematurely shorten DNA reads was added. To increase library yield, NEBNext Ultra II End Repair/dA-Tailing Module was used to eliminate an additional SPRI (Solid Phase Reversible Immobilization) cleanup step, here applying Agencourt AMPure XP beads. To increase library yield, 2 µl of concentrated NEB T4 DNA ligase was added during adaptor ligation. 1. DNA Nick-Repair, Blunt-Ending, and End Repair Add in a clean low bind PCR tube, (a) ~ 2 µg genomic Tuatara DNA as described above was eluted to 37 µl in TE buffer, (b) NEBNext FFPE Repair Buffer of 3.5 µl, (c) NEBNext FFPE Repair Mix of 2 µl (c) NEBNext Ultra II End Repair/dA-Tailing Mix of 3 µl, (d) NEBNext Ultra II End Repair/dA-Tailing Buffer of 2 µl, (e) Oxford Nanopore DNA CS 1 µl for a total volume of 50 µl. This is mixed gently via inversion and incubated at 20°C for 60 min and 65°C for 30 min in a thermocycler. 2. Immobilized Trypsin-Resin in Place of SPRI Cleanup For this run a deviated protocol to increase library yield from wash cycles and avoid unnecessary DNA shearing from agitation, applied an enzymatic digest of end-repair enzymes in step 1 above replacing the use of SPRI beads. This was done using an activated trypsin. 2.1. To activate trypsin resin, resin was gently mixed adding 20 µl of resin to 120 µl of 10 mM Tris HCl pH 8.5 buffer in a 1.5 ml tube. This was inverted for mixing flowed by a 6000 rpm spin, with the supernatant discarded; this was repeated two additional times, with 20 µl of 10 mM Tris HCl pH 8.5 buffer added to the end product. 2.2. The (a) end-repaired DNA from step 1 of 50 µl, and (b) activated trypsin resin of 20 µl from step 2.1 were (c) incubate at 37°C for 30 min. Only 60 µl of the total volume of 70 µl was retained leaving 10 µl of pelleted resin behind. 3. Adaptor Ligation Add to (a) eluted End-prepped DNA above of 60 µl, (b) Oxford Nanopore Adapter Mix (AMX) of 5 µl, and (c) NEB T4 Ligase of 10 µl, (d) Ligation Buffer (LNB) 25.0 µl, for a total volume of 100.0 µl. This is mixed gently via inversion and incubated at 20°C for 10 min in a thermocycler. 4. Library Purification, with additional SPRI Cleanup. Add to the above (a) Agencourt AMPure XP beads of 50 µl and let stand for 2 min followed by discarding supernatant while on magnet, (b) wash with Long Fragment Buffer of 100 µl X 2, and (c) elute in Oxford Nanopore Elution Buffer of 15 µl for DNA sequencing. Library was run on an SQK-LSK109 flow-cell.

O). SRA RUN_15 [Run 8 this study]
1D-ligation-Trypsin-resin_2

SRA Experiment SRX7284174; SRA Run SRR10604629

Total genomic DNA was extracted from blood using an enzymatic based DNA extraction, yielding 131.4 ng/µl after resuspending in TE buffer. Sequencing was undertaken using the Oxford Nanopore MinIon (machine ID No. MN26033; flow-cell version SQK-LSK109) with R9.4 chemistry and conducted using Minion protocol version 1d-gDNA-sqk-lsk109-GDE_9063_v109_revC_23May2018 for library preparation with modification. A nick-repair enzyme (NEBNext FFPE Repair Mix) to repair breaks in one side of the DNA double helix, which can prematurely shorten DNA reads was added. To increase library yield, NEBNext Ultra II End Repair/dA-Tailing Module was used to eliminate an additional SPRI (Solid Phase Reversible Immobilization) cleanup step, here applying Agencourt AMPure XP beads. To increase library yield, 2 µl of concentrated NEB T4 DNA ligase was added during adaptor ligation. 1. DNA Nick-Repair, Blunt-Ending, and End Repair Add in a clean low bind PCR tube, (a) ~ 2 µg genomic Tuatara DNA as described above was eluted to 37 µl in TE buffer, (b) NEBNext FFPE Repair Buffer of 3.5 µl, (c) NEBNext FFPE Repair Mix of 2 µl (c) NEBNext Ultra II End Repair/dA-Tailing Mix of 3 µl, (d) NEBNext Ultra II End Repair/dA-Tailing Buffer of 2 µl, (e) Oxford Nanopore DNA CS 1 µl for a total volume of 50 µl. This is mixed gently via inversion and incubated at 20°C for 60 min and 65°C for 30 min in a thermocycler. 2. Immobilized Trypsin-Resin in Place of SPRI Cleanup For this run a deviated protocol to increase library yield from wash cycles and avoid unnecessary DNA shearing from agitation, applied an enzymatic digest of end-repair enzymes in step 1 above replacing the use of SPRI beads. This was done using an activated trypsin. 2.1. To activate trypsin resin, resin was gently mixed adding 20 µl of resin to 120 µl of 10 mM Tris HCl pH 8.5 buffer in a 1.5 ml tube. This was inverted for mixing flowed by a 6000 rpm spin, with the supernatant discarded; this was repeated two additional times, with 20 µl of 10 mM Tris HCl pH 8.5 buffer added to the end product. 2.2. The (a) end-repaired DNA from step 1 of 50 µl, and (b) activated trypsin resin of 20 µl from step 2.1 were (c) incubate at 37°C for 30 min. Only 60 µl of the total volume of 70 µl was retained leaving 10 µl of pelleted resin behind. 3. Adaptor Ligation Add to (a) eluted End-prepped DNA above of 60 µl, (b) Oxford Nanopore Adapter Mix (AMX) of 5 µl, and (c) NEB T4 Ligase of 10 µl, (d) Ligation Buffer (LNB) 25.0 µl, for a total volume of 100.0 µl. This is mixed gently via inversion and incubated at 20°C for 10 min in a thermocycler. 4. Library Purification, with additional SPRI Cleanup Add to the above (a) Agencourt AMPure XP beads of 50 µl and let stand for 2 min followed by discarding supernatant while on magnet, (b) wash with Long Fragment Buffer of 100 µl X 2, and (c) elute in Oxford Nanopore Elution Buffer of 15 µl for DNA sequencing. Library was run on a SQK-LSK109 flow-cell.

Supplementary Method 4

Data deposition of raw PacBio sequencing reads in the NCBI Sequence Read Archive (SRA) database as PRJNA445603. DNA sequence reads from two Tuatara samples are sequenced with PacBio raw reads deposited in the NCBI Sequence Read Archive (SRA) as SRP140116/PRJNA445603. (1) LAI, Passive Integrated Transponder (PIT) 982 000167713537 originating from Lady Alice Island, Hen and Chickens Islands, New Zealand (Biosample SAMN08793959), and (2) SI-3, St. Louis Zoo SLZ 19079 originating from Stephens Island, Marlborough Sounds, Marlborough Province, New Zealand, (Biosample SAMN10598680, ISIS 103796).

The LAI sample was subjected to 4 PacBio runs. The first two are listed as A and B below and were used. Two additional LAI PacBio runs listed under C below were done on DNA extracted by Dovetail (Santa Cruz, CA) that purged mtDNA, and no mtDNA was detected. Two PacBio runs listed under D below were done on SI-3 providing a draft complete mt-genome available in GenBank as MN864230.

A). WGS sequencing of Sphenodon punctatus, LAI, Biosample SAMN08793959 5Kb Shear: LASmith_gDNA_5kb

SRA Experiment SRX5334279; SRA Run SRR8530961

High-molecular-weight genomic DNA was extracted using the Qiagen Genomic Tip DNA extraction kit (genomic-tip column 100/G) from 100 µl of snap-frozen blood according to the manufacturer's directions, and performed at the Laboratories of Analytical Biology at the Smithsonian Institutions National Museum of Natural History (NMNH). A PacBio SMRTbell library was prepared using the SMRTBell Express Template Preparation Kit (Pacific Biosciences) following the PacBio 15-kb template preparation protocol, with a minimum size cutoff of 5kb size selection used in the size selection stage with the Sage Science BluePippin system. The prepared library was run on the PacBio Sequel platform (Pacific Biosciences) using version 2.1 chemistry. The Single Molecule Real-Time (SMRT) Cell was sequenced on a SMRT cell with 360min movie lengths.

B). WGS sequencing of Sphenodon punctatus, LAI, Biosample SAMN08793959 12kb Shear: LASmith_gDNA_12Kb

SRA Experiment SRX5334280; SRA Run SRR8530960

High-molecular-weight genomic DNA was extracted using the Qiagen Genomic Tip DNA extraction kit (genomic-tip column 100/G) from 100 µl of snap-frozen blood according to the manufacturer's directions, and performed at the Laboratories of Analytical Biology at the Smithsonian Institutions National Museum of Natural History (NMNH). A PacBio SMRTbell library was prepared using the SMRTBell Express Template Preparation Kit (Pacific Biosciences) following the PacBio 15-kb template preparation protocol, with a minimum size cutoff of 12kb size selection used in the size

selection stage with the Sage Science BluePippin system. The prepared library was run on the PacBio Sequel platform (Pacific Biosciences) using version 2.1 chemistry. The Single Molecule Real-Time (SMRT) Cell was sequenced on a SMRT cell with 360min movie lengths.

C). WGS sequencing of Sphenodon punctatus, LAI, Biosample SAMN08793959

12 Kb Shear: LA0114_gDNA_12kb_1 [Not used in this study; mtDNA purged]
SRA Experiment SRX5334282; SRA Run SRR8530958

12 Kb Shear: LA0114_gDNA_12kb_2 [Not used in this study; mtDNA purged]
SRA Experiment SRX5334281; SRA Run SRR8530959

High-molecular-weight genomic DNA was extracted from ~100 µl of snap-frozen blood using proteinase K digestion with Phenol-Chloroform extraction, and performed at Dovetail Genomics (Scotts Valley, CA). A PacBio SMRTbell library was prepared using the SMRTBell Express Template Preparation Kit (Pacific Biosciences) following the PacBio 15-kb template preparation protocol, with a minimum size cutoff of 5kb size selection used in the size selection stage with the Sage Science BluePippin system. The prepared library was run on the PacBio Sequel platform (Pacific Biosciences) using version 2.1 chemistry. The Single Molecule Real-Time (SMRT) Cell was sequenced on a SMRT cell with 360min movie lengths.

D). WGS sequencing of Sphenodon punctatus, SI-3, Biosample SAMN10598680

12 Kb Shear: SLZ19079_12Kb_1
SRA Experiment SRX7298462; SRA Run SRR10619303

12 Kb Shear: SLZ19079_12Kb_2
SRA Experiment SRX7298463; SRA Run SRR10619302

High-molecular-weight genomic DNA was extracted using the Qiagen Genomic Tip DNA extraction kit (genomic-tip column 100/G) from ~20mg of liver tissue according to the manufacturer's directions, and was performed at the Laboratories of Analytical Biology at the Smithsonian Institutions National Museum of Natural History (NMNH). A PacBio SMRTbell library was prepared using the SMRTBell Express Template Preparation Kit (Pacific Biosciences) following the PacBio 15-kb template preparation protocol, with a minimum size cutoff of 5kb size selection used in the size selection stage with the Sage Science BluePippin system.The prepared library was run on the PacBio Sequel platform (Pacific Biosciences) using version 2.1 chemistry. The Single Molecule Real-Time (SMRT) Cell was sequenced with 360min movie lengths.

Supplementary Method 5

Evaluation of phylogenetic results in extant Tuatara populations and a duplicate molecule. In order to further evaluate whether the two Lady Alice Island (LAI) mt-genomic molecules are in fact not most recent common copies, a Wilcoxon signed-ranks test is conducted. Constraining the two LAI mt-genomic molecules as monophyletetic produces two shortest alternative trees of 1357 steps in length: Alternative Tree 1 (LAI M2, (LAI M1, ((((Poor Knights, ((S-1, S-2), (N Brother 1, N Brother 2))), Plate), (Cuvier, (Green Mercury, (((Mid Mercury 1, Mid Mercury 2), Stanley), Red Mercury)))), Hen))); Alternative Tree 2 (LAI M2, (LAI M1, ((((Poor Knights, ((S-1, S-2), (N Brother 1, N Brother 2))), Plate), Hen), (Cuvier, (Green Mercury, (((Mid Mercury 1, Mid Mercury 2), Stanley), Red Mercury)))))). When the shortest overall most parsimonious tree depicting a non-monphyletic grouping of LAI mt-molecules is compared with the two shortest alternative trees grouping the LAI mt-genomic molecules as monophyletic, these two alternative trees are rejected (tree 1, n=66, Z=3.7644, P <0.0002; tree 2, n=65, Z=3.8806, P<0.0001). This indicates that the two LAI mt-genomic molecules are indeed not related, and appear highly diverged as the phylogenetic tree suggests.

Supplementary Method 6

Statistical evaluation of duplicated putative Control Region sequences (NC1–3). In order to evaluate support for the non-monophyly of putative Control Region sequences (NC1–3) by region, Wilcoxon-signed ranks tests are conducted. When the two shortest overall most parsimonious trees (107 steps) depicting monphyly of sequences from each mt-genome are compared with the three shortest alternative trees (181 steps) grouping NC1 sequences as monophyletic, these three alternative trees are rejected (all, n=76, Z=8.1695, P <0.0001). When the two shortest overall most parsimonious trees (107 steps) depicting monphyly of sequences from each mt-genome are compared with the 15 shortest alternative trees (187 steps) grouping NC2 sequences as monophyletic, these 15 alternative trees are rejected (all, n=78, Z=8.7261, P <0.0001). When the two shortest overall most parsimonious trees (107 steps) depicting monphyly of sequences from each mt-genome are compared with the three shortest alternative trees (172 steps) grouping NC3 sequences as monophyletic, these three alternative trees are rejected (all, n=76, Z=8.1695, P <0.0001). Hence, constraining sequences from any one of the three non-coding blocks (NC1–3) to be monophyletic is statistically rejected.

Supplementary Method 7

Evaluation of mitochondrial encoded protein structure. Estimations were done in PAML-CodeML for synonymous (dS) and non-synonymous (dN) substitution rates and their fraction ω = dN/dS, with the LAI M2 lineage as the foreground branch and all mt-molecule-1 lineages as background branches. Specifically, we tested the null model of uniform selective pressure "M0", the nearly-neutral site model "M1a", the positive selection site model "M2a", the beta distribution site model of neutral evolution "M7", the beta distribution site model of positive selection "M8", the beta distribution site model of relaxation "M8a", the branch model assuming neutral evolution of the foreground "b_neut", the branch model with free evolution of the foreground "b_free", and – if the branch model comparisons were significant – the branch site models "bsA" and "bsA1" which assume positive selection and relaxation, respectively, at certain sites on some branches (Supplementary Table 1). The following models were tested against each other per gene using a likelihood ratio test (LRT): M1a and M2a (as well as M7 and M8) to detect positive selection as opposed to relaxation of selective pressures, and M8a and M8 to detect positive selection with increased power[50]. All these site models assume that the evolution rate is constant across the phylogenetic branches and only varies across sites. We therefore also compared M0 and b_free to test for different ω between branches, and b_neut and b_free to assess if the foreground branch does not evolve neutrally. If these models differed significantly from each other, we also compared M1 and bsA to test for relaxation at specific sites on some branches, and bsA1 and bsA to detect positive selection at specific sites on some branches (Supplementary Table 1). If any of the site or branch-site model comparisons were significant, Bayes Empirical Bayes (BEB) analysis[53] was performed and sites with a BEB score [Pr(ω >1)] higher than 95% were retained as sites under putative positive selection.

In general, PAML-CodeML identified a consistent pattern of purifying selection in all proteins, with an average ratio of ω = 0.096 ± 0.016 (Supplementary Table 2). The PAML-CodeML site models detected encoded ND2 to be under putative positive selection in the LAI M2 lineage (M8a vs M8; Supplementary Tables 2, 3). For multiple genes (encoding ND1, ATP6, COI, Cytb), we found evidence of variable selection across branches (M0 vs b_free, b_neut vs b_free), but subsequent branch-site models (bsA1 vs bsA) did not discover any individual sites under putative positive selection (Supplementary Table 2).

FEL is a maximum likelihood method that directly estimates dN and dS at each site and allows for variation in substitution rates across sites. This approach assumes that the selection pressure at each site is constant along the phylogenetic tree. As FEL is a fixed effects likelihood method as opposed to random effects models like PAML-CodeML, it is expected to be more accurate. Again, the LAI M2 lineage was selected as foreground branch. FEL detected sites under putative positive selection in six genes (encoding ND1: n=10; ND2: n=14; ND4: n=14; COII: n=3; COIII: n=3; Cytb: n=10; Table S3).

Supplementary Table 1. Summary of assumptions of the PAML site, branch and branch-site models, and of LRTs that can be performed to detect positive selection.

Model	Description	LRT
M0	site model; unique ω for all sites	
M1a	site model; assumes sites with ω <1 and with ω =1	
M2a	site model; assumes sites with ω <1, with ω =1, and with ω ≥1	vs M1a: test for positive selection at sites; sometimes lacks power
M7	site model; assumes ten categories of ω following a beta-distribution of sites, all at ω ≤1	
M8	site model; assumes ten categories of ω following a beta-distribution of sites, all at ω ≤1, and an additional category ω ≥1	vs M7: test for positive selection at sites; sometimes lacks power<br><br>vs M8a: test for positive selection at sites with increased power
M8a	site model; assumes ten categories of ω following a beta-distribution of sites, all at ω ≤1, and an additional category ω =1	
b_neut	branch model; assumes relaxation of selection	
b_free	branch site model; assumes positive selection	vs M0 to detect different ω across branches vs b_neut to detect non-neutral evolution on foreground branch
bsA1	branch model; assumes relaxation of selection	
bsA	branch site model; assumes positive selection only applied if branch models show significant difference	vs M1 to detect relaxation at specific sites on some branches<br>vs bsA1 to detect positive selection at specific sites on some branches

Supplementary Table 2. Summary of p-values across genes as obtained by LRT between models assuming absence (H0) and presence (H1) of positive selection. The ratios of non-synonymous to synonymous substitution rates ω per gene, across the phylogenetic tree (under M0) are indicated at the bottom of the table.

**H0**	**H1**	**ND1**	**ND2**	**ND3**	**ND4**	**ND4L**	**ND5**	**ND6**	**ATP6**	**ATP8**	**COI**	**COII**	**COIII**	**Cytb**
M1a	M2a	0.430	0.760	1	1	1	0.839	0.903	0.561	1	0.672	1	1	1
M7	M8	0.295	0.068	0.947	1	1	0.593	0.618	0.546	1	0.541	1	0.943	0.826
M8a	M8	0.195	0.036	1	0.924	1	0.523	0.657	0.278	1	0.386	1	0.844	0.546
M0	b_free	0.019	0.157	0.392	0.294	1	0.232	0.324	0.021	1	0.0002	0.269	0.48	0.00001
b_neut	b_free	0.019	0.532	0.772	0.294	1	0.232	0.324	0.021	0.632	0.0002	0.269	0.48	0.00001
M1	bsA	1	-	-	-	-	-	-	1	-	1	-	-	-
bsA1	bsA	1	-	-	-	-	-	-	0.997	-	1	-	-	-
**ω M0**		0.081	0.13	0.127	0.063	0.082	0.128	0.033	0.218	0.177	0.039	0.03	0.068	0.069

Supplementary Table 3. Summary of codon sites under putative positive selection (FDR ≤ 0.05). If a site is detected by FEL, stat refers to the LRT statistic, p-value to the p-value for this site, and BH[54] to the FDR-corrected p-values across sites per gene. If a site is detected by PAML with subsequent BEB analysis, stat refers to [Pr(ω >1)], and the p-value to p-value as obtained by LRT between models assuming absence and presence of positive selection, respectively.

gene	AA site	stat	p-value	BH
ND1	322	17.234	0.0001	0.003577778
ND1	78	15.795	0.0001	0.003577778
ND1	74	15.571	0.0001	0.003577778
ND1	50	13.046	0.0001	0.003577778
ND1	125	12.646	0.0001	0.003577778
ND1	285	15.62	0.0001	0.003577778
ND1	32	17.234	0.0001	0.003577778
ND1	139	15.663	0.0001	0.003577778
ND1	242	15.503	0.0001	0.003577778
ND1	253	11.633	0.001	0.0322
ND2	218	0.979	0.035554	N.A.
ND2	94	16.428	0.0001	0.00346
ND2	304	12.741	0.0001	0.00346
ND2	122	19.645	0.0001	0.00346
ND2	211	17.342	0.0001	0.00346
ND2	284	17.342	0.0001	0.00346
ND2	283	14.807	0.0001	0.00346
ND2	280	14.929	0.0001	0.00346
ND2	337	18.604	0.0001	0.00346
ND2	245	19.278	0.0001	0.00346
ND2	194	17.342	0.0001	0.00346
ND2	298	11.325	0.001	0.024714286
ND2	156	11.263	0.001	0.024714286
ND2	272	11.389	0.001	0.024714286
ND2	330	11.296	0.001	0.024714286
ND4	426	18.474	0.0001	0.003833333
ND4	36	18.471	0.0001	0.003833333
ND4	188	12.977	0.0001	0.003833333
ND4	78	13.348	0.0001	0.003833333
ND4	29	17.036	0.0001	0.003833333
ND4	30	13.313	0.0001	0.003833333
ND4	206	15.159	0.0001	0.003833333
ND4	133	17.236	0.0001	0.003833333
ND4	90	17.362	0.0001	0.003833333
ND4	70	15.261	0.0001	0.003833333
ND4	346	13.246	0.0001	0.003833333
ND4	458	19.278	0.0001	0.003833333
ND4	374	10.615	0.001	0.032857143
ND4	259	11.525	0.001	0.032857143
COII	30	13.705	0.0001	0.0076
COII	7	18.272	0.0001	0.0076
COII	8	13.358	0.0001	0.0076
COIII	73	12.991	0.0001	0.0087
COIII	248	15.972	0.0001	0.0087
COIII	45	14.111	0.0001	0.0087
Cytb	267	17.104	0.0001	0.0048375
Cytb	244	16.935	0.0001	0.0048375
Cytb	338	19.276	0.0001	0.0048375
Cytb	25	19.536	0.0001	0.0048375
Cytb	372	23.477	0.0001	0.0048375
Cytb	15	15.138	0.0001	0.0048375
Cytb	374	14.293	0.0001	0.0048375
Cytb	10	19.556	0.0001	0.0048375
Cytb	170	10.406	0.001	0.0387
Cytb	308	10.406	0.001	0.0387

Supplementary Figure 1. Alignment of Oxford Nanopore 2D read with Illumina assembly of the Lady Alice Island Molecule 1 mt-Genome (LAI M1). Alignment of the Oxford Nanopore 2D (both strand connected) read covering the complete mitochondrial genome (Supplementary Data 1, Sequence Read Archive PRJNA445603; depicted above as Mt-Molecule 2D) and Illumina Assembly of the LAI M1 mt-genome (GenBank MN864228; depicted below as Illumina Draft). Sequences are presented as light strand sequence from 3' to 5'. The Oxford Nanopore 2D read was extracted as a consensus read with over hangs. The following sequences were clipped off the ends and the connection in *ND1* is noted by NN for two missing G bases when compared to the Illumina assembly: The first part of *ND1* cut sequence of 13 bases is 3'-CAATACGTAACCA-5' and the second part of *ND1* cut sequence of 17 bases is 3'-GCATAAACAGAACGTTA-5'. At the bottom of the alignment is a line of identity (asterisk) and difference where: (1) O = Oxford Nanopore gap, (2) I = Illumina Assembly gap which is an extra imposed base in the Oxford Nanopore read, (3) R = A or G, (4) Y = C or T, (5) M = A or C, (6) W = A or T, (7) S = G or C, and (8) K = G or T. The two NN connecting the circular molecule are included in the base numbering but actually are either gaps or mis-read bases. Alignments were done with ClustalW v1.4 in MacVector v8.0 with parameters of an open gap penalty of one and extended gap penalty of two with transitions weighted. Two alignment comparative-lines were manually adjusted to favor deletions and insertions over base substitutional changes in the Oxford Nanopore read as noted at the end of each adjusted individual line with a dash A. *Note:* The Oxford Nanopore 2D read has 1192 gaps and the LAI M1 Illumina assembly has 60 gaps. There are 191 transitional differences between the Oxford Nanopore read and the Illumina assembly, which are distributed as 49 R positions and 142 Y positions. There are 41 transversional differences between the Oxford Nanopore read and the Illumina assembly, which are distributed as 7 K positions, 20 W positions, 2 S positions, and 12 M positions. In total there are 1484 differences producing a 92% identity between the Oxford Nanopore 2D read covering the entire molecule and the Illumina assembly of Lady Alice Island molecule 1.

```
Mt-Molecule 2D      1 GTCATCGTAGCTTAAAATATTAAAGCCTGGCCTTGAAGATGCCATTATAG    50
Illumina Draft      1 GTCATCGTAGCTTAAAATATTAAAGCCTGGCCTTGAAGATGCCATTATAG    50
                      **************************************************

Mt-Molecule 2D     51 GACTTGGTGCCTCAATGACGCAAAGATTTGGTCATAAAC--ACTATTACT    98
Illumina Draft     51 GACTTGGTGCCTCAATGACGCAAAGATTTGGTCATAAACTTACTATTACT   100
                      ***************************************OO*********

Mt-Molecule 2D     99 TAACTCT-AAATTACA--TGCAAGTATCAGCACGC-AATTAAGTTTCCTG   144
Illumina Draft    101 TAACTCTTAAATTACACATGCAAGTATCAGCACACCAGTGAAAATGCCC-   149
                      *******O********OO***************R*O*R*K**RW*K**YI

Mt-Molecule 2D    145 CCCAAACAACCACCAAAG-AGCCGGCATCAGGCACACCAAAGTGGCCAAA   193
Illumina Draft    150 TTAAAACAACCACCAAAGGAGCCGGCATCAGGCACACCAAAGTGGCCAAA   199
                      YYM***************O*******************************

Mt-Molecule 2D    194 GACGCCTTGCCTCGCCACACCCCCAAGG-TACACAGCAGTGATTAACATT   242
Illumina Draft    200 GACGCCTTGCCTCGCCACACCCCCAAGGGTACACAGCAGTGATTAACATT   249
                      ****************************O*********************

Mt-Molecule 2D    243 GCTTCATAAGTGAAAACTTGACTTAGCTATAGAGACCACGGCCAGTCAAT   292
Illumina Draft    250 AAGCCATAAGTGAAAACTTGACTTAGCTATAGAGACCACGGCCGGTCAAT   299
                      RMKY***************************************R******

Mt-Molecule 2D    293 TTCGTGCCAGCCACCGCGGTTAAACGAATTT------GAAAGTAAAAGCC   336
Illumina Draft    300 TTCGTGCCAGCCACCGCGGTTAAACGAATTTTAGGCCGAAAGTAAAAGCC   349
                      *******************************OOOOOO*************

Mt-Molecule 2D    337 CAACGGCGTAAAAGTAA-TAAATCACCACCC-TTCACTAAAACCCAAGAA   384
Illumina Draft    350 CAACGGCGTAAAAGTAACTAAATCACCACCCCTTCACTAAA-CCCAAGAA   398
                      *****************O*************O*********I********

Mt-Molecule 2D    385 AAA-CTAAGATGAGTTGTAAAAA-CGCAC--CTATAAAA-CCTCTACATG   429
Illumina Draft    399 AAAACTAAGATGAGTTGTAAAAAACGCACACCTATAAAAACCTCTACATG   448
                      ***O*******************O*****OO********O**********

Mt-Molecule 2D    430 AAGATGTCTTGGGGACAAGAATAAAATTGAACTTACTAAAATTAAGG-AC   478
Illumina Draft    449 AAGATGTCTTGGGGACAAGAATAAAATTGAACTTACTAAAATTAAGGGAC   498
                      ***********************************************O**

Mt-Molecule 2D    479 AA-CTGG-ATTAGATACCCCACTATGCTTAATCCTAAACATCGACACCTA   526
Illumina Draft    499 AAACTGGGATTAGATACCCCACTATGCTTAATCCTAAACATCGACACCTA   548
                      **O****O******************************************

Mt-Molecule 2D    527 AACATA-A--GGTGTTCGCCCGGGAACTACCAGCAAA-CGCTAGAAACC-   571
Illumina Draft    549 AACATACAAGGGTGTTCGCCCGGGAACTACCAGCAAAACGCTAGAAACCC   598
                      ******O*OO***************************O***********O

Mt-Molecule 2D    572 TAAAGACTTGACGGTGCCCCAAAACCCC-TAGAGGAGCCTGTTC-ATAAT   619
Illumina Draft    599 TAAAGACTTGACGGTGCCCCAAAACCCCCTAGAGGAGCCTGTTCTATAAT   648
                      ****************************O***************O*****

Mt-Molecule 2D    620 TGATGATCCGCA-CAA-CCTCACCATTTTTCGCCCC-AGCCTATATGCTA   666
Illumina Draft    649 TGATGATCCGCAATAAACCTCACCATTTTTCGCCCCCAGCCTATAT---A   695
                      ************OY**O*******************O*********III*

Mt-Molecule 2D    667 CCGCCGTCGCCAGTCTACCTTGTGAAAGAATTAAGTAGGTCAAACAGCAT   716
Illumina Draft    696 CCGCCGTCGCCAGTCTACCTTGTGAAAGAATTAAGTAGGTCAAACAGCAT   745
                      **************************************************

Mt-Molecule 2D    717 TACCGCTAATACGTCAGGTCAAGGTGCAGCCAATAAAATGGAAAGAA-TG   765
Illumina Draft    746 TACCGCTAATACGTCAGGTCAAGGTGCAGCCAATAAAATGGAAAGAAATG   795
                      ***********************************************O**
```

```
Mt-Molecule 2D    766 GGCTACATTCCCACAAAACCGGGAAATACTAAAAGTTAAATGAAAA--TA   813
Illumina Draft    796 GGCTACATTCCCACAAAACCGGGAAATACTAAAAGTTAAATGAAAAAATA   845
                      **********************************************OO**

Mt-Molecule 2D    814 ACAAGAAGGTGGATTTAGTAG--------AACCATAATATCAAGTTGAAA   855
Illumina Draft    846 ACAAGAAGGTGGATTTAGTAGTAAACTAGAACCATAATATCAAGTTGAAA   895
                      *********************OOOOOOOO*********************

Mt-Molecule 2D    856 A-GGCTCTGGAGCGCGTACACACCGCCCGTCACCCTCCTATTAA-CCAAA   903
Illumina Draft    896 AAGGCTCTGGAGCGCGTACACACCGCCCGTCACCCTCCTATTAAACCAAA   945
                      *O******************************************O*****

Mt-Molecule 2D    904 -GGAGGCAAAGTCGTAACACGGTAAGCATACTGGAAAGTGTGCTTG-AAA   951
Illumina Draft    946 AGGAGGCAA-GTCGTAACACGGTAAGCATACTGGAAAGTGTGCTTGGAAA   994
                      O********I************************************O***

Mt-Molecule 2D    952 CAAAATGTAGCTTATTATAA-GCCCCTCAGCCTACACCTGAACAATGTTAG  1001-A
Illumina Draft    995 CAAAATGTAGCTTATTATAAAGCCC-TCAGCCTACACCTGAACAATGTTAG  1044-A
                      ********************O*****I*************************

Mt-Molecule 2D   1002 CACACCTAACCATTT-GAACCTTAAACTAGCCCGCTACC-AGATAATCTA  1049
Illumina Draft   1045 CACACCTAACCATTTTGAACCTTAAACTAGCCCGCTACCCAGATAATCTA  1094
                      ***************O***********************O**********

Mt-Molecule 2D   1050 AAACCAATTCACTAACCCAAAACATTTTTCGAGCTAAGTATAG-AGATAG  1098
Illumina Draft   1095 AAACCAATTCACTAACCCAAAACATTTTTCGAGCTAAGTATAGGAGATAG  1144
                      *******************************************O******

Mt-Molecule 2D   1099 AAAAGAACC--AGGAGTTATAGAGACAGTACCGCAAGG-AAAGTTTAAAA  1145
Illumina Draft   1145 AAAAGAACCCCAGGAGCTATAGAGACAGTACCGCAAGGGAAAGTTTAAAA  1194
                      *********OO*****Y*********************O***********

Mt-Molecule 2D   1146 TAC-AAGTTAAATAAAGCAAAGATAGACCCTTGTACCTTTTGAATAATGG  1194
Illumina Draft   1195 TACCAAGTTAAATAAAGCAAAGATAGACCCTTGTACCTTTTGAATAATGG  1244
                      ***O**********************************************

Mt-Molecule 2D   1195 TTTAACTAGTCCAAACAAGCTAAAAGAGCTAAGCTTTT-ACCCCGAAATC  1243
Illumina Draft   1245 TTTAACTAGTCCAAACAAGCTAAAAGAGCTAAGCTTTTTACCCCGAAATC  1294
                      **************************************O***********

Mt-Molecule 2D   1244 AAGTGAGCTACTTATAAGCGTACAGAAATAATTCGGCTCTGTGGCAAA-G  1292
Illumina Draft   1295 AAGTGAGCTACTTATAAGCGTACAGAAATAATTCGGCTCTGTGGCAAAAG  1344
                      ************************************************O*

Mt-Molecule 2D   1293 AGCGAAA-GACTAATAAGTAGAGGCGAAACACC----GAACATGAAGATA  1337
Illumina Draft   1345 AGCGAAAAGACTAATAAGTAGAGGCGAAACACCTACCGAACATGAAGATA  1394
                      *******O*************************OOOO*************

Mt-Molecule 2D   1338 GCTGGTTACTTATTAAACGAATC-AAGTTCTACCTTAACTCACCCAACC-  1385
Illumina Draft   1395 GCTGGTTACTTATTAAACGAATCTAAGTTCTACCTTAACTCACCCAACCC  1444
                      ***********************O*************************O

Mt-Molecule 2D   1386 TCCTTAAACAA-GAGAAATGAAAGATTTAAGAGATATTTAATAGGGGTAC  1434
Illumina Draft   1445 TCCTTAAACAAAGAGAAATGAAAGATTTAAGAGATATTTAATAGGGGTAC  1494
                      ***********O**************************************

Mt-Molecule 2D   1435 AGCCCTGGCCAAAATAGGAAACAGCCTATACAAGAGGAAAGA--ATTACA  1482
Illumina Draft   1495 AGCCCTA-TTAAAATAGGAAACAGCCTATACAAGAGGAAAGAGAATTACA  1543
                      ******RIYY********************************OO******

Mt-Molecule 2D   1483 CCAGACCGTAGGCCTTAAAGCAGCTACCAAA-TATAAAGCGTTAAAGCTA  1531
Illumina Draft   1544 CCAGACCGTAGGCCTTAAAGCAGCCACCAAAATATAAAGCGTTAAAGCTA  1593
                      ************************Y******O******************
```

```
Mt-Molecule 2D   1532 ATTAACACCAACACCAACCTTTTTAATCCAACCCTAAACAA---TAAGTC  1578
Illumina Draft   1594 ATTAACACCAACACCAACCTTTTTAATCCAACCCTAAACAACAATAAGTC  1643
                      *****************************************OOO******

Mt-Molecule 2D   1579 ATACTATAATAATATAACAGAATTAATGTTAAAATGAGTAACAAGAAA--  1626
Illumina Draft   1644 ATACTATAATAATATAACAGAATTAATGTTAAAATGAGTAACAAGAAAAA  1693
                      ************************************************OO

Mt-Molecule 2D   1627 GATTTC--CGT--ATCCGCATAAGCCAGACTAGACAACCTACTGGTCATT  1672
Illumina Draft   1694 GATTTCTCCGTGCATCCGCATAAGCCAGACTAGACAACCTACTGGTCATT  1743
                      ******OO***OO*************************************

Mt-Molecule 2D   1673 GCACAATAATTAATA---CACAACGAGCAATAATATTAATCTAACTGTTA  1719
Illumina Draft   1744 ACACAATAATTAATATTACACAACGAGCAATAATATTAATCTAACTGTTA  1793
                      R**************OOO********************************

Mt-Molecule 2D   1720 ACCCAACACAGGCATGCAATCTAG-AAAGATTAAACTCACTAGTAG-AAC  1767
Illumina Draft   1794 ACCCAACACAGGCATGCA-CCTAGGAAAGATTAAACTCACCAGAAGGAAC  1842
                      ******************IY****O***************Y**W**O***

Mt-Molecule 2D   1768 TCGGCAACACCGGGCCCTGACTGTTTACCAAAA-CATAACCTT-AGCTAA  1815
Illumina Draft   1843 TCGGCAACACCGGGCCCCGACTGTTTACCAAAAACATAACCTTTAGCTAA  1892
                      *****************Y***************O*********O******

Mt-Molecule 2D   1816 TCA-GTATTAAAGGCGCCTCCTGCCCAGGAAGAAAATTAAACGGCCGCGG  1864
Illumina Draft   1893 TCAAGTATTAAAGGCGCCTCCTGCCCAG--TGAAAATTAAACGGCCGCGG  1940
                      ***O************************OOW*******************

Mt-Molecule 2D   1865 TATTTCAAC-GTGCAA-GGTAGCGTAATCAATTGTCTC-TAAATGAAGAC  1911
Illumina Draft   1941 TATTCTGACCGTGCAAAGGTAGCGTAATCAATTGTCTCCTAAATGAAGAC  1990
                      ****YYR**O******O*********************O***********

Mt-Molecule 2D   1912 TAGTATGAACGGAGAAACGAGG-CCCAACTGTCTC-TGGCGAAAATCAGT  1959
Illumina Draft   1991 TAGTATGAACGGAGAAACGAGGGCCCAACTGTCTCCTGGCGAAAATCAGT  2040
                      **********************O************O**************

Mt-Molecule 2D   1960 GAAATTGATCC--TTGTGCAAA-GCAAGGATAAA-CCATAAGACGATAAG  2005
Illumina Draft   2041 GAAATTGATCCCCTTGTGCAAAAGCAAGGATAAAACCATAAGACGATAAG  2090
                      ***********OO*********O***********O***************

Mt-Molecule 2D   2006 ACC--GTGGAGCT-AAACATCAGAGTCAATTATACATGTTGAATC-ACCA  2051
Illumina Draft   2091 ACCCCGTGGAGCTTAAACATCAGAGTCAATTATACATGTTGAATCCACCA  2140
                      ***OO********O*******************************O****

Mt-Molecule 2D   2052 GGAATAAAATCAAAAA-TGACCCC-TCTGTTTTG-CTGGG-CGGCCTCG-  2096
Illumina Draft   2141 GGAATAAAATCAAAAAATGACCCCCTCTGTTTTGGCTGGGGCGGCCTCGG  2190
                      ****************O*******O*********O*****O********O

Mt-Molecule 2D   2097 AATAAAAA----CTTCCGAAAAC-TCATTTAGACCAACAAGTCAAA-CAT  2140
Illumina Draft   2191 AATAAAAAAAAACTTCCGAAAACCTCATTTAGACCAACAAGTCAAAACAT  2240
                      ********OOOO***********O**********************O***

Mt-Molecule 2D   2141 AAAATACTTGACCCAATATTATTGATTATTGAACCAAGTTACCCCAGGGA  2190
Illumina Draft   2241 AAAATACTTGACCCAATATTATTGATTATTGAACCAAGTTACCCCAGGGA  2290
                      **************************************************

Mt-Molecule 2D   2191 TAACAGCGCCATCCC-TTCGAGAGCCCATATCGACAAGG-AGATTACGAC  2238
Illumina Draft   2291 TAACAGCGCCATCCCCTTCGAGAGCCCATATCGACAAGGGAGATTACGAC  2340
                      ***************O***********************O**********

Mt-Molecule 2D   2239 CTCGATGTTGGATCAGGACATCCTAGTGGTGCAACCGCTACTAAGGGTTC  2288
Illumina Draft   2341 CTCGATGTTGGATCAGGACATCCTAGTGGTGCAACCGCTACTAAGGGTTC  2390
                      **************************************************
```

```
Mt-Molecule 2D   2289 GTTTGTTCAACGATTAACAGTCCTACGTGATCTGAGTTCAGACCGGAGCA  2338
Illumina Draft   2391 GTTTGTTCAACGATTAACAGTCCTACGTGATCTGAGTTCAGACCGGAGCA  2440
                      **************************************************

Mt-Molecule 2D   2339 ATCCAGGTCGGTTTCTATCTATGACATACTATTTC-AGTACGAAAGGATC  2387
Illumina Draft   2441 ATCCAGGTCGGTTTCTATCTATGACATACTATTTCCAGTACGAAAGGATC  2490
                      ***********************************O**************

Mt-Molecule 2D   2388 GAAACAGTAGGGCATAACCGCAAGCAAGCC-TGCCCTTAATTAATGAAAC  2436
Illumina Draft   2491 GAAACAGTAGGGCATAACCGCAAGCAAGCCCTGCCCTTAATTAATGAAAC  2540
                      ******************************O*******************

Mt-Molecule 2D   2437 AAAATAAATTAA----CTGGGAT-TCCCC-ACGCCCTAAACCAGGGCTGC  2480
Illumina Draft   2541 AAAATAAATTAATTAACTGGGATCTCCCCCACGCCCTAAACCAGGGCTGC  2590
                      ************OOOO*******O*****O********************

Mt-Molecule 2D   2481 TGGCGTAGCAAAACCTGGATATGCTAAAGACTTAAGCACTTTTACACAGA  2530
Illumina Draft   2591 TGGCGTAGCAAAACCTGGATATGCTAAAGACTTAAGCACTTTTACACAGA  2640
                      **************************************************

Mt-Molecule 2D   2531 GGTTCAACTCCTCTCGCCAACAATGCTATCTCTATTAACCTGACTGATAG  2580
Illumina Draft   2641 GGTTCAACTCCTCTCGCCAACAATGCTATCTCTATTAACCTGACTGATAG  2690
                      **************************************************

Mt-Molecule 2D   2581 ACCCACTTACATATACATCATCCCAATCCTAATTGCAGTAGCATTCTTAA  2630
Illumina Draft   2691 ACCCACTT--ATATACATCATCCCAATCCTAATTGCAGTAGCATTCTTAA  2738
                      ********OO****************************************

Mt-Molecule 2D   2631 CATTACT-GAACGAAAA-TTCTAGGATATATACAATTACGAAAAGGCCCA  2678
Illumina Draft   2739 CATTACTAGAACGAAAAATTCTAGGATATATACAATTACGAAAAGGCCCA  2788
                      *******O*********O********************************

Mt-Molecule 2D   2679 AATATAGTAGGCCCAAAGGG-ATTCTACAAC-TATTGCAGACGGCCTAAA  2726
Illumina Draft   2789 AATATAGTAGGCCCAAAGGGGATTCTACAACCTATTGCAGACGGCCTAAA  2838
                      ********************O**********O******************

Mt-Molecule 2D   2727 ACTATTTATAAA-GAACCAGTACGTCCATATGCCGCCTCCCAAGCACTAT  2775
Illumina Draft   2839 ACTATTTATAAAAGAACCAGTACGTCCATATGCCGCCTCCCAAGCACTAT  2888
                      ************O*************************************

Mt-Molecule 2D   2776 TCATTATGGCCCCAATCTTAGCGCTATCATTATCCATTATATTATGATCA  2825
Illumina Draft   2889 TCATTATGGCCCCAATCTTAGCGCTATCATTATCCATTATATTATGATCA  2938
                      **************************************************

Mt-Molecule 2D   2826 CCTCTTTCAATACCATTTC----AATGGACATAAACCTCGGCCTGTTAAT  2871
Illumina Draft   2939 CCTCTTTCAATACCATTTCCTTTAATGGACATAAACCTCGGCCTGTTAAT  2988
                      *******************OOOO***************************

Mt-Molecule 2D   2872 CATACTCGCACTTTCAAGCATAGCAGTATATACAATCTTATGATCGGGGC  2921
Illumina Draft   2989 CATACTCGCACTTTCAAGCATAGCAGTATATACAATCTTATGATCGGG-C  3037
                      ************************************************I*

Mt-Molecule 2D   2922 TGATC-TCAAATT--AAATACGCTCTTA-AGNNGCTATTCGAGCGGTAGC  2967
Illumina Draft   3038 TGATCCTCAAATTCTAAATACGCTCTTATAGGGGCCATTCGAGCGGTAGC  3087
                      *****O*******OO************O**NN**Y**************

Mt-Molecule 2D   2968 A--AACTATCTCTTATGAAGTCACACTAG-AATTATTATTT-ATCTACAA  3013
Illumina Draft   3088 ACAAACTATCTCTTATGAAGTCACACTAGGAATTATTATTTTATCTACAA  3137
                      *OO**************************O***********O********

Mt-Molecule 2D   3014 TCTTACTAAGCG-AGGATTTA--ATTCAAACCCTAAACGCCACGCAAGAA  3060
Illumina Draft   3138 TCTTACTAAGCGGAGGATTTACAATTCAAACCCTAAACGCCACGCAAGAA  3187
                      ************O********OO***************************
```

```
Mt-Molecule 2D   3061 CCAATATGACTCTTAATAACATCATGACCAATAATA-T--TATGGTTAAC  3107
Illumina Draft   3188 CCAATATGACTCTTAATAACATCATGACCAATAATAATAATATGGTTAAC  3237
                      ************************************O*OO**********

Mt-Molecule 2D   3108 ATCTACCCTTTGCAGAAACAAACCGA-CCCCATTTGACCTAACAG--GGT  3154
Illumina Draft   3238 ATCTACCCTT-GCAGAAACAAACCGAGCCCCATTTGACCTAACAGAGGGT  3286
                      **********I***************O******************OO***

Mt-Molecule 2D   3155 GAGTCTGAACTTGTCTCAGGATTTAATGTAGAATATTCAGCAGGCCCATT  3204
Illumina Draft   3287 GAGTCTGAACTTGTCTCAGGATTTAATGTAGAATATTCAGCAGGCCCATT  3336
                      **************************************************

Mt-Molecule 2D   3205 TGCTTTATTCTTCCTAGCAGA-T-CGCCAATATCATAATAATAAATACAG  3252
Illumina Draft   3337 TGCTTTATTCTTCCTAGCAGAATACGCCAATATCATAATAATAAATACAG  3386
                      *********************O*O**************************

Mt-Molecule 2D   3253 TATCATGCATTATATTTATAAATCCAGGGCAAGGAATAATACCAGAACTA  3302
Illumina Draft   3387 TATCATGCATTATATTTATAAATCCAGGGCAAGGAATAATACCAGAACTA  3436
                      **************************************************

Mt-Molecule 2D   3303 TTCACCATAAATATTATAATAAAAACAACTATCCTAACAATTTGATTCCT  3352
Illumina Draft   3437 TTCACCATAAATATTATAATAAAAACAACTATCCTAACAATTTGATTCCT  3486
                      **************************************************

Mt-Molecule 2D   3353 ATGGGTCCGGGCCTCCTACC-TCGATTCCGATATGACCAAC-C---CATC  3397
Illumina Draft   3487 ATGGGTCCGGGCCTCCTACCCTCGATTCCGATATGACCAACTCATGCATC  3536
                      ********************O********************O*OOO****

Mt-Molecule 2D   3398 TTCTATGAAAAA-CTTCCTCCCAATC-TCCTAGCATTATGCCTTTGACAC  3445
Illumina Draft   3537 TTCTATGAAAAAACTTCCTCCCAATCACCCTAGCATTATGCCTTTGACAC  3586
                      ************O*************OY**********************

Mt-Molecule 2D   3446 ACATCTATGCCTATTACCTTATCAGGGATTCTC--AGCAATTTGGACATG  3493
Illumina Draft   3587 ACATCCATGCCTATTACCTTATCAGGGATTCCCCCAGCAATTTGGACATG  3636
                      *****Y*************************Y*OO***************

Mt-Molecule 2D   3494 TGCCTGAATAAAGGATCACTTTGATAGAGTGAACTAAAA-GGTTTAAACC  3542
Illumina Draft   3637 TGCCTGAATAAAGGATCACTTTGATAGAGTGAACTAAAAAGGTTTAAACC  3686
                      ***************************************O**********

Mt-Molecule 2D   3543 TCTTTCATCTCCTAGAAAA-TGGGAATCGAACCCAAACATTAGAGACCAA  3591
Illumina Draft   3687 -CTTTCATCTCCTAGAAAAATGGGAATCGAACCCAAACATTAGAGACCAA  3735
                      I******************O******************************

Mt-Molecule 2D   3592 AACTCTATGTACCCCATCATACTGTTTTCTAAAGTAAAGTCAGCTAATAA  3641
Illumina Draft   3736 AACTCTATGTACCCCATCATACTGTTTTCTAAAGTAAAGTCAGCTAATAA  3785
                      **************************************************

Mt-Molecule 2D   3642 AGCTTCTGGGCTCATGCCCCAAAAATGTTGGTTAAACC-TTCCTT-ACTA  3689
Illumina Draft   3786 AGCTTCTGGGCCCATGCCCCAAAAATGTTGGTTAAACCCTTCCTTTACTA  3835
                      ***********Y**************************O******O****

Mt-Molecule 2D   3690 ATGCACC--TCCATCACCTCTTTGCTCCTTATCGCCCTATCCACA-GCAC  3736
Illumina Draft   3836 ATGCACCCCTCCATCACCTCTTTGCTCCTTATCGCCCTATCCACAAGCAC  3885
                      *******OO************************************O****

Mt-Molecule 2D   3737 TATTAT--------CAAGCTACCATTGAATATTCGCCTGAGCTG-ATTG-  3776
Illumina Draft   3886 TATTATTACAATATCAAGCTACCATTGAATATTCGCCTGAGCTGGATTGG  3935
                      ******OOOOOOOO******************************O****O

Mt-Molecule 2D   3777 AAAT--ATA------CTATACTACCC-TAATCTCAAA-GAACACCACCCC  3816
Illumina Draft   3936 AAATTAATATACTCTCTATACTACCCCTAATCTCAAAAGAACACCACCCC  3985
                      ****OO***OOOOOO***********O**********O************
```

```
Mt-Molecule 2D    3817 CGAGCTGCGTAGAAGCCACCATAAA-TATTTCATGACA--AGCCGCCGCC  3863
Illumina Draft    3986 CGAGC--CGTAGAAGCCACCATAAAATATTTCATGACACAAGCCGCCGCC  4033
                       *****II*****************O***********OO*********

Mt-Molecule 2D    3864 TCAGCCATATTAAT--TCGCAAGCACCATTAATGCTTGACAAACTGCCCA  3911
Illumina Draft    4034 TCAGCCATATTAATCTTCGCAAGCACCATTAATGCTTGACAAACTGCCCA  4083
                       **************OO**********************************

Mt-Molecule 2D    3912 ATGAGACATTTCATTACTCACACCCTCAACACCAATAATAATATTAACAC  3961
Illumina Draft    4084 ATGAGACATTTCATTACTCACACCCTCAACACCAATAATAATATTAACAC  4133
                       **************************************************

Mt-Molecule 2D    3962 TAGCTATTACCATAAAACTAGGCTTA---CCATTCCACTTCTGATTGCCA  4008
Illumina Draft    4134 TAGCTATTACCATAAAACTAGGCTTGGCCCCATTCCACTTCTGATTGCCA  4183
                       *************************ROOO*********************

Mt-Molecule 2D    4009 GAAGTATCATTAG-AACAACCAA----GT---ATTAATTATCACAACATG  4050
Illumina Draft    4184 GAAGTATCATTAGGAACAACCAACTCAGTTATATTAATTATCACAACATG  4233
                       *************O*********OOOO**OOO******************

Mt-Molecule 2D    4051 ACAAAAACTGGCCCCACTAGCCATCATTATTCTATTGCACAACTCATTAA  4100
Illumina Draft    4234 ACAAAAACTGGCCCCACTAGCCATCATTATTCTATTGCACAACTCATTAA  4283
                       **************************************************

Mt-Molecule 2D    4101 ATCATCACTTGTTGACCAGC-TCGGT--TTTATCTATGGTAGTTGGGGG-  4146
Illumina Draft    4284 ATCATCACTTGTTGACCAGCCTCGGTCTTTTATCTATGGTAGTTGGGGGG  4333
                       ********************O*****OO*********************O

Mt-Molecule 2D    4147 TGAGGA-------ACCA-CCATAAATTCGAAAAAT-ATAGCATAC--CTC  4185
Illumina Draft    4334 TGAGGAGGAATGAACCAATTACAAATTCGAAAAATTATAGCATACTCCTC  4383
                       ******OOOOOOO****OYY*Y*************O*********OO***

Mt-Molecule 2D    4186 AATCGCTC-TATAG-ATG----GTTATA-TTTTAACAAA-GCCCCAGACC  4227
Illumina Draft    4384 AATCGCTCATATAGGATGAGTAGTTATAATTTTACCAAAAGCCCCAGACC  4433
                       ********O*****O***OOOO******O*****M****O**********

Mt-Molecule 2D    4228 TATCCCTACTGTACCTTATACTATATATTATGTTAAAC-TTACAATATTC  4276
Illumina Draft    4434 TATCCCTACTGTACCTTATACTATATATTATGTTAAACCTTACAATATTC  4483
                       **************************************O***********

Mt-Molecule 2D    4277 TTAGTTATT---TCCACCAC--TAACCAAGCTGACAAGCCTTTTT-CAAC  4320
Illumina Draft    4484 TTAGTTATTATTTCCACCACCCTAACCAAGCTGACAAGCCTTTTTTCAAC  4533
                       *********OOO********OO***********************O****

Mt-Molecule 2D    4321 ACAAA-CAAATCATTCCC-ATAATAATCTTGATAGCCCTAACC-TGCTTT  4367
Illumina Draft    4534 ACAAAACAAATCATTCCCCATAATAATCTTGATAGCCCTAACCCTGCTTT  4583
                       *****O************O************************O******

Mt-Molecule 2D    4368 CAATAGGAGGCCTACCACCCATAACAGGGTTTT-ACCAAA-TGATTAATT  4415
Illumina Draft    4584 CAATAGGAGGCCTACCACCCATAACAGGGTTTTTACCAAAATGATTAATT  4633
                       *********************************O******O*********

Mt-Molecule 2D    4416 CTACAAGAACTAATCTACCAACACCAA---CAAATGGCCACTCTAGCATC  4462
Illumina Draft    4634 CTACAAGAACTAATCTACCAACACCAAACACAAATGGCCACTCTAGCATC  4683
                       ***************************OOO********************

Mt-Molecule 2D    4463 CTT---CACCTTGCTCAGC-TCTT----TACCTCCGC-TAGTATTCAACG  4503
Illumina Draft    4684 CTTATCCACCTTGCTCAGCCTCTTCTTTTACCTCCGCCTAGTATTCAACG  4733
                       ***OOO*************O****OOOO*********O************

Mt-Molecule 2D    4504 CATTTCTAACCA-TC-ACCAAACACCTCAAATGTGACAAAAA-CTGATGA  4550
Illumina Draft    4734 CATTTCTAACCACTCCACCAAACACCTCAAATGTGACAAAAAACTGACGA  4783
                       ************O**O**************************O****Y**
```

```
Mt-Molecule 2D   4551 AAC-AAACCAACCACTACATTTTACTCCCACAACTAATA-T-TTGTCCC-  4596
Illumina Draft   4784 AACCAAACCAACCACTACATTTTACTCCCACAACTAATAATCTTGTCCTT  4833
                      ***O***********************************O*O******YO

Mt-Molecule 2D   4597 AATATTACTTATTA---CCCCACTAGTTATTAGCCTATTTTAAGAAATTT  4643
Illumina Draft   4834 AATATTACTTATTATTACCCCACTAGTTATTAGCCTATTTTAAGAAATTT  4883
                      **************OOO*********************************

Mt-Molecule 2D   4644 AGGATATA--AACCAGGG-CCTTCAAGCCC-AAAAA---GACACCTC--  4684
Illumina Draft   4884 AGGATATATAAACCAGGGGCCTTCAAGCCCCAAAAAAGAGACACCTCTT  4933
                      ********OO********O***********O*****OOO********OO

Mt-Molecule 2D   4685 -ATTTCTGAGGAAAC-TGAAAGATTCTTACTTTCATAGCCTTAATGCA-C  4731
Illumina Draft   4934 AATTTCTGAGGAAACCTGAAAGATTCTTACTTTCATAGCCTTAATGCAAC  4983
                      O**************O********************************O*

Mt-Molecule 2D   4732 CAAGACACTT--ATTAAGATAAGGCTTCCTAGGATGAGCAGGCCTCGATC  4779
Illumina Draft   4984 TAAGACACTTTTATTAAGATAAGGCTTCCTAG-ATGAGCAGGCCTCGATC  5032
                      Y*********OO********************I*****************

Mt-Molecule 2D   4780 CTACAAACTCT-AGTTAACAGCTAAACACCT-AAACAACAGGCTTCCATC  4827
Illumina Draft   5033 CTACAAACTCTTAGTTAACAGCTAAACACCTTAAACAACAGGCTTCCATC  5082
                      ***********O*******************O******************

Mt-Molecule 2D   4828 TACTAAGCCTG-ACTATATTTCTAATCTTTC-TAAGTTTGCAGCTCAGTA  4875
Illumina Draft   5083 TACTAAGCCTGGACTATATTTCTAATCTTTCCTAAGTTTGCAGCTCAGTA  5132
                      ***********O*******************O******************

Mt-Molecule 2D   4876 CTTGACAGGCCTGATAAGAAAAG-AATTAAAC-TTTATAAATAGGTCTAC  4923
Illumina Draft   5133 CTTGACAGGCCTGATAAGAAAAGGAATTAAACCTTTATAAATAGGTCTAC  5182
                      ***********************O********O*****************

Mt-Molecule 2D   4924 AGCCTAACACTCAACACT-GGCCATCTTACCTT-GCCATACAAACGATGA  4971
Illumina Draft   5183 AGCCTAACACCTAACACTCGGCCATCTTACCTTTGCCATACAAACGATGA  5232
                      **********YY******O**************O****************

Mt-Molecule 2D   4972 ---TACTCAACCAACCGGTTAT-CATTGGCACCCTAT--TTACTATTTGG  5015
Illumina Draft   5233 CTCTACTCAACCAACCACAAAGACATTGGCACCCTATATTTACTATTTGG  5282
                      OOO*************RSWW*WO**************OO***********

Mt-Molecule 2D   5016 GGCCTGAGCAGGAATAGTAGGAACCTCC-TAAGTCTCCTAATCCGAGGAG  5064
Illumina Draft   5283 GGCCTGAGCAGGAATAGTAGGAACCTCCCTAAGTCTCCTAATCCGAGGAG  5332
                      ****************************O*********************

Mt-Molecule 2D   5065 AATT--GTTATCCAG-AACACTTATAGG-AACGACCAAATCTACAACGTA  5110
Illumina Draft   5333 AATTAAGCTATCCAGGAACACTTATAGGGAACGACCAAATCTACAACGTA  5382
                      ****OO*Y*******O************O*********************

Mt-Molecule 2D   5111 ATCGTAATA--CCATGCTTTCACCATAATTTTT---ATAGTAATACCAGT  5155
Illumina Draft   5383 ATCGTAACAGCCCATGCTTTCACCATAATTTTTTTTATAGTAATACCAGT  5432
                      *******Y*OO**********************OOO**************

Mt-Molecule 2D   5156 AATAATCG-AGGATT-GGAA-CCGACTAATTCCATTAATAATTG-AGCCC  5201
Illumina Draft   5433 AATAATCGGAGGATTTGGAAACTGACTAATTCCATTAATAATTGGAGCCC  5482
                      ********O******O****O*Y*********************O*****

Mt-Molecule 2D   5202 CAGACATGGCCTTCCCC-GAATAAACAACATAAGCTTTTGACTC-TCCCA  5249
Illumina Draft   5483 CAGACATGGCCTTCCCCCGAATAAACAACATAAGCTTTTGACTCCTCCCA  5532
                      *****************O**************************O*****

Mt-Molecule 2D   5250 ---TCATTCCTTT----ATTGA--TCCGCCTGAACAGAAACTG-AGCAGG  5289
Illumina Draft   5533 CCATCATTCCTTTTACTATTGACATCCGCCTGAACAGAAACTGGAGCAGG  5582
                      OOO**********OOOO*****OO*******************O******
```

```
Mt-Molecule 2D    5290 GACAG-ATGAACTGTA--CCTGCCTCTAGCAG-AAACCTGGCTCA-----  5330
Illumina Draft    5583 GACAGGATGAACCGTATACCCGCCTCTAGCAGGAAACCTGGCCCACGCAG  5632
                       *****O******Y***OO**Y**********O*********Y**OOOOO

Mt-Molecule 2D    5331 GACC---CGCAGAC-TAACTATTTTT--CCTACACTTG-CTGGCGTCT--  5371
Illumina Draft    5633 GACCAAGCGTAGACCTAACCATTTTTTCCCTACACTTGGCTGGCGTCTCT  5682
                       ****OOO**Y****O****Y******OO*********O*********OO

Mt-Molecule 2D    5372 TCAATCTTAGGAGCAATTAATCTCATCACCA--ATCA---ATATAAA--C  5414
Illumina Draft    5683 TCAATCTTAGGAGCAATTAACTTCATCACCACAATCATTAATATAAAACC  5732
                       ******************YY*********OO****OOO*******OO*

Mt-Molecule 2D    5415 CCCAAACCAAT---AATACCAAATACC-TTATTCATTTGATC-GTCCTAG  5459
Illumina Draft    5733 CCCAAACCAATCTCAATACCAAATACCCTTATTCATTTGATCCGTCCTAG  5782
                       ***********OOO************O**************O*******

Mt-Molecule 2D    5460 TCACAGCAGTATTACTACTCTTATCCTTACCCGTACTAGCAG----AATT  5505
Illumina Draft    5783 TCACAGCAGTATTACTACTCTTATCCTTACCCGTACTAGCAGCAGGAATT  5832
                       ******************************************OOOO****

Mt-Molecule 2D    5506 ACAATA------ACTGACCGAAACCTAAATACT--ATT--TTGACC-A-C  5543
Illumina Draft    5833 ACAATACTTCTAACTGACCGAAACCTAAATACTTCATTCTTTGACCCATC  5882
                       ******OOOOOO*******************OO***OO******O*O*

Mt-Molecule 2D    5544 TGGAGGAGGAGACC--ATCCTGTACCAA-ATTTATTTTGATTTT---GCC  5587
Illumina Draft    5883 TGGAGGAGGAGACCCCATCCTGTACCAACACTTATTTTGATTTTTTGGCC  5932
                       **************OO************O*Y*************OOO***

Mt-Molecule 2D    5588 ACC-AGAAGTTTATATCTTAATCTTACCTGGATTTGGGATAATC--ACAT  5634
Illumina Draft    5933 ACCCAGAAGTTTATATCTTAATCTTACCTGGATTTGGGATAATCTCACAT  5982
                       ***O****************************************OO****

Mt-Molecule 2D    5635 -----AACCGACTACTCAAACAAGAAAGAACCTTTCGGATATATAGGAAT  5679
Illumina Draft    5983 GTAGTAACCGACTACTCAAACAAGAAAGAACCTTTCGGATATATAGGAAT  6032
                       OOOOO*********************************************

Mt-Molecule 2D    5680 AGTCTGAGCAAT---TTCAATTGGATTCTAAGGCTC-ATTGTATGGGCCC  5725
Illumina Draft    6033 AGTCTGAGCAATAATTTCAATTGGATTCCTAGGCTTTATTGTATGGGCCC  6082
                       ************OOO************YW*****YO*************

Mt-Molecule 2D    5726 ACCACATATTCACAGTCG-AATGGACGTAGACAC--GCGCCTATTTTACA  5772
Illumina Draft    6083 ACCACATATTCACAGTCGGAATGGACGTAGACACACGCGCCTATTTTACA  6132
                       ******************O***************OO**************

Mt-Molecule 2D    5773 TCTGCAACTATAATTATTGCCATCCCAACAG-CATTAAAGTGTTT----G  5817
Illumina Draft    6133 TCTGCAACTATAATTATTGCCATCCCAACAGGCATTAAAGTGTTTAGCTG  6182
                       *******************************O*************OOOO*

Mt-Molecule 2D    5818 ACTAGCTACCCTTTATGGAG-AATAATTAAATGAGAAGCAAGCATGCTCT  5866
Illumina Draft    6183 ACTAGCTACCCTTTATGGAGGAATAATTAAATGAGAAGCAAGCATGCTCT  6232
                       ********************O*****************************

Mt-Molecule 2D    5867 GAGCCCTGGGTTTTATTTTCTTTATTTACCGTAGGTGGATTAACTG-AAT  5915
Illumina Draft    6233 GAGCCCTGGGTTTTATTTTCTT-ATTTACCGTAGGTGGATTAACTGGAAT  6281
                       **********************I***********************O***

Mt-Molecule 2D    5916 CATTCTAGCAA-CTCATCCTTAGACATTATT-TC-ACGACACTTATTAC-  5961
Illumina Draft    6282 CATTCTAGCAAACTCATCCTTAGACATTATTCTCCACGACACTTATTACG  6331
                       ***********O*******************O**O**************O

Mt-Molecule 2D    5962 -AGTAGCGCA--TC------GCAC-ATCTATAG-AGCAGTATT-GCAATC  5999
Illumina Draft    6332 TAGTAGCGCACTTCCACTACGTACTATCTATAGGAGCAGTATTTGCAATC  6381
                       O*********OO**OOOOOO*Y**O********O*********O******
```

```
Mt-Molecule 2D   6000 ATGGCTG-AC-AACCCATTGATTCCCCTCCCAATAACAGGATATATATTA  6047
Illumina Draft   6382 ATGGCTGGACTAACCCATTGATTCCCCCTCC--TAACAGGATATATATTA  6429
                      *******O**O***************YY**II*****************

Mt-Molecule 2D   6048 CACAAAACATGAGCAAAAGTACACTTCGCACTAATATTCATCGGAGTAAA  6097
Illumina Draft   6430 CACAAAACATGAGCAAAAGTACACTTCGCACTAACATTCATCGGAGTAAA  6479
                      **********************************Y***************

Mt-Molecule 2D   6098 C-TAACCTTTTTCCCACAACACTTCTTAGGCCTAGCAGGAATG-----AC  6141
Illumina Draft   6480 CCTAACCTTTTTCCCACAACACTTCTTAGGCCTAGCAGGAATGCCACGAC  6529
                      *O*****************************************OOOOO**

Mt-Molecule 2D   6142 GATACTCAGACTACCCAGATGCATACTCCACATGAAATACCCTATCATCT  6191
Illumina Draft   6530 GATACTCAGACTACCCAGATGCATACTCCACATGAAATACCCTATCATCT  6579
                      **************************************************

Mt-Molecule 2D   6192 TT--GATCACTTATCTCCTTAACAGCTATCAT---TATACTTTT-ATCTT  6235
Illumina Draft   6580 TTTGGATCACTTATCTCCTTAACAGCTATCATCATTATACTTTTTATCTT  6629
                      **OO****************************OOO*********O*****

Mt-Molecule 2D   6236 TTGAGAGGCTTTT----CAAAACGAGAAGTAGACCACA--AAAATAAACT  6279
Illumina Draft   6630 TTGAGAGGCTTTTTCTTCAAAACGAGAAGTAGACCACACAAAAATAAACT  6679
                      *************OOOO*********************OO**********

Mt-Molecule 2D   6280 -CACAGGCTCAGAATGATTACATG---ACTTC--------ACACACCA-C  6316
Illumina Draft   6680 TCACAGGCTCAGAATGATTACACGGACACCCCCCACAACTACACACCAAC  6729
                      O*********************Y*OOO**YY*OOOOOOOO********O*

Mt-Molecule 2D   6317 GAAGAACCC---TTTATTTTACCAACCACAAGAGA------AATTGAAAT  6357
Illumina Draft   6730 GAAGAACCCCCCTTTTATTT-ACCAACCACAAGAGAGGAAGGAATTGAACC  6778
                      *********OOO*******I**************OOOOOO*******MY

Mt-Molecule 2D   6358 T--TCCAAC-AGTTTCAAGCTAATTGAGCAACCACATACAATCTCTCTTA  6404
Illumina Draft   6779 CCCTCCAACTAGTTTCAAGCTAATTGAGCAACCACATACAATCTCTCTTA  6828
                      YOO******O****************************************

Mt-Molecule 2D   6405 GG-ACTTTAGTAAAA-TTA--CGTT-TTGTCATGAACGAGTTAA-GACTT  6448
Illumina Draft   6829 GGGACTTTAGTAAAAATTATACGTTCTTGTCATGAACGAGTTAAAGACTT  6878
                      **O************O***OO****O******************O*****

Mt-Molecule 2D   6449 AAACCCT---------GCATGGCCTACCCCATACAAATTGCACTACAAAA  6489
Illumina Draft   6879 AAACCCTTTAAGCCCCGCATGGCCTACCCCATACAAATTGCACTACAAAA  6928
                      *******OOOOOOOOO**********************************

Mt-Molecule 2D   6490 CGCATC-TCTCCTCTAATAGAAGAACTTC---------ACGACCATGCAC  6529
Illumina Draft   6929 CGCATCCTCTCCTCTAATAGAAGAACTTCTACACTTCCACGACCATGCAC  6978
                      ******O**********************OOOOOOOOO************

Mt-Molecule 2D   6530 TAATAGTCATCCTATTAATCAGCACATCCGTGTTCTACATAATCACTATA  6579
Illumina Draft   6979 TAATAGTCATCCTATTAATCAGCACATCCGTGTTCTACATAATCACCATA  7028
                      **********************************************Y***

Mt-Molecule 2D   6580 ACACTGACTACTAAACTAACACACACATTAACGCAACAGACGCCC-AATC  6628
Illumina Draft   7029 ACACTGACTACTAAACTAACACAC--ATTAACGCAACAGACGCCCCAATC  7076
                      ************************II*******************O****

Mt-Molecule 2D   6629 ACAGAAACAGTATGAGT-ATCCTACCATCTATTATTCTAGTAACAATTGC  6677
Illumina Draft   7077 ACAGAAACAGTATGAACCATCCTACCATCTATTATTCTAGTAACAATTGC  7126
                      ***************RYO********************************

Mt-Molecule 2D   6678 CCTACCAT---TACAAATCCTATACCTAATAGATGAAATCAACAACC--T  6722
Illumina Draft   7127 CCTACCATCATTACAAATCCTATACCTAATAGATGAAATCAACAACCCCT  7176
                      ********OOO************************************OO*
```

```
Mt-Molecule 2D   6723 TTCTAACCATCAAAGCCCTTGGCCACCAATGATACTGAAGTTATGAATAT  6772
Illumina Draft   7177 TTCTAACCATCAAAGCCCTTGGCCACCAATGATACTGAAGCTATGAATAT  7226
                      ****************************************Y*********

Mt-Molecule 2D   6773 AT-GACTACAAAGACCTCTCATTTGAC--CTACATAATCCCAACAAA-GA  6818
Illumina Draft   7227 ACAGACTACAAAGACCTCTCATTTGACTCCTACATAATCCTAACAAAAGA  7276
                      *YO***********************OO**********Y******O**

Mt-Molecule 2D   6819 ATCAAAA--TGGACACTTCCGCCTGCTAG---CAGACCACTGAATGATT-  6862
Illumina Draft   7277 ACTAAAAAACGGACACTTCCGCCTGCTAGAAGTAGACCACCGAATGATTG  7326
                      *YY****OOY******************OOOY*******Y********O

Mt-Molecule 2D   6863 -ACCAACAAAATCCCCAATCCGAATTTTGGTATCAG---AAG-CGTCCTA  6907
Illumina Draft   7327 TACCAACAAAATCCCCAATCCGAATTTTGGTATCAGCAGAAGATGTCCTA  7376
                      O**********************************OOO***OO******

Mt-Molecule 2D   6908 CACTCATGAGCCGTCCCATCACTAGGAGTAAAA-TCGACGCCGTACCAGG  6956
Illumina Draft   7377 CACTCATGAGCCGTCCCATCACTAGGAGTAAAAATCGACGCCGTACCAGG  7426
                      *********************************O****************

Mt-Molecule 2D   6957 ACGCCTAAATCAGTCCACAATCTCAACACACC-GTTGTACCTTT---ACG  7002
Illumina Draft   7427 ACGCCTAAATCAAACTACAATCTCAACACACCTGCCAGGCCTTTTTTACG  7476
                      ************RW*Y****************O*IIIII*****OOO***

Mt-Molecule 2D   7003 GCCAATGCTCAGAGATTTGCGGAGCCAACCATAGCTTTATACCAATTGTT  7052
Illumina Draft   7477 GCCAATGCTCAGAGATTTGCGGAGCCAACCATAGCTTTATACCAATTGTT  7526
                      **************************************************

Mt-Molecule 2D   7053 ATTGAAGCTGTACCTATCTCACAACACTTCGAAGACTGATCATGGGCCA-  7101
Illumina Draft   7527 ATTGAAGCTGTACCTATCTCACA---CTTCGAAGACTGATCATGGGCCAC  7573
                      ***********************III***********************O

Mt-Molecule 2D   7102 -ACCCA-CA-CCGAGAAA-TG-TACAGCACCAGCCCTTTAGCGGAGACAG  7146
Illumina Draft   7574 AACCCAATAACCGAGAAGCTAATGCAGCACCAGCCCTTTAGCGGAGACAG  7623
                      O*****OY*O*******RO*RO*R**************************

Mt-Molecule 2D   7147 -AACA--TTCTCT--TCGTTTGAACCGAGAAGC--ACGCAGCACCAGCCT  7189
Illumina Draft   7624 GAACACATTCTCTCTTCGTTTGAACCGAGAAGCTAATGCAGCACCAGCCT  7673
                      O****OO******OO*****************OO*Y*************

Mt-Molecule 2D   7190 T--AAGCTG-AGACAGGAACACATTAAT---TTCGTTAA--CC------A  7225
Illumina Draft   7674 TTTAAGCTGGAGACAGGAACACATTGTTCCCTTCGTTAATGCCCCAACTA  7723
                      *OO******O***************RW*OOO********OO**OOOOOO*

Mt-Molecule 2D   7226 AACCTG-CCCCATGATTATCAACAATAGTCCTAGTGTGGCTCTCACTTAG  7274
Illumina Draft   7724 AACCCGGCCCCCTGATTATCAACAATAGTCCTAGTGTGGCTCTCACTAGG  7773
                      ****Y*O****M*************************************WR*

Mt-Molecule 2D   7275 AGTAATACACCAAAA-GTGAAAC-----------ATTTATCCCACAACCA  7312
Illumina Draft   7774 AGTAATACACCAAAAAGTGAAACTATTCCAAAACATTTATCCCACAACCA  7823
                      ***************O*******OOOOOOOOOOO****************

Mt-Molecule 2D   7313 TAAAG--GTG-AGC---TC----ACCTTGACCT-GACTGCTATCC-GAACC  7351-A
Illumina Draft   7824 TGT-GCTGCGGAGCCCCTCGTCCACCTTGACCTTGACCATGATCCCGAACC  7873-A
                      *RWI*OO*Y*O***OOO**OOOO**********O***YRYK****O*****

Mt-Molecule 2D   7352 TTTT-GAAATTTTTGCCACTCCTTATTTACTAAAAATACCCCTTATATGA  7400
Illumina Draft   7874 TTTTTGAAATTTTTGCCACTCCTTATTTACTAAAAATACCCCTTATATGA  7923
                      ****O*********************************************

Mt-Molecule 2D   7401 CTAGCGGTGATAATACCAGCAATAGTTCTTTT-ACCCC-AGCCAACACCT  7448
Illumina Draft   7924 CTAGCGGTGATAATACCAGCAATAGTTCTTTTTACCCCCAGCCAACACCT  7973
                      ********************************O*****O***********
```

```
Mt-Molecule 2D    7449 TGTAACAGG-CGTTATATTCTACTTAAAAA-TGAACAATGAAGCAATTCA  7496
Illumina Draft    7974 TGTAACAGGGCGTTATATTCTACTTAAAAAATGAACAATGAAGCAATTCA  8023
                       *********O********************O*******************

Mt-Molecule 2D    7497 CTAAACAGATTATAAGCCTACTC--AACGCAAGGCCATAAATGAACGGTG  7544
Illumina Draft    8024 CTAAACAGATTATAAGCCCACTCCCAACGCAAGGCCATAAATGAACGGTG  8073
                       ******************Y****OO*************************

Mt-Molecule 2D    7545 TTTT-CATGACCTTAATAA---CC--AATATCAATAAACATGATTAGTCT  7588
Illumina Draft    8074 TTTTTCATGACCTTAATAATAACCCTAACATCAATAAACATGATTAGTCT  8123
                       ****O**************OOO**OO**Y*********************

Mt-Molecule 2D    7589 ACTCCCTCACACATTCACCCCAACAGCC-ACTTAGCATTAACCATGGCCC  7637
Illumina Draft    8124 ACTCCCTCACACATTCACCCCAACAGCCCAATTAGCATTAACCATGGCCC  8173
                       ****************************O*M*******************

Mt-Molecule 2D    7638 TAGCCTTTCCATTATGGTTAGCCACCGCA-TCATCGGGGTATTAAAAA--  7684
Illumina Draft    8174 TAGCCTTTCCATTATGGTTAGCCACCGTAATCATCGGGGTATTAAAAAAA  8223
                       ***************************Y*O******************OO

Mt-Molecule 2D    7685 -CAACTCGATCACTAGCACATTTCCTCCCAG----TTTC--ATTC-TTAT  7726
Illumina Draft    8224 ACAACTCGATCACTAGCACATTTCCTCCCAGAAGGCTCCCCAACCATTCT  8273
                       O******************************OOOOY*Y*OO*WY*O**M*

Mt-Molecule 2D    7727 -ATCCCAGCCTTC-TG-TAGTTGAAACAATCAGCCTCTTAATTCGCCC-T  7772
Illumina Draft    8274 TATCCCAGCCCTTGTGGTAGTTGAAACAATCAGCCTCTTAATTCGCCCCT  8323
                       O*********Y*YO**O*******************************O*

Mt-Molecule 2D    7773 TAGCACTCGGAGTACGCCTAGCAGCTAACCTAACAGCAGGACATCTACTA  7822
Illumina Draft    8324 TAGCACTCGGAGTACGCCTAGCAGCTAACCTAACAGCAGGACATCTACTA  8373
                       **************************************************

Mt-Molecule 2D    7823 CT--AACCAATAGCCCTAGCAATCATCCACCTTC-AACACC--TATGACT  7867
Illumina Draft    8374 CTTCAACTAATAGCCCTAGCAATCATCCACCTTCCAACACCCATATGAC-  8422
                       **OO***Y**************************O******OO******I

Mt-Molecule 2D    7868 TCGGCTACAGTGGCCGTAGCAACAACTTTAATACTA---ACCATCCTAGA  7914
Illumina Draft    8423 -CGGCTACAGTGGCCGTAGCAACAACTTTAATACTACTAACCATCCTAGA  8471
                       I***********************************OOO***********

Mt-Molecule 2D    7915 AGTAGCAGTAGCACTTATTCAAGCCTATGTATTTGTACTC--ACTATCCC  7962
Illumina Draft    8472 AGTAGCAGTAGCACTTATTCAAGCCTATGTATTTGTACTCCTACTATCCC  8521
                       ****************************************OO********

Mt-Molecule 2D    7963 TGTACCTACAAGAAA-CATCTAATGTCCACT-AACCCACACATACTACA  8010
Illumina Draft    8522 TGTACCTACAAGAAAACATCTAATGTCCACTCAAACCCACACATATCACA  8571
                       ***************O***************O************YY***

Mt-Molecule 2D    8011 TCGTAGAACAAA--CCATGACCTATTACAGGAGCAATATCTAC--TATTA  8056
Illumina Draft    8572 TCGTAGAACAAAGTCCATGACCTATTACAGGAGCAATATCTGCCCTATTA  8621
                       ************OO***************************R*OO*****

Mt-Molecule 2D    8057 TCAACATCAGGTCTCGCC-TAAGATT-----ATA-CTCTACGACATTAAT  8099
Illumina Draft    8622 ATAACATCAGGTCTCGCCGTATGATTTCACTATAACTCTACGACATTAAT  8671
                       WY****************O**W****OOOOO***O***************

Mt-Molecule 2D    8100 CGTTACCG-ATTACT--GCCTTCT---AACAATATACCAATGATGACGA-  8142
Illumina Draft    8672 CGTTACCGGATTACTAAGCCTTCTTCTAACAATATACCAATGATGACGAG  8721
                       ********O******OO*******OOO**********************O

Mt-Molecule 2D    8143 -TATTATACGAGAAG--AT-TTTCATG-ACACCAC--CCTATTTGTACAA  8185
Illumina Draft    8722 ATATTATACGAGAAGGCACATTTCAAGGACACCACACCCTATTTGTACAA  8771
                       O**************OO*YO*****W*O*******OO*************
```

```
Mt-Molecule 2D   8186 AA-GGGCTACGATATGGAATAATTTTGTTTATTGTCTCCGAGGTGTT---  8231
Illumina Draft   8772 AAAGGGCTACGATATGGAATAATTTTGTTTATTGTCTCCGAGGTGTTCTT  8821
                      **O********************************************OOO

Mt-Molecule 2D   8232 -TTTATTGGTTTCTTTTGA-CCTTTTATCACTCTAGCCTATCCCC-ACAC  8278
Illumina Draft   8822 TTTTATTGGTTTCTTTTGAGCCTTTTATCACTCTAGCCTATCCCCCACAC  8871
                      O******************O*************************O****

Mt-Molecule 2D   8279 CAGAAATTG-AGGCCAATA--CGCTAACTG---TTTTCCC---AGACC-A  8318
Illumina Draft   8872 CAGAAATTGGAGGCCAATGGCCGCCAACTGGGGTTTTCCCGTTAGACCCA  8921
                      *********O********ROO***Y*****OOO*******OOO*****O*

Mt-Molecule 2D   8319 TTCGAGGTCCCC-TTCTAAACACAACCGTATTATTAGCCTCCGGGGTAAC  8367
Illumina Draft   8922 TTCGAGGTCCCCCTTCTAAACACAACCGTATTATTAGCCTCCGGGGTAAC  8971
                      ************O*************************************

Mt-Molecule 2D   8368 AGTCACCTGAGCACATCATTCTATCATAGAAGG-AAACGAAA-TCGGCC-  8414
Illumina Draft   8972 AGTCACCTGAGCACATCATTCTATCATAGAAGGGAAACGAAAATCGGCCC  9021
                      *********************************O********O******O

Mt-Molecule 2D   8415 TTTTCT--TTATTGTTAACCATCACCCTAG-ATTATACTTCACAGCCTTA  8461
Illumina Draft   9022 TTTTCTCTTTATTGTTAACCATCACCCTAGGATTATACTTCACAGCCTTA  9071
                      ******OO**********************O*******************

Mt-Molecule 2D   8462 CAAGCAATAGAGTATTACGAAGCCC--TTTACAATTTCAGATAATGTT-A  8508
Illumina Draft   9072 CAAGCAATAGAGTATTACGAAGCCCCTTTTACAATTTCAGATAATGTTTA  9121
                      *************************OO*********************O*

Mt-Molecule 2D   8509 TG----AACCTTTT--GT----ACAGGTTTCCACGGACTC-ATGTAATTA  8547
Illumina Draft   9122 TGGAACAACCTTTTTTGTGGCCACAGGTTTCCACGGACTCCATGTAATTA  9171
                      **OOOO********OO**OOOO******************O*********

Mt-Molecule 2D   8548 TCGGC--CTCCTT-C--ACTGTATGCCTCCTACGACAAATCCTTCA----  8588
Illumina Draft   9172 TCGGCTCCTCCTTTCTAATTGTATGCCTCCTACGACAAATCCTTCATCAC  9221
                      *****OO******O*OO*Y***************************OOOO

Mt-Molecule 2D   8589 --------------CCATTTGGGCTTGAGGCAGCAGCATGATATTGACA  8624
Illumina Draft   9222 TTCACCATAGACCACCATTTGGGTTTGAGGCAGCAGCATGATATTGACA  9271
                      OOOOOOOOOOOOOO*********Y**************************

Mt-Molecule 2D   8625 TTTCGTAGACGTAGTATGGCTATTCC-ATTTACCTCAAT----TGATGAG  8669
Illumina Draft   9272 TTTCGTAGACGTAGTATGGCTATTCCTATTTACCTCAATCTACTGATGAG  9321
                      **************************O************OOOO*******

Mt-Molecule 2D   8670 G-ACCTATTCTTT-AGTATTATTAGTACAAATGACTTC-AATCATTAAGT  8716
Illumina Draft   9322 GGACCTATTCTTTTAGTATTATTAGTACAAATGACTTCCAATCATTAAGT  9371
                      *O***********O************************O***********

Mt-Molecule 2D   8717 TCTAGGCAACCTAGAAAAAGAATAATGAACATATTAATC-TGCTT--AGT  8763
Illumina Draft   9372 TCTAGGCAACCTAGAAAA-GAATAATGAACATATTAATCCTGCTTTTAGT  9420
                      ******************I********************O*****OO***

Mt-Molecule 2D   8764 TATAATAATCCTCTTATCTATAGCATTAATCACAATTAACATCTGACTCC  8813
Illumina Draft   9421 TATAATAATCCTCTTATCTATAGCATTAATCACAATTAACATCTGACTCC  9470
                      **************************************************

Mt-Molecule 2D   8814 CAAACACA--ACCACATCAAGAAAAACTATCACCATACGAATGCGGCTTT  8861
Illumina Draft   9471 CAAACACACAACCACATCAAGAAAAACTATCACCATACGAATGCGGCTTT  9520
                      ********OO****************************************

Mt-Molecule 2D   8862 GACTCCTTAATTTCCGCTC-----CCATT----ATGCGCTTT--CTTGGT  8900
Illumina Draft   9521 GACCCTTTAATCTCCGCTCGCCTCCCATTTCTATGCGCTTTTTCTTGGT  9570
                      ***Y*Y*****Y*******OOOOO*****OOOO*********OO******
```

```
Mt-Molecule 2D   8901 AGCCGTC-TATTC-TTTTATTTGATCTAGAAATTGCC----TCCTACCAC  8944
Illumina Draft   9571 AGCCGTCCTATTCCTTTTATTTGATCTAGAAATTGCCCTCCTCCTACCAC  9620
                      *******O*****O**********************OOOO*********

Mt-Molecule 2D   8945 TACCATGAGCTGTACAA-----CACC---ACCGCAACCATATGACATGAA  8986
Illumina Draft   9621 TACCATGAGCTGCACAATCTAACACCCCTACCGCAACCAT--GACATGAA  9668
                      ************Y****OOOOO****OOO**********II********

Mt-Molecule 2D   8987 CTTCC-TAATTTTAACC-TCCTAACAC-CGGATTAATATATGAATGAACT  9033
Illumina Draft   9669 CTTCCCTAATTTTAACCCTCCTAACACTCGGATTAATATATGAATGAACT  9718
                      *****O***********O*********O**********************

Mt-Molecule 2D   9034 CAAGGAG-ACTG-AGTGA-----ATGAAGAGTTAGTTCAATAAAGACA-C  9075
Illumina Draft   9719 CAAGGAGGACTGGAGTGAGCCGAATGAAGAGTTAGTTCAATAAAGACAAT  9768
                      *******O****O*****OOOOO***********************OY

Mt-Molecule 2D   9076 TAACTTCGAC--AATTAATCATGGTTTAATCCATGA-TCTTCAATGCCTA  9122
Illumina Draft   9769 TAACTTCGACTTAATTAATCATGGTTTAATCCATGACTCTTCAATGCCTA  9818
                      **********OO************************O*************

Mt-Molecule 2D   9123 CCATTTTATTTAATCTGGGCTTGGCTTTTATAACAAGTATAATAGGCCTG  9172
Illumina Draft   9819 CCATTTTATTTAATCTGGGCTTGGCTTTTATAACAAGTATAATAGGCCTG  9868
                      **************************************************

Mt-Molecule 2D   9173 TCATTACAACGCTCACACCT-ATCTCCGCTG-AT-ATGCT--GAAGGCAT  9217
Illumina Draft   9869 TCATTACAACGCTCACACCTTATCTCCGCTTTATTATGCTTGGAAGGCAT  9918
                      ********************O*********KO**O*****OO********

Mt-Molecule 2D   9218 AATATTAACTCTTTTTCTAATATTGA-------GGCCACAGAACAA---T  9257
Illumina Draft   9919 AATATTAACTCTTTTTCTAATATTAACAACCTGGGCCACAGAACAACAAT  9968
                      ************************R*OOOOOOO*************OOO*

Mt-Molecule 2D   9258 CTACCTTACTTTCTTTCTGCCTTA---------CTAACACT----GCTCG  9294
Illumina Draft   9969 CTACCTTACTCT-TTTCTGCCTTAATAATGCTACTAACACTTTCTGCCTG 10017
                      **********Y*I***********OOOOOOOOO********OOOO**YY*

Mt-Molecule 2D   9295 CGGAGCGGGCACAGGCCTGTCTTTACTTGTAACT------CGAACACACG  9338
Illumina Draft  10018 CGGAGCGGGCACAGGCCTGTCTTTACTTGTAACTACAACTCGAACACACG 10067
                      **********************************OOOOOO**********

Mt-Molecule 2D   9339 GATCAAGCAATATAAACAACCTAAATCTCCTTCAATGCTAAAA--TATTA  9386
Illumina Draft  10068 GATCAAGCAATATAAACAACCTAAATCTCCTTCAATGCTAAAAATTATTA 10117
                      *******************************************OO*****

Mt-Molecule 2D   9387 TTCCAACAATTTTCCTTCTCC-TGTAATC-TTTTGTCAAACCCCAAGTCA  9434
Illumina Draft  10118 TTCCAACAATTTTCCTTCTCCCTGTAATCCTTTTGTCAAACCCCAAGTCA 10167
                      *********************O*******O********************

Mt-Molecule 2D   9435 ACATGAATAAACTTC---ATTAATAGTCTACTAATCTCCGCCACTAGCTC  9481
Illumina Draft  10168 ACATGAATAAACTTCTTCATTAATAGTCTACTAATCTCCGCCACTAG-TC 10216
                      ***************OOO*****************************I**

Mt-Molecule 2D   9482 CAACTACCT--AAACCACAAATCAACATG-AGTGAAAGTT-TC-AGCCCT  9526
Illumina Draft  10217 TAACCATCTTAAAACCACAAATCAACACGGAGTGAAAGTTCTCTAGCCCT 10266
                      Y***Y*Y**OO****************Y*O**********O**O******

Mt-Molecule 2D   9527 TACACAG-ACTAGATCACATTTCATCCC-ACTATTAGTCCTAACAACATG  9574
Illumina Draft  10267 TACACAGGACTAGATCACATTTCATCCCCACTATTAGTCCTAACAACATG 10316
                      *******O********************O*********************

Mt-Molecule 2D   9575 ACTTCTGCCACTAATAACACTAGCAAGCCAAAACTCC-TCAAAAGC-AAC  9622
Illumina Draft  10317 ACTTCTGCCACTAATAACACTAGCAAGCCAAAACTCCCTCAAAAAAGAAC 10366
                      *************************************O******RMO***
```

```
Mt-Molecule 2D   9623 CATATGAGTGTAAACAACTATTCTTA-TTACCTTGACACTT--TCAGACC  9669
Illumina Draft  10367 CATATGAGCGTAAACAACTATTCTTAATTACCTTGACACTTCTTCAGACC 10416
                      ********Y*****************O**************OO*******

Mt-Molecule 2D   9670 TTCA-ATTAG-ATTTCTTTGTGCCACAGACCTG------TTTTATAT---  9708
Illumina Draft  10417 TTCCTACTAGGAGCCTTTTGTGCCACAGACCTGGCACTGTTTTATATCAT 10466
                      ***MO*Y***O*KYYY*****************OOOOOO********OOO

Mt-Molecule 2D   9709 --TCGAAGCAACG-TTATCCCTACACTAATCATTATC---TGATGAGGAA  9752
Illumina Draft  10467 ATTCGAAGCAACGCTTATCCCCACACTAATCATTATCACCCGATGAGGAA 10516
                      OO***********O*******Y***************OOOY*********

Mt-Molecule 2D   9753 ATCAACCAGAACGCCTTAATGCCG-AATATATTTCTTATTT-ACACACTC  9800
Illumina Draft  10517 ATCAACCAGAACGCCTTAATGCCGGAATATATTTCTTATTTTACACACTC 10566
                      ************************O****************O********

Mt-Molecule 2D   9801 ACAGCCTCTCTTCCATTACTAATTGCACTACTGACTTTATATAACCTGAA  9850
Illumina Draft  10567 ACAGCCTCTCTTCCATTACTAATTGCACTACTGACTTTATATAACCTGAA 10616
                      **************************************************

Mt-Molecule 2D   9851 CAACACCCTATTAATCACAATCCTACATTTCCTGCCCCAAAACACCAACC  9900
Illumina Draft  10617 CAACACCCTATTAATCACAATCCTACATTTCCTGCCCCAAAACACCAACC 10666
                      **************************************************

Mt-Molecule 2D   9901 CACCATCAACAAGTTTCTTTCTGTGAAGTGCATGTATAATAGCATTTTTA  9950
Illumina Draft  10667 CACCATCAACAAGTTTCTTTCTGTGAAGTGCATGTATAATAGCATTTTTA 10716
                      **************************************************

Mt-Molecule 2D   9951 GTAAAA-TACCATTATATAAG-TCCA--TATGACTACCAAA-GCCCATG-  9994
Illumina Draft  10717 GTAAAAATACCATTATATGGGCTCCACTTATGACTACCAAAAGCCCATGT 10766
                      ******O***********RR*O****OO*************O*******O

Mt-Molecule 2D   9995 --AAGC--CAATCGTTG-CTCAATGGTCT--GT-ACAATCCTCCTAAAAT 10036
Illumina Draft  10767 AGAAGCTCCAATCGCCGGCTCAATGGTCTTAGCCGCAATCCTCCTAAAAT 10816
                      OO****OO******YY*O***********OO*YOR***************

Mt-Molecule 2D  10037 TAG-AGGATATGGCATTATTCGTACCATCACCATACTCACCC-AATAACA 10084
Illumina Draft  10817 TAGGAGGATATGGCATTATTCGTACCATCACCATACTCACCCCAATAACA 10866
                      ***O**************************************O*******

Mt-Molecule 2D  10085 AAAACCATGTCAATCCCATTTATTACTTTATCTTTATGAGGG-TG-TTAT 10132
Illumina Draft  10867 AAAACCATGTCAATCCCATTTATTACTTTATCTTTATGAGGGGTGGTTAT 10916
                      ******************************************O**O****

Mt-Molecule 2D  10133 AATAAGCCTCATTTGCCTTCGACAAACAGATTTAAAATCT--TATCGCCT 10180
Illumina Draft  10917 AACAAGCCTCATTTGCCTTCGACAAACAGATTTAAAATCTCTTATCGCCT 10966
                      **Y*************************************OO********

Mt-Molecule 2D  10181 ATTCATCAGTAA---ATAAGGGCCTAGTAACAGCAGCCTCCCTAATCCAC 10227
Illumina Draft  10967 ATTCATCAGTAAGCCATATGGGCCTAGTAACAGCAGCCTCCCTAATCCAC 11016
                      ************OOO***W*******************************

Mt-Molecule 2D  10228 ACAGAATGAAGCCTAACGG-AGCCATCATTTCATTA-TAGCCCACGGCCT 10275
Illumina Draft  11017 ACAGAATGAAGCCTAACGGGAGCCATCATTTTAATAGTAGCCCACGGCCT 11066
                      *******************O***********Y*W**O*************

Mt-Molecule 2D  10276 TGTCTCCTCAGCACTATTTTTGTCTATCCAACTTTAATTATGAACG-GTT 10324
Illumina Draft  11067 TGTCTCCTCAGCACTATTTT-GTCTATCCAACTTTAATTATGAACGTATT 11115
                      ********************I*************************OR**

Mt-Molecule 2D  10325 AACAATCGAACACTACTTTTAGTACGAAGCATACAAC--ACTATACC--T 10370
Illumina Draft  11116 AACAATCGAACACTACTTTTAGTACGAAGCATACAACTAATTATACCCCT 11165
                      *************************************OO*Y******OO*
```

```
Mt-Molecule 2D  10371 TCTCTCCA--TGAT-ATTCCTGTTTAATCCCAT-AAACATAGCCCTCCCA 10416
Illumina Draft  11166 TCTCTCCACATGATGACTCCTGTTTAA-CCTATTAAACATAGCCCTCCCA 11214
                      ********OO****O*Y**********I**Y**O****************

Mt-Molecule 2D  10417 --ATTAATCAACCTAATAGGAGAACTACTAATTATTACGGCCAT--TCAA 10462
Illumina Draft  11215 CCAACAATCAACCTAATAGGAGAACTACTAATTATTACGGCCATATTCAA 11264
                      OO*WY**************************************OO****

Mt-Molecule 2D  10463 CT--TCT------ACAATCCT-ATTGCAGG--CTGGCGTAGTGTTAACAG 10501
Illumina Draft  11265 CTGATCCCCCCCCACAATCCTTATTACAGGAGCTGGCGTAGTGTTAACAG 11314
                      **OO**YOOOOOO********O***R****OO******************

Mt-Molecule 2D  10502 CCTGCTACTCATTACA--TATTCTTAACCACA--ACATAGCA-GTTCTCT 10546
Illumina Draft  11315 CCTGCTACTCATTACACATATTCTTAACCACACAACATAGCAAGTTCTCT 11364
                      ****************OO**************OO********O*******

Mt-Molecule 2D  10547 AGCCAC-TGGCATCCATACAA-TAACAAACACACGAGAACACCTATTAAT 10594
Illumina Draft  11365 AGCCACCTGGCATCCATACAAATAACAAACACACGAGAACACCTATTAAT 11414
                      ******O**************O****************************

Mt-Molecule 2D  10595 AAGCCTTCACATAATTCCAATAGTTCTC----TATTCAAACCAGAAC-AG 10639
Illumina Draft  11415 AAGCCTTCACATAATTCCAATAGTTCTCCTAATATTCAAACCAGAACTAG 11464
                      ****************************OOOO***************O**

Mt-Molecule 2D  10640 TATGAT-T--CA--TATTAACCAGATACC-TGACCG-TCACAAAGTGAAC 10682
Illumina Draft  11465 TATGATCTGGCACGTATTAACCAGATACCCTGACCGCTCGCAA-G-GAAC 11512
                      ******O*OO**OO***************O******O**R***I*I****

Mt-Molecule 2D  10683 CACGA--C--ACTT-GAGTCAATCCTAAA-CAACAAACAAAGCTACTAAC 10726
Illumina Draft  11513 CACGAGATTGACCCCGAGTCAACTCTAAAACAACAAACAAAGCTACTAAC 11562
                      *****OOYOO**YYO*******YY*****O********************

Mt-Molecule 2D  10727 AACACC-AACCAGAAACCACAAGCACAGAACACCCAAAATCATACAAAAT 10775
Illumina Draft  11563 AACACCCAACCAGAAACCACAAGCACAGAACACCCAAAATCATACAAAAT 11612
                      ******O*******************************************

Mt-Molecule 2D  10776 CGCAACCCC-ACAAAACTCTTCACGAATCAAAGAAATACCACCAAAATCA 10824
Illumina Draft  11613 CGCAACCCCCACAAAA-TCCCCACGAATCAAAGAAATACCACCAAAATCA 11661
                      *********O******I**YY*****************************

Mt-Molecule 2D  10825 ACTGTCTCTTCCCCAACCCC--TTTTACTTAACCCC-AAGAAAATCCAGA 10871
Illumina Draft  11662 ACTGTCTCTTCCCCAACCCCCTTTTTACTTAACCCCCAAGAAAATCCAGA 11711
                      ********************OO**************O*************

Mt-Molecule 2D  10872 CCCAACAAAAACTAGCAAAGATAAAAA--CCACAAGATTAACAGCAACTG 10919
Illumina Draft  11712 CCCAACAAAAACTAGCAAAGATAAAAAAACCACAAGATTAACAGCAACTG 11761
                      ***************************OO********************

Mt-Molecule 2D  10920 AACGACTCCCTCAAGCCTCAGGATAAAAATCAGCAGCCAAAGCAACAGAA 10969
Illumina Draft  11762 AACGACTCCCTCAAGCCTCAGGATAAAAATCAGCAGCCAAAGCAACAGAA 11811
                      **************************************************

Mt-Molecule 2D  10970 TAAGCGAACACTACTAACATACCACCCGAATAAATCAAAAACAAA-TGAC 11018
Illumina Draft  11812 TAAGCGAACACTACTAACATACCACCCGAATAAATCAAAAACAAAATGAC 11861
                      *********************************************O****

Mt-Molecule 2D  11019 AGACAAAAA-GAACTTACCAACACCGACCAAAAATCCCC-ACCAACA-T- 11064
Illumina Draft  11862 AGACAAAAAAGAACT-ACCAACACCGACCAAAAATCCCCCACCAACAGCA 11910
                      *********O*****I***********************O*******OYO

Mt-Molecule 2D  11065 GAAGCTATCAAA-GCCCTAACACCCCATAATAGGGCGAAGGGTTAGAAGC 11113
Illumina Draft  11911 GAAGCTATCAAAAGCCCTAACACCCCATAATAGGGCGAAGGGTTAGAAGC 11960
                      ************O*************************************
```

```
Mt-Molecule 2D  11114 CATTAACAA---ACCAAAGACGAAACAA-GACCAAACAGAAAA-TAGCAT 11158
Illumina Draft  11961 CACTAACAACAAACCAAAGACGAAACAAAGACCAAACAGAAAAATAGCAT 12010
                      **Y******OOO****************O**************O******

Mt-Molecule 2D  11159 AAGTCATTATTCTCACCAAGACTCAAACTCGGAATAGCGG-TTGAAAA-C 11206
Illumina Draft  12011 AAGTCATTATTCTCACCAAGACTCAAACTCGGAATAGCGGCTTGAAAAAC 12060
                      ****************************************O*******O*

Mt-Molecule 2D  11207 CGCCGTTATGTTTAACTATAAAA-TGCAAGGCGGCACGCC---TTATATT 11252
Illumina Draft  12061 CGCCGTTATGTTTAACTATAAAAATGCA-GGCGGCACGCCGCCTTATATT 12109
                      ***********************O****I***********OOO*******

Mt-Molecule 2D  11253 TATATATACTATACTCTAGTACGTCCTTCCCCTC-CTATGTATATATCGT 11301
Illumina Draft  12110 TATATATACTATACTCTAGTACGTCCTTCCCCCCTCCCTATGTATATCGT 12159
                      ********************************Y*O*YM*R*R********

Mt-Molecule 2D  11302 GCATTCCTTCTCGACCTCACGA-TAATCATACTCCATTGACTTCCTATTG 11350
Illumina Draft  12160 GCATTCCTTCTCGACCTCACGAGTAATCATACTCCATTGACTTCCTATTG 12209
                      **********************O***************************

Mt-Molecule 2D  11351 ATTCTACATATTAGTGAGA--TAAGCAACCCTTGAATGTAAGATTAATCA 11398
Illumina Draft  12210 ATTCTACATATTAGTGAGAGATAAGCAACCCTTGAATGTAAGATTAATCA 12259
                      *******************OO*****************************

Mt-Molecule 2D  11399 TTACTAGCTTCACGCGCATAATATCCTATTCCACATACTTT-CTTTTCAA 11447
Illumina Draft  12260 TTACTAGCTTCACGCGCATAATATCCTATTCCACATACTTTTCTTTTCAA 12309
                      *****************************************O********

Mt-Molecule 2D  11448 A-TACCTCTGGTTACTCTTTCCAGTGCTTACGGATCTTCC-TCCCGCAAC 11495
Illumina Draft  12310 AATACCTCTGGTTACTCTTTCCAGTGCTTACGGATCTTCCCTCCCGCAAC 12359
                      *O**************************************O*********

Mt-Molecule 2D  11496 T--TGATATTAAGAATACCTCTGGTTGCACTCGAGATTTAAAGACACGTT 11543
Illumina Draft  12360 TCTTGATATTAAGAATACCTCTGGTTGCACTCGAGATTTAAAGACACGTT 12409
                      *OO***********************************************

Mt-Molecule 2D  11544 CTGTCACAAATAAGTAACTTATAGTCTGCAAAACATTTGGTACTTTT--A 11591
Illumina Draft  12410 CTGTCACAAATAAGTAACTTATAGTCTGCAAAACATTTGGTACTTTTTTA 12459
                      ***********************************************OO*

Mt-Molecule 2D  11592 AAATATTTACCTTTCAACCA--TATCCAGTTATGTCTATACCATCCTATA 11639
Illumina Draft  12460 AAATATTTACCTTTCAACCACATATCCAGTTATGTCTATACCATCCTGTA 12509
                      ********************OO*************************R**

Mt-Molecule 2D  11640 TAGG-TCGGGCATATTAATGCATGGTGT--AACGGACATA----ATTCCA 11682
Illumina Draft  12510 TAGGGTCGGGCATATTAATGCATGGTGTGTAACGGACATAGACAATTCCA 12559
                      ****O***********************OO**********OOOO******

Mt-Molecule 2D  11683 AGAGGCTATTTAATTAATGCTTGTTAGACATAATACTACATATATTTCCC 11732
Illumina Draft  12560 AGAGGCTATTTAATTAATGCTTGTTAGACATAATACTACATATATTTCCC 12609
                      **************************************************

Mt-Molecule 2D  11733 C-TTCTTAACAAAAATTTT-ACCATTAACTTTAAAATATTTTATTATAAC 11780
Illumina Draft  12610 CCTTCTTAACAAAAATTTTTACCATTAACTTTAAAATATTTTATTATAAC 12659
                      *O*****************O******************************

Mt-Molecule 2D  11781 TTAGTTTTT-C-ACTAAACCCCC-TTACCCCC-ACGAACATAGATTAGCT 11826
Illumina Draft  12660 TTAGTTTTTTCTGCTAAACCCCCCTTACCCCCCACGAACATAGATTAGCT 12709
                      *********O*OR**********O********O*****************

Mt-Molecule 2D  11827 AACTTTATTCTTGTTAAACCCCTAAACCAAGAGTCAACTAAACTGAGTTT 11876
Illumina Draft  12710 AACTTTATTCTTGTTAAACCCCTAAACCAAGAGTCAACTAAACTGAGTTT 12759
                      **************************************************
```

```
Mt-Molecule 2D  11877 GTTCGTAG-AAAACTTTTT--CCCCCACTTTTT---AAAATGATAAATAA 11920
Illumina Draft  12760 GTTCGTAGGAAAACTTTTTTCCCCCCACTTTTTTTTAAAATGATAAATAA 12809
                      ********O**********OO************OOO**************

Mt-Molecule 2D  11921 TTTATAAAATCCTATACTAAGGAAGCCTTATGTATTTTTAAATTTAAACC 11970
Illumina Draft  12810 TTTATAAAATCCTATACTAAGGAAGCCTTATGTATTTTTAAATTTAAACC 12859
                      **************************************************

Mt-Molecule 2D  11971 -TAATTTT-AATGTATTTTT---ATCCTGCCAGGGCCACGGAACTTCAT 12015
Illumina Draft  12860 CTAATTTTTTAATGTATTTTTTTTATCCTGCCAGGGCCACGGAACTTCAC 12909
                      O********O***********OOO*************************Y

Mt-Molecule 2D  12016 -CAT---GAGGG-AGGAACCCCACCGG-TTTAGGAGTTGGCCATTCTTG- 12058
Illumina Draft  12910 GCTTTTAGAGGGGAGGAACCCCACCGGCTTTAGGAGCCGGCCATTCTTGG 12959
                      O*W*OOO*****O**************O********YY***********O

Mt-Molecule 2D  12059 CCAAACCCAAGTG-AAGCTATGATCGAAACCCAATGAATCACACCAATTA 12107
Illumina Draft  12960 CCAAACCCAAGTGGAAGCTATGATCGAAACCCAATGAATCACACCAATTA 13009
                      *************O************************************

Mt-Molecule 2D  12108 ACACAATAATGCTTGCATCCATTGGCATCCTTCTC-ACC--TTAATGGCC 12154
Illumina Draft  13010 ACACAATAATGCTTGCATCCATTAGCATCCTTCTCTACCCTTTAATGGCC 13059
                      ***********************R***********O***OO*********

Mt-Molecule 2D  12155 GATACCCTATTTAAAACTTCAATAAAGATACAAGCCCGTACTGCA---AA 12201
Illumina Draft  13060 GATACCCTATTTAAAACTTCAATAAAGATACAAGCCCGTACTGCAGTAAA 13109
                      *********************************************OOO**

Mt-Molecule 2D  12202 GTACGCCTTCTTTTCCACACTTGCCACC-TTATCATATTTATTAACTTAG 12250
Illumina Draft  13110 GTACGCCTTCTTTTCCACACTTGCCACCCTTATCATATTTATTAACTTAG 13159
                      ****************************O*********************

Mt-Molecule 2D  12251 -AA-GGAAATCAACACCACGCACATCAACTTAATCTCTTCCACAAATTTT 12298
Illumina Draft  13160 GAATAGAAATCAACACCACGCACATCAACTTAATCTCTTCCACAAATTTT 13209
                      O**OR*********************************************

Mt-Molecule 2D  12299 AACGTCGGTCCAAGCTTTAAATTCGATTACTTCACACTCATATTCTGCCC 12348
Illumina Draft  13210 AACGTCGGCCTAAGCTTTAAATTCGATTACTTCACACTCATATTCTGCCC 13259
                      ********Y*Y***************************************

Mt-Molecule 2D  12349 AACAGCACTATTTGTAACATGGTCTATTATAGATTTCGCCAAATGATATA 12398
Illumina Draft  13260 AACAGCACTATTTGTAACATGGTCTATTATAGATTTCGCCAAATGATATA 13309
                      **************************************************

Mt-Molecule 2D  12399 TAGCCCACGACCAAGAAGTCAATAAATTCTTTAAATACCTATTAATCTTT 12448
Illumina Draft  13310 TAGCCCACGACCAAGAAGTCAATAAATTCTTTAAATACCTATTAATCTTT 13359
                      **************************************************

Mt-Molecule 2D  12449 CTTTT-GTAATATTAACACTAGTCTCCTCAAACAACCTTTT-CAATTGTT 12496
Illumina Draft  13360 CTTTTAGCAATATTAACACTAGTCTCCTCAAACAACCTTTTTCAATTGTT 13409
                      *****O*Y*********************************O********

Mt-Molecule 2D  12497 TATCGGATGGGAAGGTGTTGGCATCATGTCTTTCCTACTAATCGGATGAT 12546
Illumina Draft  13410 TATCGGATGGGAAGGTGTTGGCATCATGTCTTTCCTACTAATCGGATGAT 13459
                      **************************************************

Mt-Molecule 2D  12547 GACGGACCTGAGGAGAA--TA---CAGC-TCAATACAAGCCATCATCTAC 12590
Illumina Draft  13460 GACGGACCCGAGGAGAAGCTAACACAGCCTCAATACAAGCCATCATCTAC 13509
                      ********Y********OO**OOO****O*********************

Mt-Molecule 2D  12591 AATCGCCTTG-AGACATTGGCTTTATTT-AACACTAGTATGAACTGGAAC 12638
Illumina Draft  13510 AATCGCCTTGGAGACATTGGCTTTATTTTAACACTAGCATGAACTGGAAC 13559
                      **********O*****************O********Y************
```

```
Mt-Molecule 2D  12639 CAACGTTGCTTCATGAGAACTCGACCAATTATTTATACTAAA-CCATTCG 12687
Illumina Draft  13560 CAACGTTGCTTCATGAGAACTCGACCAATTATTTATACTAAAACCATTCG 13609
                      ******************************************O*******

Mt-Molecule 2D  12688 ACACACTACCC-TTTTAGCCCTGGTCCTAGCAGCCGCTGCAAA-TCAGCA 12735
Illumina Draft  13610 ACACACTACCCCTTTTAGCCCTGGTCCTAGCAGCCGCTGCAAAATCAGCA 13659
                      ***********O*******************************O******

Mt-Molecule 2D  12736 CAATTTGGCATACACCCTTGACTATTAAA---TATAGAAGG-CCAACACC 12781
Illumina Draft  13660 CAATTTGGCATACACCCTTGACTATTAGGGGCCATAGAAGGGCCAACACC 13709
                      ***************************RROOOY********O********

Mt-Molecule 2D  12782 AGTATCAGCCCTACTTCACTCTAGCACAATAGTCGTAGCCGGCATTTTCT 12831
Illumina Draft  13710 AGTATCAGCCCTACTTCACTCTAGCACAATAGTCGTAGCCGGCATTTTCT 13759
                      **************************************************

Mt-Molecule 2D  12832 TATTAATCCGCCTTCACCCAATAATA-AAGACAACGAACTTACACGAACC 12880
Illumina Draft  13760 TATTAATCCGCCTTCACCCAATAATAGAAGACAACGAACTTACACGAACC 13809
                      **************************O***********************

Mt-Molecule 2D  12881 ACCTGCCTCTTACTTGGGGCCATCTCCACCTTTATAACCGCAATATGTGC 12930
Illumina Draft  13810 ACCTGCCTCTTACTTGGGGCCATCTCCACCTTTATAACCGCAATATGTGC 13859
                      **************************************************

Mt-Molecule 2D  12931 ACTAACACAAA-TGATATCAAAAA--TTATTGCTT-ATCAACAGC-GGTC 12975
Illumina Draft  13860 ACTAACACAAAATGATATCAAAAAAATTATTGCTTTATCAACAGCTAGTC 13909
                      ***********O************OO*********O*********OR***

Mt-Molecule 2D  12976 AACTAGGCCTAATAATAACAACCATCA-CCTTAATCAACCAAAC-TTGCC 13023
Illumina Draft  13910 AACTAGGCCTAATAATAACAACCATCGGCCTTAATCAACCAAACCTTGCC 13959
                      **************************RO****************O*****

Mt-Molecule 2D  13024 TTTTCTCCACATATGCCTACACGCATTCTTTTAAATCAAAACTATTTAT 13073
Illumina Draft  13960 TTT-CTCCACATATGCCTACACGCATTCTTT--AAATCAAAACTATTTAT 14006
                      ***I***************************II*****************

Mt-Molecule 2D  13074 TTGCTC-GGAATCATCTCCCACAACCTTAGCAACGAACAGGACATTCGAA 13122
Illumina Draft  14007 TTGCTCCGGAATCATCTCCCACAACCTTAGCAACGAACAGGACATTCGAA 14056
                      ******O*******************************************

Mt-Molecule 2D  13123 AA-TAGGAG-AATC-ACAAAACAATACCAATTACATCCTCCTGCTTCACA 13169
Illumina Draft  14057 AAATAGGAGGAATCCACAAAACAATACCAATTACATCCTCCTGCTTCACA 14106
                      **O******O****O***********************************

Mt-Molecule 2D  13170 ATTGGGAACCTTGCTCTAGCAGGAATTCCATTCATGACCGGCTTCTACTC 13219
Illumina Draft  14107 ATTGGGAACCTTGCTCTAGCAGGAATTCCATTCATGACCGGCTTCTACTC 14156
                      **************************************************

Mt-Molecule 2D  13220 AAAAGATGCTATCATTGAAACCATAAACTCATCTACACTAAACCTAG-AG 13268
Illumina Draft  14157 AAAAGATGCTATCATTGAAACCATAAACTCATCTACACTAAACCTAGGAG 14206
                      ***********************************************O**

Mt-Molecule 2D  13269 CACTTCTTCTTACCATGGCCGCCACCGTAATAACAGCTGCCTATACAACA 13318
Illumina Draft  14207 CACTTCTTCTTACCATGGCCGCCACCGTAATAACAGCTGCCTATACAACA 14256
                      **************************************************

Mt-Molecule 2D  13319 CGCCTTATCTTTT-AGTACAAACAGGCCCCC-ACGACATTACCCAATACA 13366
Illumina Draft  14257 CGCCTTATCTTTTTAGTACAAACAGGCCCCCCACGACATTACCCAATACA 14306
                      *************O*****************O******************

Mt-Molecule 2D  13367 AAAA-TCACTGA-GATAAAACC-TATCTAATGCCATCCTACGCCTCTCCT 13413
Illumina Draft  14307 AAAAATCACTGAAGATAAAACCCTATCTAATGCCATCCTACGCCTCTCCT 14356
                      ****O*******O*********O***************************
```

```
Mt-Molecule 2D  13414 TAGGAAGCATTGCAATTGGGCCTATTTCTGCTAAGTGACTTTAGACCAAA 13463
Illumina Draft  14357 TAGGAAGCATTGCAATTGGGCCTATTTCTGCTA--TGACTTTAGACCAAA 14404
                      *********************************II***************

Mt-Molecule 2D  13464 CTCAGACC-TCC-TACAACTGTCACCACCCTCATCAAACTCTCAGCACTA 13511
Illumina Draft  14405 CTCAGACCCTCCCTACAACTGTCACCACCCTCATCAAACTCTCAGCACTA 14454
                      ********O***O*************************************

Mt-Molecule 2D  13512 GGG-CCACCA--ATGG-AATCTTTGTATCACTTATTTTCATCCATAAAA- 13556
Illumina Draft  14455 GGGGCCACCATAATGGGAATCTTTGTATCACTTATTTTCATCCATAAAAA 14504
                      ***O******OO****O********************************O

Mt-Molecule 2D  13557 CAAACAAAACCGTCCACAAAAA--TAATTTGTCAACCTTTTCAAACATAT 13604
Illumina Draft  14505 CAAACAAAACCGTCCACAAAAAAATAACTTGTCAACCTTTTCAAACATAT 14554
                      **********************OO***Y**********************

Mt-Molecule 2D  13605 TAACATTCTATAGCTCCC------ACCGATCCCCCACAACCCACCTTT-- 13646
Illumina Draft  14555 TAACATTCTATAGCTCCCTAACCCACCGATCCCCCACAACCCACCTTTTA 14604
                      ******************OOOOOO************************OO

Mt-Molecule 2D  13647 -AAAAAGCACAAAA-GTATCCACAACC-TCAATGATGCAAC-TGATACGA 13692
Illumina Draft  14605 AAAAAAGCACAAAAAGTATCCACAACCCTCAATGATGCAACCTGATACGA 14654
                      O*************O************O*************O********

Mt-Molecule 2D  13693 ATATTCAGTCCC-AAATTTCTTCTCCAAAACACAAT-AAAGATATCAAAA 13740
Illumina Draft  14655 ATATTCAGTCCCCAAATT-CTTCTCCAAAACACAATTAAAGATATCAAAA 14703
                      ************O*****I*****************O*************

Mt-Molecule 2D  13741 GCCCTTTCCTTC-TGGCCCAGGGCAA-GTAAAA-CCTACTTAGCAGTATT 13787
Illumina Draft  14704 GCCCTTTCCTTCCTGGCCCAGGGCAAAGTAAAAACCTACTTAGCAGTATT 14753
                      ************O*************O******O****************

Mt-Molecule 2D  13788 CACAATATCCTTGATAACC-TGATTATCTTAACAAATCAGACCTCCCCAC 13836
Illumina Draft  14754 CACAATATCCTTGATAACCCTGATTATCTTAACAACCTAGACCTCCCCAC 14803
                      *******************O***************MYY************

Mt-Molecule 2D  13837 GCTGAACTTTTATAGCT-ATTAAAGCACCAACCTTGTAAATTG-AGACTG 13884
Illumina Draft  14804 GCTGAACTTTTATAGCTTATTAAAGCACCAACCTTGTAAATTGGAGACTG 14853
                      *****************O*************************O******

Mt-Molecule 2D  13885 AAGACTTATCCCTTCTAAAAGTATTTATAAAAACACTACACCTACTTAGT 13934
Illumina Draft  14854 AAGACTTATCCCTTCTAAAAGTATTTATAAAAACACTACACCTACTTAGT 14903
                      **************************************************

Mt-Molecule 2D  13935 TTAACAAAAACATCAGATTGTGATTCTGTCAATAGGGGCTAAGCCCCTTT 13984
Illumina Draft  14904 TTAACAAAAACATCAGACTGTGATTCTGTCAATAGGGGCTAAGCCCCTTT 14953
                      *****************Y********************************

Mt-Molecule 2D  13985 -AGGTACAGGCGGCATGCCGCCTTATATTTATATA--CTATACTCTAGTA 14031
Illumina Draft  14954 TAGGTACAGGCGGCATGCCGCCTTATATTTATATATACTATACTCTAGTA 15003
                      O**********************************OO*************

Mt-Molecule 2D  14032 CGTCCTTCCCCC-TCC-TATGTATATCGTGCATTCCTTCTCGACCTCACG 14079
Illumina Draft  15004 CGTCCTTCCCCCCTCCCTATGTATATCGTGCATTCCTTCTCGACCTCACG 15053
                      ************O***O*********************************

Mt-Molecule 2D  14080 AGTAATCATACTCCATTGACTTCCTATTGATTCTACATATTAGTGAGA-- 14127
Illumina Draft  15054 AGTAATCATACTCCATTGACTTCCTATTGATTCTACATATTAGTGAGAGA 15103
                      ************************************************OO

Mt-Molecule 2D  14128 TAAGCAACCCTTGAATGTA-GATTAATCATTACTAGCTTCACGC--ATAA 14174
Illumina Draft  15104 TAAGCAACCCTTGAATGTAAGATTAATCATTACTAGCTTCACGCGCATAA 15153
                      *******************O************************OO****
```

```
Mt-Molecule 2D  14175 TATCCTATTCCACATACTTT-CTTTTCAAAATACCTCTGGTTACTCTTTC 14223
Illumina Draft  15154 TATCCTATTCCACATACTTTTCTTTTCAAAATACCTCTGGTTACTCTTTC 15203
                      ********************O*****************************

Mt-Molecule 2D  14224 CAGTGCTTACGGATCTTCCC----GCAACTCTTGATATTAAGAATACCTC 14269
Illumina Draft  15204 CAGTGCTTACGGATCTTCCCTCCCGCAACTCTTGATATTAAGAATACCTC 15253
                      ********************OOOO**************************

Mt-Molecule 2D  14270 TGGTTGCACTCGAGATTTAAAGACACGTTCTGTCACAAATAAGTAACTTA 14319
Illumina Draft  15254 TGGTTGCACTCGAGATTTAAAGACACGTTCTGTCACAAATAAGTAACTTA 15303
                      **************************************************

Mt-Molecule 2D  14320 TAGTCTGCAAAACATTTGGTACTTTTT--AAATATTTAC-TTTCAACCAC 14366
Illumina Draft  15304 TAGTCTGCAAAACATTTGGTACTTTTTTAAAATATTTACCTTTCAACCAC 15353
                      ***************************OO**********O**********

Mt-Molecule 2D  14367 ATATCCAGTTATGTCTATACCATCCTATATAG--TCAGGCATATTAATGC 14414
Illumina Draft  15354 ATATCCAGTTATGTCTATACCATCCTGTATAGGGTCGGGCATATTAATGC 15403
                      **************************R*****OO**R*************

Mt-Molecule 2D  14415 ATGGTGT--AACGGACATAGACAATTCCAAGAGGCTATTTAATTAATGCT 14462
Illumina Draft  15404 ATGGTGTGTAACGGACATAGACAATTCCAAGAGGCTATTTAATTAATGCT 15453
                      *******OO*****************************************

Mt-Molecule 2D  14463 TGTTAGACATAATACTACATATATTTCCCC-TTCTTAACAAAAATTTTTA 14511
Illumina Draft  15454 TGTTAGACATAATACTACATATATTTCCCCCTTCTTAACAAAAATTTTTA 15503
                      ******************************O*******************

Mt-Molecule 2D  14512 CCATTAACTTTAAAATATTTTATTATAACTTAGTTTTT-CTGCTAAACCC 14560
Illumina Draft  15504 CCATTAACTTTAAAATATTTTATTATAGCTTAGTTTTTTCTGCTAAACCC 15553
                      ***************************R**********O***********

Mt-Molecule 2D  14561 CC-TTACCCCCTTCACGAACATAGATTA----ACTTTATTCTTGTTAAAC 14605
Illumina Draft  15554 CCCTTACCCCC--CACGAACATAGATTAGCTAACTTTATTCTTGTTAAAC 15601
                      **O********II***************OOOO******************

Mt-Molecule 2D  14606 CC-TAAACCAAGAGTCAACTAAACTGAGTTTGTTC--AG-AAAACTTTTT 14651
Illumina Draft  15602 CCCTAAACCAAGAGTCAACTAAACTGAGTTTGTTCGTAGGAAAACTTTTT 15651
                      **O********************************OO**O**********

Mt-Molecule 2D  14652 --CCCCCACTTTTT---AAAATGATAAATAATTTATAAAATCCTATACTA 14696
Illumina Draft  15652 TCCCCCCACTTTTTTTTAAAATGATAAATAATTTATAAAATCCTATACTA 15701
                      OO************OOO*********************************

Mt-Molecule 2D  14697 AGGAAGCCTTATGTATTTTTAAATTTAAACCCTAATTTTT-AATGTATTT 14745
Illumina Draft  15702 AGGAAGCCTTATGTATTTTTAAATTTAAACCCTAATTTTTTAATGTATTT 15751
                      ****************************************O*********

Mt-Molecule 2D  14746 TT---ATC-TGCCAGGGCCACGGAACTTCACGCTTTTAGAGG--AG-AAC 14788
Illumina Draft  15752 TTTTTATCCTGCCAGGGCCACGGAACTTCACGCTTTTAGAGGGGAGGAAC 15801
                      **OOO***O*********************************OO**O***

Mt-Molecule 2D  14789 CCCACCGGCTTTAGGAGCCGGCCA--CTTGGCCAAACC-AAGTGGAAGCT 14835
Illumina Draft  15802 CCCACCGGCTTTAGGAGCCGGCCATTCTTGGCCAAACCCAAGTGGAAGCT 15851
                      ************************OO************O***********

Mt-Molecule 2D  14836 ATGA-CGAAACCATTCGAAAACACCACCCA-CTATTAA-GTCCTAAATCA 14882
Illumina Draft  15852 ATGATCGAAACCATTCGAAAACACCACCCAATTATTAAAGTCCTAAATCA 15901
                      ****O*************************OO******O***********

Mt-Molecule 2D  14883 TGCCTTTATTGACCTACCATCCCCATCCAACATCTCCGCATGATGAAACT 14932
Illumina Draft  15902 TGCCTTTATTGACCTACCATCCCCATCCAACATCTCCGCATGATGAAACT 15951
                      **************************************************
```

```
Mt-Molecule 2D  14933 TTTGGCTCTCTCCTAGGATTATGTTTAATTATCCAGCTAATCACTGGAGT 14982
Illumina Draft  15952 TT-GGCTCTCTCCTAGGATTATGTTTAATTATCCAGCTAATCACTGGAGT 16000
                      **I***********************************************

Mt-Molecule 2D  14983 ATTTTTAGCCATACATTACACCGCCGA-GTAATACTAAGCATTCTCTT-A 15030
Illumina Draft  16001 ATTTTTAGCCATACATTACACCGCCGACGTATCACTA-GCATTCTCTTCA 16049
                      ***************************O***WY****I**********O*

Mt-Molecule 2D  15031 ATCTCTCAT--TTGCCGAAACGTAAACTACGGTTGACTAATCCGCAATAT 15078
Illumina Draft  16050 ATCTCTCATATTTGCCGAAACGTAAACTACGGTTGACTAATCCGCAATAT 16099
                      *********OO***************************************

Mt-Molecule 2D  15079 ACATGCAA-CGGAGCTTCATTGTTTTTCATCTGCCTATACCTTCATGCAA 15127
Illumina Draft  16100 ACATGCAAACGGAGCTTCATTGTTTTTCATCTGCCTATACCTTCATGCAA 16149
                      ********O*****************************************

Mt-Molecule 2D  15128 CCCGAGGCCTTTATTACGGATC-TATCTCTACAAAGAA-CATGAAACATT 15175
Illumina Draft  16150 CCCGAGGCCTTTATTACGGATCCTATCTCTACAAAGAAACATGAAACATT 16199
                      **********************O***************O***********

Mt-Molecule 2D  15176 G-AGTTATCTTACTACTAACCACTATAGCAACAGCATTCGTAGGATATGT 15224
Illumina Draft  16200 GGAGTTATCTTACTACTAACCACTATAGCAACAGCATTCGTAGGATATGT 16249
                      *O************************************************

Mt-Molecule 2D  15225 CCTCCCATGAGGACAAATATCCTTCTGAGG--CAACAGTAATCACAAAC- 15271
Illumina Draft  16250 CCTCCCATGAGGACAAATATCCTTCTGAGGGGCAACAGTAATCACAAACC 16299
                      ******************************OO*****************O

Mt-Molecule 2D  15272 --TTGTCC---ATACCATATGTAGGAGGAACACTTGTAAATTGAATCTGA 15316
Illumina Draft  16300 TCTTGTCCGCCATACCATATGTAGGAGGAACACTTGTAAATTGAATCTGA 16349
                      OO******OOO***************************************

Mt-Molecule 2D  15317 GGAGGCTTCTCAATTGACAACGCCACACTAACCCGATTTT--ACCTTTCA 15364
Illumina Draft  16350 GGAGGCTTCTCAATTGACAACGCCACACTAACCCGATTTTTTACCTTTCA 16399
                      ****************************************OO********

Mt-Molecule 2D  15365 CTTCCTACTCCCATTCATAATCATAGGCTTAAGCATTATACACTTAGTAT 15414
Illumina Draft  16400 CTTCCTACTCCCATTCATAATCATGGGCTTAAGCATTATACACTTAGTAT 16449
                      ************************R*************************

Mt-Molecule 2D  15415 TC-TCCACAAAACAGGATCGAACAACC-AACAGGAATAAACTCCAACTCA 15462
Illumina Draft  16450 TCCTCCACGAAACAGGATCGAACAACCCAACAGGAATAAACTCCAACTCA 16499
                      **O*****R******************O**********************

Mt-Molecule 2D  15463 GACAAAATCCCATTCCACCC-TACTTTTCCTACAAGGACCTGCTAGGAGC 15511
Illumina Draft  16500 GACAAAATCCCATTCCACCCCTACTTTTCCTACAAGGACCTGCTAGGAGC 16549
                      ********************O*****************************

Mt-Molecule 2D  15512 A-T--CAATATTAACAGCATTA-TAACC-TCACACTTTTATACC-AAACC 15555
Illumina Draft  16550 ACTAATAATATTAACAGCATTACTAACCCTCACACTTTTATACCCAAACC 16599
                      *O*OOY****************O*****O***************O*****

Mt-Molecule 2D  15556 -ATTAGGAG--CCGGAAAATTTCACCCCGGCAAACCCACTTTCT------ 15596
Illumina Draft  16600 TATTAGGAGACCCGGAAAATTTCACCCCGGCAAACCCACTTTCTACTCCC 16649
                      O********OO*********************************OOOOOO

Mt-Molecule 2D  15597 CCCCACATTAAGCCAGAGTGATATTTTT-ATTCGCTTATGCCATCCTCCG 15645
Illumina Draft  16650 CCCCACATTAAGCCAGAGTGATATTTTTTATTCGCTTATGCCATCCTCCG 16699
                      ****************************O*********************

Mt-Molecule 2D  15646 ATCTATTCCTAATAAA-TGG-AGGG-TGTTGGCCTTAATACTCTCAATCT 15692
Illumina Draft  16700 ATCTATTCCTAATAAAATGGGAGGGGTGTTGGCCTTAATACTCTCAATCT 16749
                      ****************O***O****O************************
```

```
Mt-Molecule 2D  15693 TAATCCTCCTCATCATACCCCTGGTCCACACATCCAAACAACGAAGCTCA 15742
Illumina Draft  16750 TAATCCTCCTCATCATACCCCTGGTCCACACATCCAAACAACGAAGCTCA 16799
                      **************************************************

Mt-Molecule 2D  15743 ATACACCGCCCAATCAGTAAAATATTATTCTGATACTTAATCTCCACTAT 15792
Illumina Draft  16800 ATACACCGCCCAATCAGTAAAATATTATTCTGATACTTAATCTCCACTAT 16849
                      **************************************************

Mt-Molecule 2D  15793 TTTCATCCTCACATGAATTGGGG-ACAACCAGTAGAACCCC-ATTCACGG 15840
Illumina Draft  16850 TTTCATCCTCACATGAATTGGGGGACAACCAGTAGAACCCCCATTCATCT 16899
                      ***********************O*****************O*****YSK

Mt-Molecule 2D  15841 TAATCGGCCAAACAAC-TCAGTAATTTATTTCCTAATTATTACAGTCCTA 15889
Illumina Draft  16900 TAATCGGCCAAACAGCCTCAGTAATTTATTTCCTAATTATTACAGTCCTA 16949
                      **************R*O*********************************

Mt-Molecule 2D  15890 ATTC-TATTACTGGCACTAT-GAAAATAAA-TATTAAAC-GATAGTCTC- 15934
Illumina Draft  16950 ATTCCTATTACTGGCACTATAGAAAATAAAATATTAAACTGATAGTCTCA 16999
                      ****O***************O*********O********O*********O

Mt-Molecule 2D  15935 CTCAAGAAAAGAGGACCTTTCCC-TATCCC-GGTTCCCAGAAC-GGAGTT 15981
Illumina Draft  17000 CTCAAGAAAAGAGGACCTTTCCCCTATCCCCGGTTCCCAGAACCGGAGTT 17049
                      ***********************O******O************O******

Mt-Molecule 2D  15982 TTAAATAAACTATTTCTTGACATCACAAACCTC-ACCAACCGGAG--GGA 16028
Illumina Draft  17050 TTAAATAAACTATTTCTTGACATCACAAACCTCTACCAACCGGAGTGGGA 17099
                      *********************************O***********OO***

Mt-Molecule 2D  16029 AAAA-GTGATTGCTAATCACTAAAATCAGGGTTAAACACCTGACCCCC-TC 16076
Illumina Draft  17100 AAAAAGTGATTGCTAATCACTAAAATCAGGGTTAAACACCTGACCCCCCTC 17149
                      ****O******************************************O**

Mt-Molecule 2D  16077 ATTAATGCAGGCGGCATGCCGCCTTATATTTATATA--CTATACTCTAGT 16124
Illumina Draft  17150 ATTAATGCAGGCGGCATGCCGCCTTATATTTATATATACTATACTCTAGT 17199
                      ************************************OO************

Mt-Molecule 2D  16125 ACGTCCTTCCCCC-TCC-TATGTATATCGTGCATTCCTTCTCGACCTCAC 16172
Illumina Draft  17200 ACGTCCTTCCCCCCTCCCTATGTATATCGTGCATTCCTTCTCGACCTCAC 17249
                      *************O***O********************************

Mt-Molecule 2D  16173 GAGTAATCAT-CATATATT-A-TTCCTATTGATTCTACATATTA-TGAGA 16218
Illumina Draft  17250 GAGTAATCATAC-TCCATTGACTTCCTATTGATTCTACATATTAGTGAGA 17298
                      **********O*I*MY***O*O**********************O*****

Mt-Molecule 2D  16219 GATG-GCAACCCT-GAATGTAAGATTAATCGT-ACTAGCTTCACGCGCAT 16265
Illumina Draft  17299 GATAAGCAACCCTTGAATGTAAGATTAATCATTACTAGCTTCACGCGCAT 17348
                      ***RO********O****************R*O*****************

Mt-Molecule 2D  16266 AATATCCTATTCCACATACTTTTCTTTTTCAAAATACC----GTTACT-T 16310
Illumina Draft  17349 AATATCCTATTCCACATACTTTTCTTTT-CAAAATACCTCTGGTTACTCT 17397
                      ****************************I*********OOOO******O*

Mt-Molecule 2D  16311 TTCCAGTGCTTACATGGATCTTCCCTCCCGCAAATTTTTGATA---AGAA 16357
Illumina Draft  17398 TTCCAGTGCTTAC--GGATCTTCCCTCCCGCAA-CTCTTGATATTAAGAA 17444
                      *************II******************IY*Y******OOO****

Mt-Molecule 2D  16358 TAC-TCTGGTTGCAC-CGAGATTTAAAGACACGTTCTGTCACAAATAAGT 16405
Illumina Draft  17445 TACCTCTGGTTGCACTCGAGATTTAAAGACACGTTCTGTCACAAATAAGT 17494
                      ***O***********O**********************************

Mt-Molecule 2D  16406 AACTTATAGTCTGCAAAACATTTGGTACTTTTT-AAAATATTTACCTTTC 16454
Illumina Draft  17495 AACTTATAGTCTGCAAAACATTTGGTACTTTTTTAAAATATTTACCTTTC 17544
                      *********************************O****************
```

```
Mt-Molecule 2D  16455 AACCA--TATCCAGTTATGTCTATACCATCCTGTATAGGGTCGGCATAT 16502
Illumina Draft  17545 AACCACATATCCAGTTATGTCTATACCATCCTGTATAGGGTCGGCATAT 17594
                      *****OO*******************************************

Mt-Molecule 2D  16503 TAATGCATGGTGTGTAACGGACATAGACAATTCCA-GAGGCTATTTAATT 16551
Illumina Draft  17595 TAATGCATGGTGTGTAACGGACATAGACAATTCCAAGAGGCTATTTAATT 17644
                      ***********************************O**************

Mt-Molecule 2D  16552 AATGCTTGTT-G-TATAATACTACATATATTTCCCC-TTCTTAACAAAA- 16597
Illumina Draft  17645 AATGCTTGTTAGACATAATACTACATATATTTCCCCCTTCTTAACAAAAA 17694
                      **********O*OY**********************O************O

Mt-Molecule 2D  16598 -TTTTACCATTAACTTTAAAATATTTTATTATAGC--AGTTTTT-CTGCT 16643
Illumina Draft  17695 TTTTTACCATTAACTTTAAAATATTTTATTATAGCTTAGTTTTTTCTGCT 17744
                      O**********************************OO*******O*****

Mt-Molecule 2D  16644 AAACCCC--TTACCCCC-ACGAACATAGATTAGCTAACTTTATTCT-GTT 16689
Illumina Draft  17745 AAACCCCCCTTACCCCCCACGAACATAGATTAGCTAACTTTATTCTTGTT 17794
                      *******OO********O****************************O***

Mt-Molecule 2D  16690 AAACCC-TAAACCAAGAGTCAA-TAAACTGAGTTTGTTCGTAGGAAAACT 16737
Illumina Draft  17795 AAACCCCTAAACCAAGAGTCAACTAAACTGAGTTTGTTCGTAGGAAAACT 17844
                      ******O***************O***************************

Mt-Molecule 2D  16738 TTTT---CCCCACTTTTT----AAATGATAAATAATTTATAAAATCCTAT 16780
Illumina Draft  17845 TTTTTCCCCCCACTTTTTTTTAAAATGATAAATAATTTATAAAATCCTAT 17894
                      ****OOO***********OOOO****************************

Mt-Molecule 2D  16781 ACTAAGGAAGCCTTATGCGTTTT-AAACATTAAGT---ATTTTT---ACA 16823
Illumina Draft  17895 ACTAAGGAAGCCTTATGCATTTTTAAACATTAAATCTTATTTTTTTTACA 17944
                      ******************R****O*********R*OOO******OOO***

Mt-Molecule 2D  16824 T-ATTTTTAC--AAAAATAAAGCATTTT-ACATAAAATATAAGGGCCCGT 16869
Illumina Draft  17945 TTATTTTTACCAAAAAATAAAGCATTTTTACATAAAATATAAGGGCCCGT 17994
                      *O********OO****************O*********************

Mt-Molecule 2D  16870 ACCTACAACCACACCATGTATAATTTATTTCTTTTT--CATAAAA-TAAA 16916
Illumina Draft  17995 ACCTACAACCACACCATGTATAATTTATTTCTTTTTTCCATAAAAATAAA 18044
                      ************************************OO*******O****

Mt-Molecule 2D  16917 AGCACTGAAA-TGCCTCTTACCATTTACAATAAAA 16950
Illumina Draft  18045 -GCACTGAAAATGCCTCTTACCATTTACAATAAAA 18078
                      I*********O************************
```
