# Supplementary material for: Evidence of two deeply divergent co-existing mitochondrial genomes in the Tuatara reveals an extremely complex genomic organization
Source: Commun Biol. 2021 Jan 29;4:116. doi: 10.1038/s42003-020-01639-0 (PMC7846811; doi:10.1038/s42003-020-01639-0)
Supplement: Supplementary file 2 — Description of Additional Supplementary Files [file 42003_2020_1639_MOESM2_ESM.pdf]

Description of Additional Supplementary Files

File Name: Supplementary Data 1
Description: Oxford Nanopore reads for LAI molecule 1 (M1). File contains 114 Oxford Nanopore mtDNA reads that were assigned to M1 based on a 5% difference cut off (see Methods: Oxford Nanopore data and evaluation of LAI). File is in fasta format.

File Name: Supplementary Data 2
Description: Oxford Nanopore reads for LAI molecule 2 (M2). File contains 32 Oxford Nanopore mtDNA reads that were assigned to M2 based on a 5% difference cut off (see Methods: Oxford Nanopore data and evaluation of LAI). File is in fasta format.
