# Supplementary material for: Evidence of two deeply divergent co-existing mitochondrial genomes in the Tuatara reveals an extremely complex genomic organization
Source: Commun Biol. 2021 Jan 29;4:116. doi: 10.1038/s42003-020-01639-0 (PMC7846811; doi:10.1038/s42003-020-01639-0)
Supplement: Supplementary file 5 — Reporting Summary [file 42003_2020_1639_MOESM5_ESM.pdf]

nature research

Corresponding author(s): Macey et al. 2020

Last updated by author(s): May 6, 2020

Reporting Summary

Nature Research wishes to improve the reproducibility of the work that we publish. This form provides structure for consistency and transparency in reporting. For further information on Nature Research policies, seeAuthors & Referees and theEditorial Policy Checklist .

Statistics

For all statistical analyses, confirm that the following items are present in the figure legend, table legend, main text, or Methods section.

n/a	Confirmed	
☐	☒	The exact sample size (*n*) for each experimental group/condition, given as a discrete number and unit of measurement
☒	☐	A statement on whether measurements were taken from distinct samples or whether the same sample was measured repeatedly
☐	☒	The statistical test(s) used AND whether they are one- or two-sided<br>*Only common tests should be described solely by name; describe more complex techniques in the Methods section.*
☒	☐	A description of all covariates tested
☐	☒	A description of any assumptions or corrections, such as tests of normality and adjustment for multiple comparisons
☒	☐	A full description of the statistical parameters including central tendency (e.g. means) or other basic estimates (e.g. regression coefficient) AND variation (e.g. standard deviation) or associated estimates of uncertainty (e.g. confidence intervals)
☐	☒	For null hypothesis testing, the test statistic (e.g. *F*, *t*, *r*) with confidence intervals, effect sizes, degrees of freedom and *P* value noted<br>*Give P values as exact values whenever suitable.*
☒	☐	For Bayesian analysis, information on the choice of priors and Markov chain Monte Carlo settings
☒	☐	For hierarchical and complex designs, identification of the appropriate level for tests and full reporting of outcomes
☒	☐	Estimates of effect sizes (e.g. Cohen's *d*, Pearson's *r*), indicating how they were calculated

Our web collection on statistics for biologists contains articles on many of the points above.

Software and code

Policy information about availability of computer code

Data collection	Illumina assemblies were done in Geneious v10.2.4 and MaSuRCA with mapping in Minimus and evaluations done with Jellyfish. Oxford Nanopore 2D reads were extracted using Nanopolish with 1D reads done with Guppy. Blastn and Megablast were used to search data and backmapping done in Geneious v10.2.4. PacBio reads were assembled in CANU v1.8. Alignments were done in Geneious using MUSCLE v3.8.425 and MacVector v8.0 using ClustalW v1.4.
Data analysis	Phylogenetic estimates were constructed in PAUP* v4.0, with alternative trees made in MacClade v4.03. Selection analyses were done with PAML-CodeML using the BEB method implemented in ETE3, and FEL implemented in HyPhy. Protein structure simulations applied the ConSurf Server to the UNIREF-90 database, based on the MAFFT-L-INS-i aligner, HHPred and MODELLER. PyMOL was used for structure visualizations.

For manuscripts utilizing custom algorithms or software that are central to the research but not yet described in published literature, software must be made available to editors/reviewers. We strongly encourage code deposition in a community repository (e.g. GitHub). See the Nature Research guidelines for submitting code & software for further information.

Data

Policy information about availability of data

All manuscripts must include a data availability statement. This statement should provide the following information, where applicable:

- Accession codes, unique identifiers, or web links for publicly available datasets
- A list of figures that have associated raw data
- A description of any restrictions on data availability

All data are freely available. The Tuatara Genome Consortium Project Whole Genome Shotgun and genome assembly are registered under the umbrella BioProject PRJNA418887. The Lady Alice Island (LAI) sample is a male and NCBI Biosample SAMN08793959 with the four Stephens Island samples all being female: (SI-1) SAMN10598677, (SI-2) SAMN10598679, (SI-3) SAMN10598680, and (SI-4) SAMN00855319 with published transcriptome data in SRA0516479. New DNA sequences generated in this study are deposited as GenBank MN864228– MN864230 and Sequence Read Archive (SRA) PRJNA445603. Published transcriptome sequences used in this study are from SRA SRA051647 and the complete mt-genome from that transcriptome is deposited as a Third Party Annotation in the DDBJ/ENA/

GenBank databases as TPA: BK012001.

Field-specific reporting

Please select the one below that is the best fit for your research. If you are not sure, read the appropriate sections before making your selection.

☐ Life sciences ☐ Behavioural & social sciences ☒ Ecological, evolutionary & environmental sciences

For a reference copy of the document with all sections, see nature.com/documents/nr-reporting-summary-flat.pdf

Ecological, evolutionary & environmental sciences study design

All studies must disclose on these points even when the disclosure is negative.

Study description	This is the study of mitochondrial genomes in the Tuatara. One male from Lady Alice Island is compared with four female samples from Stephens Island. Long-read and short read sequencing is conducted to provide a robust picture of Tuatara mt-genomics.
Research sample	One male Tuatara (Sphenodon punctatus) from Lady Alice Island New Zealand, and four female Tuatara samples from Stephens Island New Zealand encompassing the extremes of genetic diversity for this species.
Sampling strategy	Sampling was undertaken using venipuncture or necropsy. Because this species is special to Maori and highly protected only one wild sample is used from Lady Alice Island. Remaining samples from Stephens Island are from St. Louis Zoo. No animals were sacrificed in this study. Inclusion of published transcriptome data from a Stephens Island individual further accented this report.
Data collection	DNA sequencing was conducted via standard methods.
Timing and spatial scale	Samples were collected from Lady Alice Island in 2011 and St. Loius Zoo animals from Stephens Island in 2002 and 2018.
Data exclusions	No data are excluded from this study.
Reproducibility	Data reproducibility was verified using repeat sequencing and independent analyses using alternative pipelines.
Randomization	There was no need to randomise our study given the focus on genomic and population genomics
Blinding	Blinding was irrelevant given the focus on genomic and population genomics

Did the study involve field work? ☒ Yes ☐ No

Field work, collection and transport

Field conditions	Data not available, but field site is a temperate offshore Islands in New Zealand
Location	Lady Alice, New Zealand
Access and import/export	The Lady Alice Island sample was collected under Victoria University of Wellington Animal Ethics approvals 2006R12; 2009R12; 2012R33; 22347 and held and used under permits 45462-DOA and 32037-RES 32037-RES issued by the New Zealand Department of Conservation. Import/export of the Lady Alice Island sample followed CITES guidelines and described in Methods. St. Louis Zoo animals were handled under zoo protocol.
Disturbance	Animals were handled minimally and returned to the site of capture for release or zoo.

Reporting for specific materials, systems and methods

We require information from authors about some types of materials, experimental systems and methods used in many studies. Here, indicate whether each material, system or method listed is relevant to your study. If you are not sure if a list item applies to your research, read the appropriate section before selecting a response.

Materials & experimental systems

n/a	Involved in the study	
☒	☐	Antibodies
☒	☐	Eukaryotic cell lines
☒	☐	Palaeontology
☐	☒	Animals and other organisms
☒	☐	Human research participants
☒	☐	Clinical data

Methods

n/a	Involved in the study	
☒	☐	ChIP-seq
☒	☐	Flow cytometry
☒	☐	MRI-based neuroimaging

Animals and other organisms

Policy information about studies involving animals; ARRIVE guidelines recommended for reporting animal research

Laboratory animals	N/A
Wild animals	One adult male Tuatara, (Sphenodon punctatus) from Lady Alice Island was captured and a blood sample taken using established venipuncture approaches.
Field-collected samples	N/A
Ethics oversight	A sample was collected under Victoria University of Wellington Animal Ethics approvals 2006R12; 2009R12; 2012R33; 22347. St. Louis Zoo policy and protocol were applied by zoo staff.

Note that full information on the approval of the study protocol must also be provided in the manuscript.
